# Polypeptide-Based Systems: From Synthesis to Application in Drug Delivery

**DOI:** 10.3390/pharmaceutics15112641

**Published:** 2023-11-20

**Authors:** Mariia Stepanova, Alexey Nikiforov, Tatiana Tennikova, Evgenia Korzhikova-Vlakh

**Affiliations:** 1 Institute of Macromolecular Compounds, Russian Academy of Sciences, Bolshoy pr. 31, 199004 St. Petersburg, Russia; maristepanova@hq.macro.ru (M.S.); anikiforov71@gmail.com (A.N.); 2 Institute of Chemistry, Saint-Petersburg State University, Universitetskiy pr. 26, Petergof, 198504 St. Petersburg, Russia

**Keywords:** polypeptides, polypeptide copolymers, polypeptide particles, self-assembly, hydrogels, drug delivery systems

## Abstract

Synthetic polypeptides are biocompatible and biodegradable macromolecules whose composition and architecture can vary over a wide range. Their unique ability to form secondary structures, as well as different pathways of modification and biofunctionalization due to the diversity of amino acids, provide variation in the physicochemical and biological properties of polypeptide-containing materials. In this review article, we summarize the advances in the synthesis of polypeptides and their copolymers and the application of these systems for drug delivery in the form of (nano)particles or hydrogels. The issues, such as the diversity of polypeptide-containing (nano)particle types, the methods for their preparation and drug loading, as well as the influence of physicochemical characteristics on stability, degradability, cellular uptake, cytotoxicity, hemolysis, and immunogenicity of polypeptide-containing nanoparticles and their drug formulations, are comprehensively discussed. Finally, recent advances in the development of certain drug nanoformulations for peptides, proteins, gene delivery, cancer therapy, and antimicrobial and anti-inflammatory systems are summarized.

## 1. Introduction

Traditional routes of free drug administration often demonstrate low therapeutic efficacy, which may be due to low stability, poor bioavailability, or high systemic or specific toxicity of some drugs. These drawbacks can be overcome by the use of drug delivery systems [1], among which inorganic and organic nanoparticles (NPs) can be applied. Unlike inorganic NPs, which can be quite toxic, have poor surface functionality, low drug capacity (except mesoporous particles), and unclear mechanisms of elimination from the body [2,3], organic NPs, e.g., liposomes, lipids, and polymeric NPs, are devoid of these bottlenecks [4]. In turn, compared to NPs derived from small organic molecules, polymeric delivery systems can provide higher stability and prolonged drug release through the gradual or triggered degradation of the polymer [5]. In this regard, a large number of biocompatible and biodegradable natural and synthetic (co)polymers have been studied for the development of drug delivery systems, including their targeted and stimuli-responsive variants [6,7]. Such classes of biodegradable polymers include natural polysaccharides [8], aliphatic hydroxycarboxylic acid polyesters (or polyhydroxyalkanoates) [9,10], poly(trimethyl carbonates) [11], polypeptides [12], poly(organo)phosphazenes [13], etc., as well as their copolymers with nondegradable biocompatible polymers such as poly(ethylene glycol), poly(N-isopropylacrylamide), synthetic glycopolymers, etc., are widely investigated for the development of nanomedicines [6,14].

Among other synthetic biodegradable copolymers, polypeptides (or poly(amino acids)) have a number of distinct advantages [15,16]. According to the nomenclature recommended by IUPAC, polypeptides are macromolecules consisting of more than 20 amino acid residues [17]. However, the term is sometimes applied to shorter oligomeric sequences obtained by the polymerization technique and having a molecular weight distribution, in contrast to peptides with a specific primary sequence [18]. This class of macromolecules represents bioinspired polymers with such important properties in biomedicine as biodegradability, biocompatibility, and a variety of side chain functionalities, making polypeptides very attractive for the development of scaffolds, drug delivery systems, and vaccines. In addition, the diversity of natural and unnatural amino acids makes it possible to vary the composition of polypeptides within a wide range, giving them anionic, cationic, neutral, amphiphilic, or hydrophobic properties and different biodegradability. Furthermore, similar to other synthetic and natural polymers, polypeptides can exhibit pH-, enzyme-, reduction-, and even temperature-sensitive properties [19]. Unlike many synthetic copolymers, synthetic polypeptides can form stable secondary structures such as *α*-helixes and *β*-sheets [20,21]. This property affects self-assembly behavior and underlies the properties of drug delivery systems. Finally, varying the functional groups of side chains allows the conjugation of drugs and vectors for targeted delivery [22]. Overall, polypeptide-based/containing delivery systems can be considered versatile platforms for the delivery of drugs with different structures, physicochemical properties, and biological properties.

In this review, we focus on analyzing the background and current advances in the development and application of polypeptide systems for drug delivery. In contrast to the existing reviews that focus mainly on synthetic aspects [23,24,25] or certain biomedical applications [15,19,26,27], this review addresses important issues in addition to synthetic aspects, such as the analysis of polymers and nanoparticle types based on/containing polypeptides, methods for their preparation, the self-assembly features of amphiphilic polypeptides and their copolymers, drug loading, and the properties of the systems, such as stability, degradability, cellular uptake capacity, cytotoxicity, and immunogenicity. Finally, the developed drug delivery systems based on polypeptides and their copolymers were summarized and discussed. In addition to delivery systems in nanoparticle format, attention has also been paid to hydrogels as drug delivery systems.

Thus, this review provides a comprehensive overview of polypeptides and their copolymers, NPs, and hydrogels based on them, as well as the physicochemical and biological properties of polypeptide-containing (nano)materials with respect to their application as delivery systems.

Due to the fact that different abbreviations for the same polymers are used in numerous original articles, a uniform system for polymer abbreviations, which may differ from the abbreviations introduced in the original sources, is applied in this review to avoid confusion within this text. In particular, the standard three-letter abbreviations recommended by IUPAC were used for all L-amino acids. The abbreviations for amino acids, polymers, drugs, and other compounds used in this paper are summarized in the Abbreviations part.

## 2. Synthetic Polypeptides and Their Copolymers

### 2.1. Homopolypeptides

**Figure 1:**
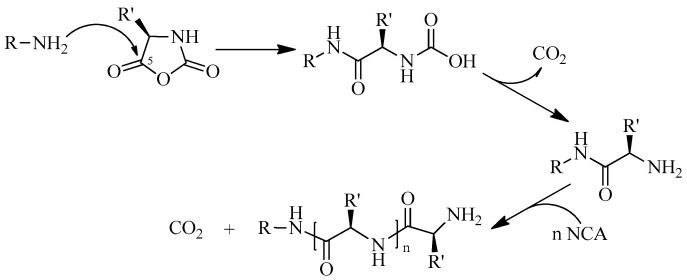
Normal amine mechanism of NCA polymerization.

Currently, the most common approach to synthesizing polypeptides is the ring-opening polymerization (ROP) of N-carboxyanhydrides (NCA) of α-amino acids [28]. Traditionally, linear primary amines are the most widely used initiators, for which the reaction rate of the initiation step is much higher than that of the chain growth step. This leads to the production of polypeptides with relatively low dispersity (*Đ*). The reaction is believed to proceed by the so-called “normal amine mechanism” (NAM), involving a nucleophilic attack of the amino group on the 5-CO carbon atom of NCA [29]. The terminal amino group formed in the process of decarboxylation of intermediate carbamic acid begins to act as an initiating group in the chain growth stages (Figure 1). NAM can be complicated by the presence of many other nucleophilic compounds or bases, such as impurities of any amines, alcohols, or water remaining in the NCA and solvents. These impurities can serve as initiators of NCA ROP and lead to the formation of polymers with a bimodal or broad molecular weight distribution.

Usually, NAM is well realized when using polar solvents for polymerization (*N*,*N*-dimethylformamide (DMF), nitrobenzene). In this case, the reaction follows first-order kinetics. Substitution of polar solvents with non-polar solvents (1,4-dioxane, tetrahydrofuran (THF), and benzene) changes the polymerization kinetics. For example, the ROP of Glu(OBzl) NCA in a nonpolar solvent gives a complex kinetic function that has a break at a conversion of 20–40%. The latter is explained by the change in conformation of the polypeptide from a disordered structure to an α-helix [30].

**Figure 2:**
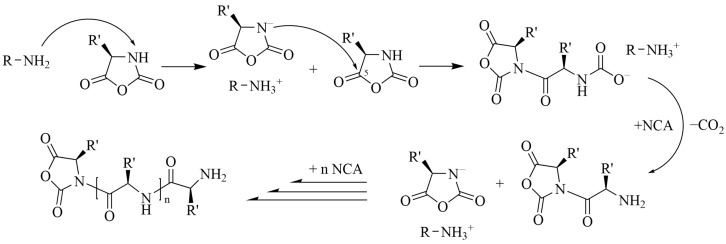
Activated monomer mechanism of NCA polymerization.

When strong bases are used as initiators, e.g., alkoxides of alkali metals or tertiary amines, the “activated monomer mechanism” (AMM) takes place. Initially, the NH group of NCA is deprotonated to form its anion, which acts as a direct initiator of the polymerization reaction (Figure 2). In the case of secondary amines or sterically hindered primary amines, both normal amine and activated monomer mechanisms are believed to proceed in parallel to some extent [31]. It can be noted that the use of strong inorganic bases (NaOMe, NaOH), unlike primary amines, allows to obtain polypeptides with extremely high values of degree of polymerization (DP), even up to 11,000 [32,33].

In order to improve control over NCA ROP, a number of methods have been developed to produce well-defined linear polypeptides. One of the proposed approaches is to change the pressure and temperature of the NCA polymerization reaction. Lowering the temperature (usually to 0 °C) slows down the side reactions compared to the chain growth reaction. This is attributed to the fact that at lower temperatures, the activation barrier of the chain growth reaction becomes lower than that of the side reactions [34,35]. Furthermore, a number of authors attribute a significant decrease in *Đ* values to the increased stability of carbamic acids at low temperatures, which leads to a greater difference in the rates of initiation and chain growth reactions [36].

Lowering the pressure in the reaction system to ~1 × 10^−5^–1 × 10^−6^ bar favors the rapid removal of forming carbon dioxide. When performing ROP in DMF, CO_2_ can interact with this solvent, resulting in the formation of formaldehyde and dimethylamine, leading to side reactions including chain termination [36]. In addition, the acceleration of CO_2_ removal hinders the deprotonation of NCA [37,38], thus avoiding the reaction *via* the AMM side reaction pathway and the formation of isocyanates, which also leads to chain termination.

**Figure 3:**
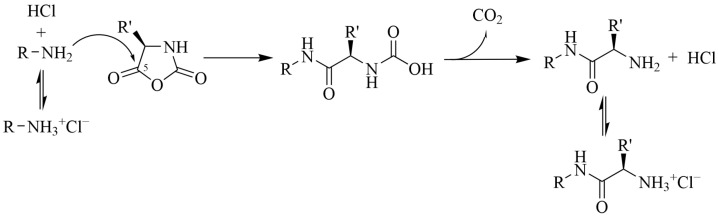
Synthesis of polypeptides using amine-hydrochlorides as initiators in NCA ROP.

Another approach to avoid deprotonation of NCA and realize exactly AMM is the use of hydrochlorides of primary amines, first proposed by Schlaad et al. [37]. The presence of additional protons in the system allowed the protonation of the formed NCA anions faster than the reaction initiation step (Figure 3) [39]. However, increasing the equilibrium concentration of free amine required increasing the reaction temperature and time. Furthermore, it was not possible to achieve high conversions, as the molecular masses of the obtained polypeptides exceeded the calculated ones by 20–30%.

**Figure 4:**
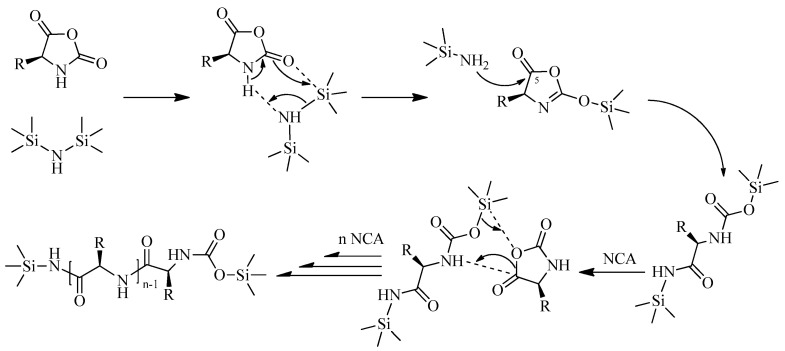
Proposed mechanism for polypeptide synthesis using HMDS initiator.

Another type of initiator that allows the preparation of polypeptides with controlled molecular weight characteristics are derivatives of silazanes, in particular, hexamethyldisilazane (HMDS). Its application was first described by Lu et al. for the polymerization of Glu(OBzl) NCA [40]. In accordance with the proposed mechanism, HMDS, interacting with the NCA molecule, deprotonates the 3-N nitrogen atom to form trimethylsilazane, which in turn conducts a nucleophilic attack on the 5-CO carbon atom. This leads to the opening of the NCA ring with the formation of trimethylsilylcarbamate and trimethylsilazane terminal groups (Figure 4). The subsequent growth of the polypeptide chain is associated with the transfer of the trimethylsilylcarbamate group to another monomer molecule. This approach allows obtaining polypeptides with *Đ*~1.2, and the use of various *N*-trimethylsilyl amines as initiators provides the formation of the desired terminal groups in the synthesized polypeptides [29].

**Figure 5:**
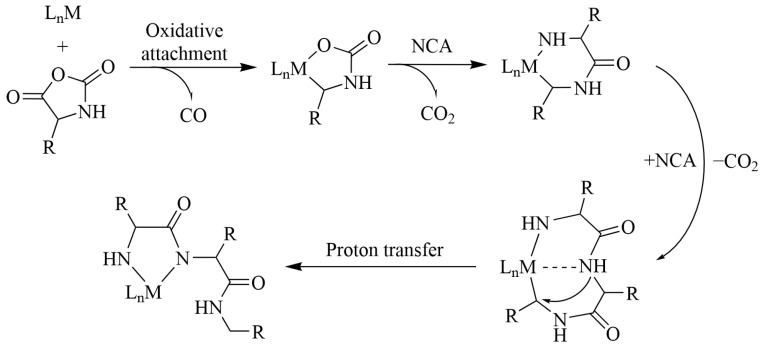
Ring-opening polymerization of NCA using transition metals zero-valence cyclooctadiene complexes.

Deming et al. proposed the use of transition metal zero-valence cyclooctadiene complexes M(COD)(bipy) (COD—1,5-cyclooctadiene; bipy—2,2′-bipyridine), M(COD)(R_3_P)_2_, and M(R_3_P)_4_ (M = Co, Ni, Fe, Pt, and Pd) in the NCA ROP [41,42,43]. These organometallic compounds act as both catalysts and initiators in NCA polymerization and allow the production of polypeptides with narrow molecular weight distributions and controlled molecular weights (500 < M_n_ < 500,000) [29]. When interacting with NCA monomers, their oxidative attachment through anhydride bonds occurs with the formation of a six-membered amido-alkyl metallocycle (Figure 5). The latter, interacting with the second monomer molecule, forms a five-membered amido-amidate metallocycle complex, which directly participates in the subsequent stages of chain growth due to the nucleophilic attack of the amide NH-group on the 5-CO carbon atom of NCA.

In addition, organometallic complexes of Pt, Ir, and Ru have been reported as catalysts and initiators of ROP [44,45]. Despite the well-defined nature of the resulting polypeptides, ROP using such compounds has not been widely used due to the lack of commercially available organometallic compounds that could be used for routine synthesis.

An overview of recent advances in the synthesis of polypeptides by ROP from α-amino acid NCAs is also presented elsewhere [46].

### 2.2. Copolypeptides

During the whole time of NCA chemistry research, applying traditional approaches to synthesis, a considerable variety of amino acid homopolymers have been successfully obtained. Blout et al. in the 1950s–1960s investigated in detail the influence of reaction temperature, solvents, initiators, and monomer concentrations on the molecular weight characteristics of the obtained poly-*γ*-benzyl-L-glutamates, poly-*ε*-carbobenzyloxy-L-lysine, and poly-L-serine [47,48]. In general, taking into consideration all developed synthetic approaches, the preparation of homopolypeptides with given molecular weight characteristics does not cause difficulties today. A somewhat more difficult task is the preparation of copolymers of amino acids and block copolymers consisting of polypeptide fragments.

**Figure 6:**
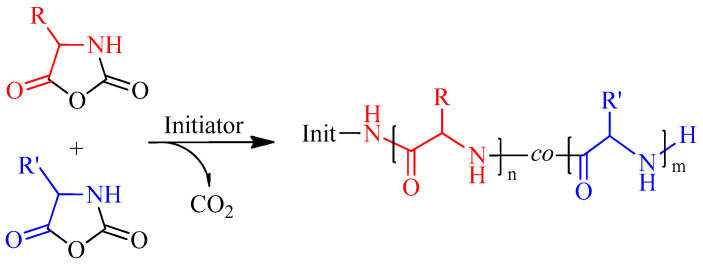
Simplified scheme for the synthesis of copolypeptides by copolymerization of different NCAs.

Conventional synthetic approaches are mainly used in the preparation of copolypeptides. In particular, for the synthesis of statistical/random copolypeptides, a reaction mixture of two or more NCA amino acids is subjected to ROP (Figure 6). Despite some differences in the composition of the initial reaction systems and the resulting copolypeptides, this approach has been successfully used by many authors [49,50,51,52]. To date, the synthesis of numerous copolypeptides by copolymerization of two or more NCAs of different amino acids has been reported. For example, the following combinations can be found in the literature: random/statistical copolymers of Glu(OBzl) and Val [53], Leu and Val [54], Lys(Z) and Asp(OBzl) [55], Ala and Val [56], Lys(Z) and Val [57], Ala and Sar [58], Ala and Pro [59], Lys and Phe [51], Glu and Phe [51,60], Lys and Aib [52], Arg and Lys [61], Lys and Ile [62], Lys and His [63], as well as terpolymers of Leu, Asp(OBzl) and Val [64], Glu, Ala and Tyr [65], Lys, Glu and Ile/Phe [66], and Glu(OBzl), Lys(Z) and Tyr(Bzl) [67].

The final composition of the copolypeptide depends on the reactivity of the monomers [29,68]. The latter, in turn, is influenced by the reaction conditions and especially by the solvent in which the ROP is carried out. According to literature data, the most uniform distribution of amino acid residues in the polypeptide chain is achieved when polymerization is carried out in nonpolar solvents, such as benzene or a mixture of benzene and dichloromethane [69]. In addition, there are suggestions that the reactivity of amino acids decreases with an increasing probability of amino acid participation in α-helix formation. It was also found that racemic mixtures of NCAs polymerize slower than optically pure ones [70]. At the same time, no effect of the initiator has been observed on the polypeptide composition.

In addition to copolymerization, modification of the side chains of homopolypeptides is also a widely used strategy to introduce new functionalities and produce copolypeptides. It can be the modification with other amino acids [71,72] or small functional moieties such as hydrophobic fragments [73,74], sugar units [75,76], OEG and pyridinium tetrafluoroborate [77], allyl or propargyl groups [78], etc.

Finally, copolypeptides can be obtained by partial deprotection of side-protecting groups of various homopolypeptides [79]. In particular, Higuchi et al. proposed the synthesis of poly[(*γ*-methyl-L-glutamate)-*co*-(L-glutamic acid)] *via* ROP of *γ*-methyl-L-glutamate NCA, followed by partial deprotection with trifluoroacetic acid. The copolymer containing 30% of Glu-units was obtained by the developed approach.

### 2.3. Block-Polypeptides and Hybrid Polypeptide-Containing Block-Copolymers

**Figure 7:**
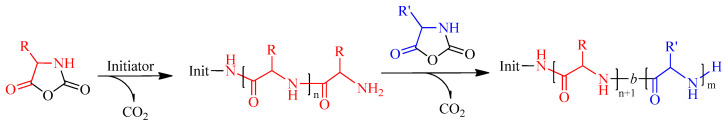
Scheme for the preparation of block-polypeptides by sequential polymerization of different NCAs.

In the synthesis of block-polypeptides, the most common approach is to initially prepare an oligo- or polypeptide and use it as a macroinitiator in the ROP of the NCA of the other amino acid. A general outline of such a synthetic approach is shown in Figure 7.

In most cases, the initially obtained homopolypeptide macroinitiator is isolated from the reaction mixture and purified, and after that, the synthesis of the block copolymer is performed [18,80,81,82,83]. However, there are publications in which the interaction of the obtained macroinitiator with the following NCA is carried out without its preliminary isolation. For example, Su et al. obtained a series of PLys_30_-*b*-PPhe_15–45_ by the “one-pot” method using the NAM polymerization technique in vacuum [84]. A similar “one-pot” method was applied by Holowka et al. in the synthesis of block-polypeptides of Leu with Lys(Z) or Glu(OBzl) [85], and Jan et al. in the synthesis of PLys-*b*-PCys(Bzl) [86] and PLys-*b*-PThr [87].

In the preparation of hybrid block copolymers, the most common approach is the polymerization of NCAs on the terminal amino group of a non-polypeptide macroinitiator. For example, NH_2_-terminated PEG is one of the most common macroinitiators for the preparation of such hybrid block copolymers. At present, a wide range of PEGylated block copolymers, including those containing a random polypeptide block, are known and widely used in the development of drug delivery systems (see Section 5). Table 1 summarizes a number of known PEGylated polypeptide-containing block copolymers, their molecular weight characteristics, and methods of preparation. Natural and synthetic glycopolymers are also widely used as hydrophilic biocompatible building blocks to produce polypeptide-containing block copolymers and their based delivery with reduced cytotoxicity and macrophage uptake (Table 1).

**Figure 8:**
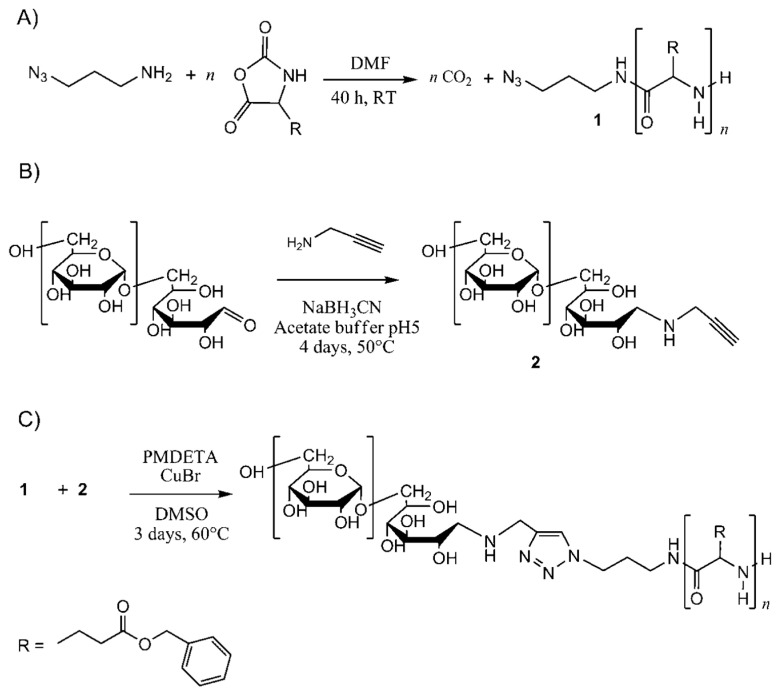
Synthesis of dextran-*block*-poly(*γ*-benzyl-L-glutamate) by “click chemistry”: (**A**) Synthesis of azido-terminated PGlu(OBzl) by ROP using 1-azido-3-aminopropane as initiator, (**B**) Functionalization of dextran by reductive amination with propargylamine to produce a terminal alkyne group, (**C**) Block coupling of PGlu(OBzl) and modified dextran by “click chemistry” to produce dextran-*b*-PGlu(OBzl). Reproduced without changes with permission of John Wiley and Sons, Inc. from [90].

Besides ROP initiated with an amino-terminated macroinitiator, coupling methods such as solid-phase peptide synthesis or “click chemistry” can also be used to produce block-copolymers [88,89,90]. The latter is possible through ROP NCA initiated using 1-azido-3-aminopropane to synthesize an azido-terminated polypeptide, followed by 1,3-dipolar cycloaddition with an alkyne group previously introduced into a polymer of a different nature, catalyzed by copper(I) compounds (Figure 8).

In addition, examples of the preparation of block copolypeptides containing blocks of other synthetic polymers, such as polyethylene, poly(N-isopropylacrylamide), and polyoxazoline, are reported (Table 1). Utilizing the approaches described above, but using bifunctional polymers as a macroinitiator or coupling agent, it is possible to obtain amphiphilic triblock copolymers. Such hybrid triblock copolymers are usually synthesized using PEG with two terminal amino groups as a macroinitiator or modified with coupling methods using dihydroxyl-terminated PEG. Currently, a number of triblock-copolymers have been prepared and characterized by these pathways, for example P(Glu(OEG_2_))-*b*-PEG-*b*-P(Glu(OEG_2_)) [91], P(Glu(OEt))-*b*-PEG-*b*-P(Glu(OEt)) [92], PLeu-*b*-PEG-*b*-PLeu and P(D,L-Leu)-*b*-PEG-*b*-P(D,L-Leu) [93], PAsp-*b*-PEG-*b*-PAsp [94], PCys(Bzl)-*b*-PEG-*b*-PCys(Bzl) [95] and PGlu(EEO_2_)-*b*-PEG-*b*-PGlu(EEO_2_) [96].

**Table 1:** Some hybrid polypeptide-containing block copolymers known from the literature.

Block Copolymers	Synthetic Pathway	*M_n_*		Ref.
		Non-Polypeptide Block	Polypeptide Block	
PEO-*b*-PTyr	Coupling	5000	6160–6980	[88]
PEG-*b*-PLys	NAM ROP by NH_2_-terminated macroI	2000	35,700	[97]
PEG-*b*-P(Lys-*co*-Phe)	NAM ROP by NH_2_-terminated macroI	2000	31,300–36,900	[97]
PEO-*b*-P(Glu/Lys-*co*-Phe)	NAM ROP by NH_2_-terminated macroI	2000/5000	24,200–4100	[98]
PEO-*b*-P(Glu/Lys-*co*-Phe)	NAM ROP by NH_2_-terminated macroI	2000	5400–8100	[98]
PCL-*b*-PEG-*b*-PGlu(OBzl)	NAM ROP by NH_2_-terminated macroI	2300	15,000	[99]
PEG-*b*-P(Lys-*co*-Leu)	NAM ROP by NH_2_-terminated macroI	2000	6500–11,000	[100]
PEG-*b*-PGlu(OBzl)	NAM ROP by NH_2_-terminated macroI	5000	7200	[101]
PEG-*b*-P(Lys-*co*-Glu(OBzl))	NAM ROP by NH_2_-terminated macroI	5000	9600	[102]
PEG-*b*-P(*γ*-propargyl-Glu)	NAM ROP by NH_2_-terminated macroI	5000/10,000	11,200–16,200	[102]
mPEG-*b*-P(D,L-Leu)	NAM ROP by NH_2_-terminated macroI	5000	5800–6400	[103]
PEG-*b*-P(Cys-*co*-Phe)	NAM ROP by NH_2_-terminated macroI	5000	7400–8200	[104]
PEG-*b*-POrn	NAM ROP by NH_2_-terminated macroI	10,000	13,500	[105]
PEG-*b*-PCys(SCbz)	NAM ROP by NH_2_-terminated macroI	5000	8500	[106]
PEG-*b*-PGlu(OBzl)	NAM ROP by NH_2_-terminated macroI	5000	7700	[106]
mPEO-*b*-PCys-*b*-PHis	NAM ROP by NH_2_-terminated macroI	10,000	15,700–16,100	[107]
PEG-*b*-PLys	NAM ROP by NH_2_-terminated macroI	5000	<17,000	[108]
mPEG-*b*-PGlu(EEO_2_)	NAM ROP by NH_2_-terminated macroI	1450–4500	3000–14,800	[96]
PCys(Bzl)-*b*-PEG-*b*-PCys(Bzl) PCys(Me)-*b*-PEG-*b*-PCys(Me)	NAM ROP by NH_2_-terminated macroI	3400; 8000 3400	5000–11,500 4500–5900	[95]
Hyaluronan-*b*-PGlu(OBzl)	Coupling of blocks	3650	8700	[89]
Dextran-*b*-PGlu(OBzl)	Coupling of blocks	6600	19,600	[90]
PMAG-*b*-P(Lys-*co*-Phe)	NAM ROP by NH_2_-terminated macroI	10,700/25,500	25,900–34,500	[109]
PMAG-*b*-PGlu(OBzl)	NAM ROP by NH_2_-terminated macroI	5300–23,300	6550–23,300	[110]
PMAG-*b*-PIle	NAM ROP by NH_2_-terminated macroI	5300–23,300	26,800–31,200	[110]
PMAG-*b*-PPhe	NAM ROP by NH_2_-terminated macroI	10,600	16,000	[111]
PNIPAAm-*b*-PGlu	NAM ROP by NH_2_-terminated macroI	7700	10,500	[112]
PE-*b*-PLys	NAM ROP by NH_2_-terminated macroI	17,000	29,500–52,500	[113]
PEOX-*b*-PGlu	NAM ROP by NH-terminated macroI	14,000	21,000	[114]

Abbreviations: NAM—normal amine mechanism; ROP—ring-opening polymerization; *M_n_*—number average molecular weight; macroI—macroinitiator. Other abbreviations are provided in Abbreviations part.

### 2.4. Dendrimers, Hyperbranched, and Star-Shaped Copolymers

**Figure 9:**
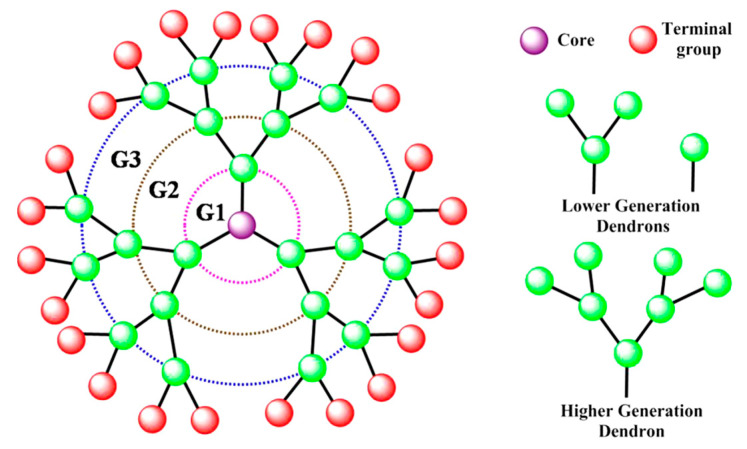
A typical anatomical structure of a dendrimer and dendrons. G1, G2, and G3 represent the first, second, and third generations, respectively. Reproduced without changes with permission from [115], Copyright© 2019, American Chemical Society.

Dendrimers are superbranched polymers characterized by a predominantly globular structure. It can be divided into three main components: the core, the inner shell (generations—G1, G2…), and the outer shell—end functional groups [115]. The generation of a dendrimer is determined by the number of branching points achieved by sequential synthesis (Figure 9).

When considering polypeptide-containing dendrimers, they can be divided into two main groups: those containing peptide cores and non-peptide cores. Among polypeptide dendrimers, lysine and glutamic acid are the most used amino acids due to the presence of the same second functional group in their structures, which directly allows the formation of branching points of AB_2_-type dendrimers. Dendrimeric polypeptides are traditionally prepared by standard techniques of peptide synthesis in solution or by the solid-phase method [116,117,118]. For example, Zhang et al. obtained Glu-based dendrimers (G4-PGlu) by peptide synthesis followed by cross-linking of two dendrimer molecules using CuAAC-catalyzed “click chemistry” to obtain the copolymer (G4-PGlu)-PEG-(G4-PGlu) [119]. The summarization of advances in the synthesis of Lys- and Orn-based dendrimers can be found in some recent reviews [120,121].

**Figure 10:**
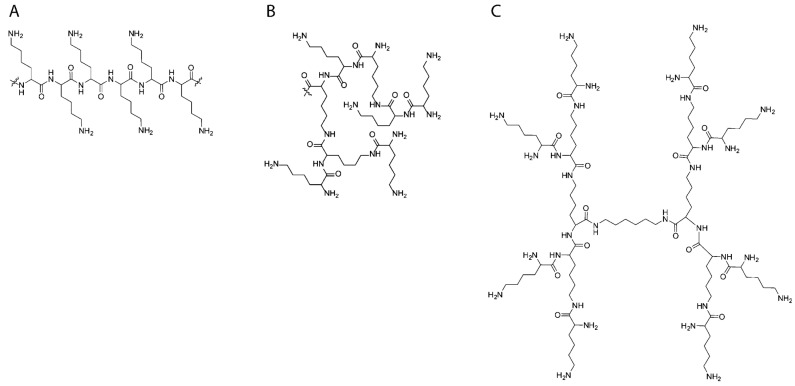
Schematic representation of the different PLys analogues: (**A**) linear PLys; (**B**) hyperbranched PLys; (**C**) third-generation dendritic PLys. Reproduced without changes with permission from [116], Copyright© 2012, American Chemical Society.

The class of hyperbranched structures is predominantly represented by Lys-based polypeptides. The main difference between hyperbranched polypeptides and dendrimers is the large defectivity of the structure and high *Đ* values [116]. Hyperbranched PLys can also be obtained by phototriggered ROP of Lys NCA [122,123] and tetrafluoroborate NCA of Lys [124], as well as catalytic thermal polymerization of Lys [125] (Figure 10).

**Figure 11:**
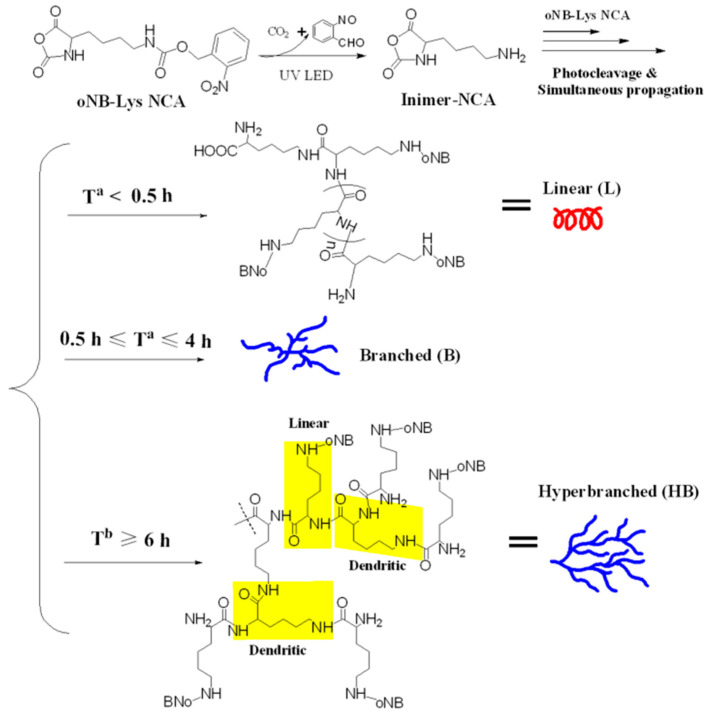
Scheme for “one pot” UV-triggered ROP of Lys(oNB) NCA at room temperature. Reproduced without changes with permission from [122], Copyright© 2017, American Chemical Society.

The main difference between such polypeptides and Lys-based dendrimers is the large defectivity of the structure and high *Đ* values [116]. For instance, Li et al. obtained photocaged NCA of Lys(*o*NB), which is able to lose its protecting group in solution under UV radiation and transform into a reactive inimer (initiator + monomer) containing a primary amino group, initiating its polymerization (Figure 11) [122]. The hyperbranched PLys were characterized by a weight average molecular weight (*M_w_*) of 1400–16,000 with *Đ* = 6.2–8.9 and a degree of branching (DB) = 0.35–0.49.

**Figure 12:**
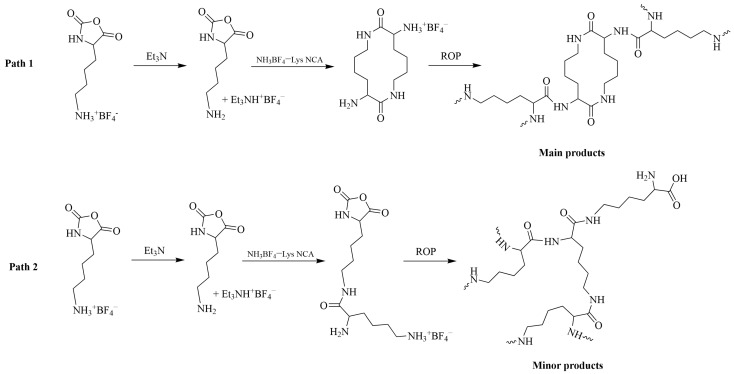
Scheme for Lys tetrafluoroborate NCA ROP catalyzed by Et_3_N at 15 °C. Reproduced with permission of Elsevier from [124].

A similar approach to obtaining Lys tetrafluoroborate NCA as an ionic monomer was proposed later in the same research group [124]. The resulting monomer was stable in DMF solutions at temperatures below 20 °C and was not prone to self-polymerization. The addition of triethylamine (Et_3_N) to the reaction system led to the formation of triethylammonium tetrafluoroborate and the inimer, which polymerized according to NAM (Figure 12). In contrast to UV-triggered ROP, the molecular weight and dispersity of hyperbranched PLys in this case were much lower: *M_w_*~2200, *Đ* = 1.1–1.3, DB = 0.51–0.54.

The method of thermal polymerization consists in keeping a mixture of Lys·HCl, alkali, and additives in the melt of the reaction system at ~150 °C for about 48 h in the presence of a catalyst, usually zirconium(IV) *n*-butoxide [125]. The use of additives is necessary for partial temporary protection of ε-NH_2_ groups, which are more reactive than α-NH_2_ groups. Applying a similar approach but using hydrophobic amino acids as additives, Liu et al. obtained a number of hyperbranched copolymers of PLys, such as hyperbranched P(Lys-*co*-Tyr), P(Lys-*co*-Ala), and P(Lys-*co*-Phe), containing 3–12 mol% of hydrophobic amino acids [126].

Graft-copolymers represent one more kind of hyperbranched polypeptide. There are three main approaches in the synthesis of graft copolymers: “grafting from”, “grafting to”, and “grafting through” [127]. The “grafting from” approach consists in synthesizing polymer side chains on a polymer backbone acting as a macroinitiator. With this approach, it is possible to achieve high side chain grafting rates at the expense of low steric hindrance. However, the difficulty in analyzing the side chains separately from the backbone complicates the characterization of the resulting graft-copolymer. In the case of “grafting to”, polymeric side chains are synthesized separately from the polymeric backbone and then grafted onto it, usually using various coupling techniques. “Grafting through” involves obtaining macromolecular monomers having end groups capable of polymerization and then polymerizing them.

**Figure 13:**
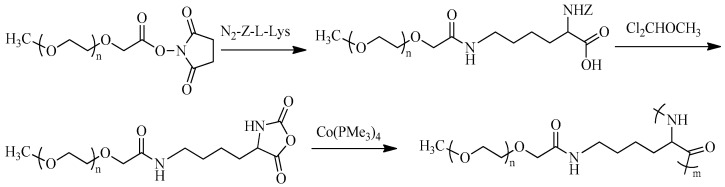
Synthesis of PLys-*g*-PEG using «grafting through» approach. Reproduced with adaptations with permission from [131], Copyright© 1999, American Chemical Society.

Graft-copolypeptides can contain a polypeptide moiety in both the main and side chains [128,129,130,131]. In this case, the main chain contains Glu, Asp, Cys, and Lys residues, which allows the polymer chain to be easily modified by a variety of conjugation techniques. As in the case of block copolymers, one of the most common types of graft copolymers with a polypeptide main chain are PEGylated copolymers. Such graft copolymers can be obtained using “grafting through” and “grafting onto” approaches. Yu et al. obtained PEGylated Lys, after which they synthesized the corresponding NCA and carried out its polymerization initiated by the cobalt organometallic complex (Figure 13) [131]. This approach also allowed the preparation of hybrid graft-block copolymers containing non-functionalized Lys and Glu units with *Đ* ˂ 1.2: PLys-*g*-PEG-*b*-PLys and PLys-*g*-PEG-*b*-PGlu.

Lee et al. synthesized a similar PLys-*g*-PEG copolymer using a “grafting to” approach [132]. PEGylation was carried out by reacting the N-hydroxysuccinimide ester (NHS) of mPEG propionic acid with a preliminary synthesized PLys backbone. The resulting graft-copolymer was characterized by a grafting ratio of (Lys units)/(PEG side chains) of 2.9.

The “grafting to” approach has also found application in the synthesis of PGlu-*g*-PEG using carbodiimide chemistry [133,134], CuAAC “click chemistry” [135,136], thiol-ene [137], and transesterification reactions [138]. In particular, Ding et al. synthesized PGlu-*g*-poly(2-(2-methoxyethoxy)ethyl methacrylate) by atom transfer radical polymerization of 2-(2-methoxyethoxy)ethyl methacrylate using PGlu(OEtCl) as a macroinitiator [139]. Anas et al. reported the use of the CuAAC “click” reaction between P(Cys-S-Propargyl) and azide-terminated functional PIPOX to obtain water-soluble amphiphilic PCys-*g*-PIPOX [140].

Graft copolymers with polypeptide side chains are represented by a wide range of compounds. In general, “grafting to” approaches to obtain such copolymers do not differ much from those described above. However, it is possible to apply the “grafting from” approach, in which the NCA of an amino acid is polymerized on a polyfunctional macroinitiator having a linear or branched structure. Polysaccharides, in particular chitin or CS, are often used as linear macroinitiators, which subsequently act as polymeric backbones. A large number of graft copolymers have been obtained in this way, for example, CS-*g*-PLys [141], CS-*g*-PGlu [142], CS-*g*-P(Phe(DH)-*co*-Cys) and CS-*g*-P(Phe(DH)-*co*-Cys-*co*-Arg) [143], and chitin-*g*-PGlu [144]. In turn, synthetic PAMAM [145] and PEI [146,147] can also be used as branching non-polypeptide macroinitiators to graft polypeptides.

Star-shaped polypeptides are a type of branched structure in which lateral linear polypeptide chains (arms) are linked to a central core. As in the case of graft-copolypeptides, there are two main approaches to the synthesis of star-shaped polypeptides: “core-first” and “arm-first” approaches, essentially similar to “grafting from” and “grafting to”. They employ similar synthetic schemes of NCA polymerization using polyfunctional cores as initiators, as well as the coupling techniques described above. Examples of polyfunctional cores for the synthesis of star-shaped polypeptides include dendritic PAMAM [145], hyperbranched PEI [146,147], polyols (pentaerythritol, dipentaerythritol, tris(hydroxymethyl)propane) [86,148,149,150], 2,2-bis-(hydroxymethyl)propionic acid (bis-MPA)-based dendrimers [151], cyclotriphosphazene-based compounds [152], and many others. Recent advances in the field of star-shaped polypeptides are discussed in detail in some recent reviews [33,46,147].

## 3. Polypeptide-Based Nanoparticles

A standard search for the term “nanoparticles” results in their definition as particles with a diameter ranging from 10 to 100 nm. Historically, the concept of “nanotechnology” was introduced by Norio Taniguchi in 1974 and developed by Eric Drexler in the early 1980s [153]. This definition was first introduced for inorganic NPs, where the transition from individual atoms/molecules to clusters/associates/supramolecular structures was accompanied by a change in the physical properties of the system. In contrast to inorganic systems, many macromolecules, even in individual form, are in fact NPs with hydrodynamic diameters (*D_H_*) ranging from a few nm to tens of nm (for example, the immunoglobulin G (IgG) has a *D_H_* of around 11 nm). In turn, the formation of NPs from a set of individual macromolecules, leading to changes in the physical and biological properties of the system, goes beyond 100 nm for some polymer systems. To date, two types of size classification can be distinguished for polymer particles. According to some sources, polymer NPs are systems up to 500 nm in diameter [154], while particles between 500 and 1000 nm are classified as submicron particles. According to other sources, polymer NPs cover the entire nanoscale range (up to 1000 nm) [155,156,157].

### 3.1. Diversity of Polypeptide-Based Nanoparticle Morphology

**Figure 14:**
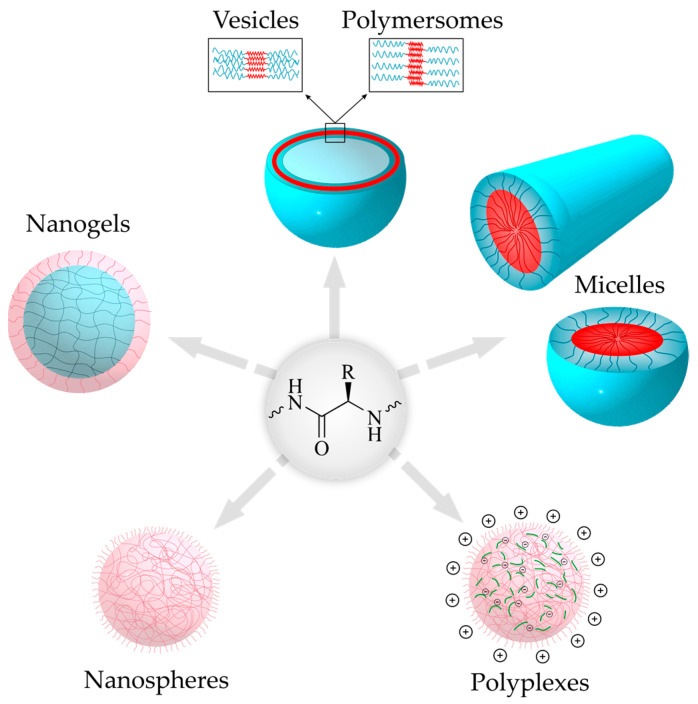
Various types of nanoparticles that can be obtained from polypeptides and polypeptide-containing copolymers.

The formation of polypeptide NPs can be controlled by physical and chemical parameters [158]. Depending on the chemical composition of the polypeptide, its molecular weight, and its hydrophobic-hydrophilic ratio, NPs of various morphologies can be formed: micelles, polymersomes, vesicles (nanocapsules), nanogels, and nanospheres [158,159,160] (Figure 14). The morphology and size of the resulting polypeptide-based NPs are also significantly influenced by the β-layers and α-helixes formed by polypeptides [161,162,163]. This organization is induced by intra- and/or intermolecular interactions due to possible hydrogen bonds, electrostatic interactions, and hydrophobic interactions between the side functional groups of the amino acids in polypeptides [20]. Varying the amino acids in a polypeptide can favor the formation of secondary conformations, while changing conditions (pH, ionic strength, temperature, etc.) can affect their disassembly to form random polypeptide coils. More details on the fundamental aspects of the secondary structure of polypeptides and its influence on the properties of polypeptides can be found elsewhere [20,21,164]

Amphiphilic copolymers (containing hydrophilic and hydrophobic domains) are capable of forming nanoscale structures (nanoparticles) by self-assembly. In the case of polypeptides, these can be synthetic copolymers of amino acids (random and block copolymers), homopolypeptides conjugated with polymer(s) of another nature into block- or graft copolymers, or homopolypeptides modified in their side chains with various moieties [51,71,165,166]. NPs can also be prepared from non-amphiphilic polypeptides *via* non-covalent modification with two polymers of opposite charges or due to hydrophobic interactions or hydrogen bonds between homopolypeptides and their copolymers [165,167,168].

#### 3.1.1. Micelles

Spherical micelles are formed from amphiphilic block copolymers by self-assembly and are characterized by a “core-shell” structure. In aqueous media, the amphiphilic block copolymers are oriented in such a way that the hydrophobic block forms a “core”, which is surrounded by a “shell” or “corona” of the hydrophilic block. Such self-assembly occurs due to the insolubility of the hydrophobic block of the block-polypeptides or hybrid block copolymers in water and, as a consequence, the tendency to minimize the contact of the hydrophobic fragment with a thermodynamically poor solvent (water). In turn, an increase in the concentration of amphiphilic copolymers leads to an increase in the free energy of the system, resulting in a structuring of the macromolecules accompanied by a decrease in entropy [161,162]. Thus, micelle formation occurs spontaneously when the system reaches the so-called “critical micelle concentration” (CMC). CMC is a specific characteristic of a certain amphiphilic system, which depends on the nature of hydrophobic units, polymer molecular weight, and the molar content of the hydrophobic fragment [158]. Above CMC, the micelles are thermodynamically stable, whereas when the system is diluted to a concentration below CMC, the micelles disintegrate [160].

In contrast to the micelles forming from small amphiphilic molecules, polymer micelles have a CMC that is one-two orders of magnitude lower. For instance, many reported amphiphilic polypeptides have a CMC of 10^−6^ M, whereas the CMC for small amphiphilic surfactants is 10^−3^–10^−4^ M [82,169,170]. This explains the greater stability of polymeric micelles in aqueous media.

The examples of polypeptide-based micelles and their characteristics are summarized in Table 2. Depending on the composition and characteristics of the amphiphilic copolymer, the latter can self-assemble into micelles with hydrodynamic diameters ranging from a few tens to several hundred nanometers and polydispersity indices (PDI) up to 0.3 (Table 2).

Besides spherical micelles, amphiphilic block copolymers can self-assemble into cylindrical or worm-like micelles [180]. In contrast to spherical micelles, cylindrical ones are usually formed from block copolymers having a crystallizable hydrophobic block, PCL, or poly-L-lactide, serving as a micellar core [181]. In this case, the term “crystallization-driven self-assembly” is usually used to underline the formation of micelles when crystallization is one of the factors. In addition, the formation of cylindrical micelles has been reported for amphiphilic ELP [182] containing non-elastin assembly domains or complementary leucine zipper motifs, respectively [183,184]. Zhang et al. observed the formation of cylindrical micelles in water from palmitoylated-polypeptide triblock amphiphiles [185]. Changing pH affected the secondary structure of the polypeptides in the micelles, from random coils at pH 2 to β-sheets at pH 7–11, but the type of micelles did not change. The formation of cylindrical micelles in an aqueous solution was also found for asymmetrical block copolymers consisting of PLys and polystyrene blocks [186].

Graft copolymers are another type of copolymer that can promote the formation of cylindrical micelles [187]. For example, Le et al. found that PEG-*b*-PGlu(OBzl) copolymers formed the typical spherical micelles [188]. However, replacing a block copolymer with 20–50 wt% of PEG-*g*-PGlu(OBzl) changed the morphology of micelles to a cylindrical shape.

Under certain conditions, spherical micelles can be transformed into cylindrical ones. For example, Sun et al. reported the preparation and study of thermolysin-responsive polypeptide-containing amphiphiles [189]. The latter were polyacrylamide copolymers grafted with KLAKLAKKKLAKLAKLAK and thermolysin-responsive GPLGLAGG peptides and differed by the degree of polymerization. The copolymers self-assembled into spherical micelles, which were transformed into worm-like micelles as a result of the enzymatic cleavage of coronal peptide side chains.

Due to their structure, micelles are suitable for the encapsulation of hydrophobic drugs. The latter are captured by the hydrophobic core of the micelle during the micelle formation process [110,190]. Drug conjugation is also possible [164]. Currently, most of the systems in clinical trials or in use are micellar systems [158]. An overview of poly(amino acid)-based micelles, among other types of polymeric micelles for cancer therapy, can be found in a recent review [191].

#### 3.1.2. Polymersomes

Polymersomes are spherical vesicular NPs similar in morphology to liposomes. Specifically, polymersomes self-assemble from amphiphilic block copolymers to form a bilayer hydrophobic polymer membrane surrounding an aqueous core [192]. Both the outer and inner surfaces of the vesicle are hydrophilic. Unlike liposomes, polymersomes have a denser membrane, which provides higher nanoparticle stability and lower membrane permeability [166]. It is known that the membrane thickness of liposomes is 3–6 nm [166,193], whereas for polymersomes, this parameter can be varied from 5 to 50 nm by modulating the hydrophobic block length [194].

As for micelles, self-assembly of amphiphilic block copolymers into polymersomes in aqueous media occurs spontaneously to minimize energetically unfavorable interactions of hydrophobic moiety with water. In this case, the concentration of copolymer at which self-assembly starts is called “critical aggregation concentration” or “critical association concentration” (in both cases abbreviated as CAC) [195]. The CAC values for polymersomes self-assembled from amphiphilic polypeptide-based copolymers are close to the CMC values for self-assembling this kind of copolymer and lie in the diapason of 10^−6^ M. The examples of polypeptide-based polymersomes and their characteristics are summarized in Table 3.

Depending on the ratio between the hydrophilic and hydrophobic parts, amphiphilic block copolymers of the same nature may form cylindrical or spherical micelles, or polymersomes. The possible morphologies of the self-assembled nanostructures primarily depend on the inherent macromolecular curvature and how it affects the packing of the copolymer chains. According to the fundamental concept, the most likely morphology of self-assembly can be predicted using a dimensionless “packing parameter” (*p*) [198]:

(1) $$p=\frac{v}{al}$$

where *v* is the volume of the hydrophobic chains, *a* is the optimal area of the head group, and *l* is the length of the hydrophobic tail.

**Figure 15:**
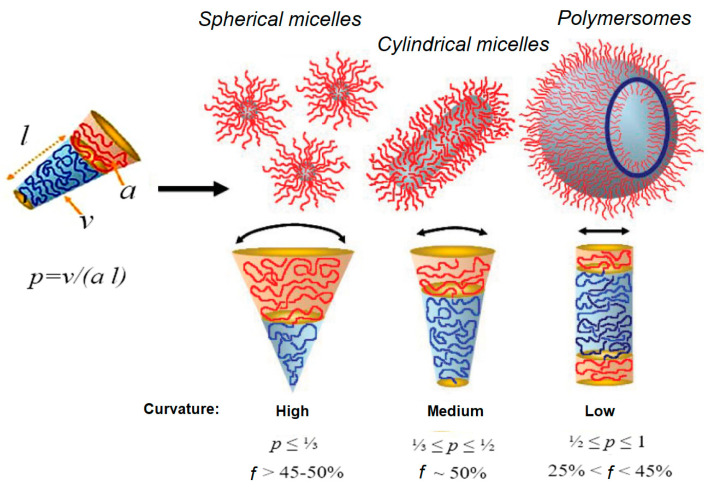
Structures formed due to self-assembly of amphiphilic block copolymers depend on composition and properties of the amphiphile. Reproduced with permission of John Wiley and Sons, Inc. from [198] and completed with information from [199].

The high curvature is favorable to the formation of spherical micelles (*p* ≤ 1/3). If curvature is medium (1/3 ≤ *p* ≤ 1/2), the cylindrical micelles are formed. In turn, when curvature is low (1/2 ≤ *p* ≤ 1), the formation of polymersomes is more likely [198] (Figure 15).

In practice, however, the hydrophilic fraction (*f*, *%*) is a more convenient parameter to predict the expected morphology [199]. As a general empirical rule, block copolymers with *f* > 50 will mostly form spherical micelles. In the region 25 ≤ *f* < 45, the formation of polymersomes is observed. Finally, the cylindrical (or worm-like) micelles are formed in a relatively narrow region: *f*~50. For instance, Checot et al. demonstrated that elongation of the hydrophilic fragment switches the morphology of self-assembled structures from polymersomes to micelles. In particular, amphiphilic block copolymers containing cationic or anionic polypeptide chains with DP close to hydrophobic polybutadiene, namely PB_48_-*b*-PGlu_56_ and PB_48_-*b*-PLys_61_, self-assemble into polymersomes. In turn, their analogs with an elongated hydrophilic polypeptide block, namely PB_48_-*b*-PGlu_114_, PB_48_-*b*-PGlu_145,_ and PB_48_-*b*-PLys_178_, formed micelles. Details on the mechanisms of polymersome self-assembly as well as the methods of shape transformation can be found elsewhere [200].

Although this empirical rule generally allows the prediction of the morphology of self-assembled structures well, the exact aggregation behavior may depend on both the nature of the block copolymer and the self-assembly conditions [166]. For example, Huang et al. have studied the self-assembly of PNIPAM-*b*-PLys(Z) depending on the length of hydrophilic and hydrophobic blocks and the organic solvent from which self-assembly was performed in water [201]. It was found that PNIPAM_197_-*b*-PLys(Z)_44_, enriched with hydrophilic fragments, formed spherical micelles from DMF and a mixture of spherical and cylindrical micelles from THF. In turn, PNIPAM_90_-*b*-PLys(Z)_71_, which has hydrophilic and hydrophobic fragments of close length, formed giant polymersomes from DMF (an organic solvent with a large dipole moment) and compact ones when they formed from THF. Ishimura et al. observed that the smaller the dipole moment of the solvent, the more the side chain of PLys(Z) is compressed [202]. Due to this, denser packing of polypeptide helices along their long axes is possible.

Yang et al. observed the formation of NPs of different morphology upon self-assembly of PGlu-*b*-PLGA at different pH values [203]. Being pH-dependent, PGlu changed its structure from an α-helix to a coil when pH was changed from 3 to 9. This led to the formation of disordered aggregates, micelles, semi-vesicles, and vesicles at pH 3, 5, 7, and 9, respectively.

In the context of drug delivery systems, polymersomes are versatile systems as they can be used to encapsulate both hydrophilic (inside a core) and hydrophobic (within a membrane) drugs [158,160,204].

#### 3.1.3. Vesicles

Vesicles are another type of hollow NP formed by the self-assembly of amphiphilic block copolymers [204]. Like polymersomes, they also have an aqueous core surrounded by a polymer shell that self-assembles due to hydrophobic interactions in water [205]. The difference between vesicles and polymersomes is that the latter, unlike the former, have a bilayer membrane. In fact, polymersomes are a type of vesicle. Sometimes researchers do not have the necessary experimental equipment to distinguish the formation of exactly polymersomes and term them in general as vesicles.

The ability of polypeptides to form supramolecular structures and the possibility of controlling this process allow the formation of various self-assembling structures. In addition to the parameters of amphiphilic polymer blocks discussed above (Section 3.1.2 and Figure 15), the conformation of polypeptide chains also influences the self-assembly process in solution and the characteristics of the nano-objects obtained. For example, copolymers containing ordered polypeptide fragments are able to form vesicles, whereas random coils form only micelles [164]. Moreover, the steric conformation of the helical parts also has a great influence on the morphology of the resulting self-assembled polypeptide structures. For example, it was shown that mixing in equimolar amounts and subsequent heating of enantiomeric nanotubes based on amphiphilic polypeptides, one with a right-handed helix hydrophobic block and the other with a left-handed helix hydrophobic block, results in the formation of a stereocomplex with a planar sheet morphology that closes into a vesicular structure [206]. It should be noted that the vesicles were formed quantitatively.

In contrast to polymersomes that self-assemble predominantly from diblock copolymers, vesicles are formed from triblock copolymers consisting of an inner hydrophobic block and two hydrophilic blocks on the sides. The hydrophilic blocks may be of the same nature or may be different, such as neutral or charged. As a result of self-assembly, one hydrophilic block forms the outer surface of the vesicle, and another one covers the inner surface of the nanoparticle. For instance, Zhen et al. reported the formation of 100–150 nm vesicles from PLys_30_-*b*-PTHF_14_-*b*-PLys_30_ modified with single glucose units. Depending on the modification, the CAC values for the copolymers were in the range of 3.0–4.6 µM. Nanovesicles of 110 and 250 nm with narrow PDI were self-assembled from PEO-*b*-PGlu(OBzl)-*b*-PLys (*M_n_* = 24,900) and PLys-*b*-PGlu(OBzl)-*b*-PLys (*M_n_* = 74,000) [207].

#### 3.1.4. Nanogels

Nanogels are a kind of soft nanomaterial formed by physical interactions or chemical crosslinking [208]. Typically, nanogels are spherical in shape, but other structures are also possible depending on fabrication methods. In addition, nanogels can be prepared as crosslinked core-shell or core-shell-corona structures [209]. Nanogels are also called nanometer-sized gel particles [210].

Physical nanogels are formed under mild conditions from amphiphilic or charged copolymers and stabilized by relatively low energy interactions (hydrophobic, ionic, or hydrogen bonds) between different (co)polymer moieties [211]. Chemical crosslinking can be provided by the formation of covalent bonds between the polymer chains (formation of Schiff-base or disulfide, click chemistry, photo-induced crosslinking, enzyme-mediated crosslinking, etc.) [211]. The recent advances in polypeptide nanogels are discussed in some recent reviews [212,213].

Some examples of polypeptide-based nanogels and their characteristics are summarized in Table 4. The main feature of nanogels is their significantly smaller (two to three times smaller) size in the dry state in contrast to their hydrodynamic diameter.

Nanogels forming due to hydrophobic interactions demonstrated the CAC values typical for the self-assembly of other amphiphilic copolymers. For instance, P(Lys-*co*-Phe) demonstrated CAC in the range of 6.7–9.4 mg/L depending on the hydrophilic/hydrophobic ratio [214]. CAC for P(Glu-*co*-Phe) with a Glu/Phe ratio of 4 was 4.2 mg/mL [214].

**Table 4:** Some examples of nanogels formed from polypeptide-based copolymers.

Copolymer	Formation	*D*_*H*, *DLS*_ (nm)	$\bar{D}$_TEM/AFM_ (nm)	PDI	Ref.
PEG-*b*-P(Glu-*co*-Cys)	Cross-linking	107	43		[215]
PEG-*b*-P(Phe-*co*-Cys)	Hydrophobic interactions + cross-linking	105–256 *^a^*	68–210		[216]
PEG-*b*-P(Phe-*co*-Cys)/DOX	Hydrophobic interactions + cross-linking	104; 146		0.31	[217,218]
PEG-*b*-P(Lys-*co*-Ala)/HA	Hydrophobic + ionic intercations	160–220 *^b^*			[219]
PEG-*b*-P(Glu-*co*-Glu(PheOMe))	Hydrophobic interactions + Ca^2+^ cross-linking	72	28	0.11	[220]
PSar-*b*-P(Phe-*co*-DCys)	Hydrophobic interactions +cross-linking	86–130 *^c^*		0.15–0.41	[221]
P(Glu-*co*-Glu(OSu))/DOX, P(Glu-*co*-Glu(OSu))-*g*-PEG/DOX	Hydrophobic interactions	142–168 *^c^*		0.17–0.25	[222]
P(Lys-*co*-Phe)	Hydrophobic interactions	110–220 *^c^*	30	0.08–0.15	[51,214]
P(Glu-*co*-Phe)	Hydrophobic interactions	90–250 *^c^*	50–121	0.16–0.27	[51,60,214]
P(Lys-*co*-Aib)	Hydrophobic interactions	185–294 *^c^*		0.18–0.24	[52]
PMAG-*b*-(PLys-*co*-Phe)	Hydrophobic interactions	170–290 *^c^*		0.22–0.36	[109]

*^a^* depending on Cys content; *^b^* depending on conditions; *^c^* depending on composition; *Abbreviations:* DLS—dynamic light scattering; $\bar{D}$—diameter; TEM—transmission electron microscopy; AFM—atomic force microscopy; HA—hyaluronic acid. Other abbreviations are provided in the list of abbreviations and in the footer to Table 2.

The formation of disulfide bonds between Cys units is one of the most widely used techniques for cross-linking amphiphilic or charged polypeptides [215,216,221]. Disulfide bonds are redox-sensitive and can be cleaved in the presence of glutathione in cells, making such systems promising for intracellular drug delivery.

Nanogels self-assembled from amphiphilic copolymers can be used to load hydrophobic drugs [223,224,225], while polypeptides containing ionizable groups can capture and retain drugs due to ionic interactions [210].

#### 3.1.5. Nanospheres

Nanospheres are matrix-type solid polymer systems. In contrast to soft NPs such as self-assembled systems and cross-linked nanogels, nanospheres are characterized by the rather dense packing of polymer chains inside the nanoparticle. As a result, such systems have close dimensions both in the dry state and in the aqueous medium. Drug substances can be encapsulated in, adsorbed onto, or chemically bound to nanospheres [204].

Generally, nanospheres are formed from hydrophobic polymers or copolymers in which the hydrophobic moiety is dominant. For instance, the NPs with the hydrodynamic diameter of 120–180 nm and narrow PDI (0.08–0.18) were successfully formed by nanoprecipitation from PCL-*b*-PGlu(OBzl) and PTMC-*b*-PGlu(OBzl) hydrophobic block-copolymers [226].

Alternatively, nanospheres can be formed from copolymers that, in addition to hydrophobic fragments, also have oppositely charged units. In this case, the packing density inside the nanosphere is achieved due to both hydrophobic and ionic interactions. Recently, Osipova et al. have reported the synthesis of P(Lys-*co*-Glu-*co*-Phe/Ile) and P(Lys-*co*-Lys(His)-*co*-Glu-*co*-Phe) copolymers and the formation of nanospheres from them [66,227]. The nanospheres that formed were quite dense. For example, the P(Lys-*co*-Glu-co-Phe) nanospheres had hydrodynamic diameter and mean diameter in the dry state (TEM) of 180 and 160 nm, respectively. At the same time, they possessed a hydrophilic coating to stabilize NPs. Dzhuzha et al. have reported the preparation of nanospheres from PGlu modified with Phe/Ile/Trp and Lys/Orn, with hydrodynamic diameters ranging from 195 to 350 nm depending on the hydrophobic amino acid [71]. As in the previous case, the diameter of the nanospheres in the dry state was only 10–20% smaller than the hydrodynamic diameter.

#### 3.1.6. Polyplexes

Polyplexes are interpolyelectrolyte complexes spontaneously formed between polycation and polyanion. Depending on the nature of polyelectrolytes, homogeneous and heterogeneous polyplexes are distinguished [167]. Homogeneous polypeptide complexes are formed when both polyanion and polycation are polypeptides, e.g., PLys and PGlu [228]. Heterogeneous polyplexes can be formed when the polypeptide is mixed with a synthetic polymer or biopolymer.

The most studied kinds of heterogeneous polyplexes are polypeptide/polysaccharide [229,230,231] or cationic polypeptide/nucleic acid [232]. Depending on the polycation/polyanion ratio, the formed polyplex can be charged positively or negatively. Recently, Weber et al. have studied the formation of polyplexes of PLys with sulfonated polysaccharides (cellulose sulfate, dextran sulfate, and heparin) [229]. At PLys/polysaccharide ratios of 0.9 and 1.1, the complexes formed were spherical NPs with diameters of 60 to 100 nm. In all complexes, PLys had an α-helical conformation. An increase in the molecular weight of PLys in complex with heparin contributed to an increase in the size of the polyplexes formed at the same ratio of positive and negative units [230].

Besides the complexation of individual polycations and polyanions, it is also possible to form complexes from copolymers consisting of polycationic and polyanionic fragments. For instance, Liu et al. prepared NPs of 60–80 nm in diameter from the CS-*g*-PGlu copolymer [233]. Song et al. reported the synthesis of cationic PAsp-*g*-CS-cyclodextrin and the formation of its polyplexes with pDNA [234]. The NPs with *D_H_* ranging from 100 to 350 nm were formed depending on the copolymer/pDNA ratio.

Polyplexes are the best systems for the delivery of charged molecules, especially nucleic acids [230] and peptide/protein drugs [235].

#### 3.1.7. Dendrimers and Dendrimers-Based Nanoparticles

Along with linear polypeptides and their copolymers, peptide dendrimers are a separate type of polypeptide delivery system [165]. Dendrimers refer to hyperbranched polymers with a three-dimensional architecture [115,236]. The main building block to obtain polypeptide dendrimers is L-lysine. Lys-based dendrimers up to six generations are known to be successfully synthesized and utilized as DNA delivery systems [118,237,238].

The shape, size, and surface chemistry can be controlled by introducing other amino acids, including neutral, negatively charged, and hydrophobic ones. The introduction of oppositely charged or hydrophobic amino acids contributes to the process of co-assembly of dendrimers. Such systems are capable of forming nanoscale polyplexes, or micelles [165]. Details on the self-assembly of peptide dendrimers and dendrimer-based NPs as delivery systems can be found in the recent related reviews [236,239].

Dendrimers and dendrimers-based NPs can be used to deliver small drugs or gene therapeutics [204,238,239].

### 3.2. Methods for Preparation of Nanoparticles and Drug Nanoformulations

In general, methods for preparation of polypeptide NPs can be divided into two types: preparation of NPs from pre-synthesized polypeptides and polypeptide-containing copolymers or formation of NPs during polymerization of monomers [160,200,240]. The approach based on the use of pre-synthesized polypeptides is more popular due to the possibility of rigorous pre-purification and characterization of the (co)polymers utilized. The advantage of the second approach is that it can be realized one-pot, but the final delivery systems may have impurities of monomers, initiators, solvents, etc. The selection of the method for the preparation of NPs is determined by the composition of the polypeptide or its copolymer and the final application of NPs [241]. The morphology of the obtained nanostructures depends on many factors, but to a greater extent is determined by the parameters of the copolymer(s) used [166] (Section 3.1.2 and Figure 15). The existing methods to produce polypeptide-based NPs are summarized below.

#### 3.2.1. Nanoparticles from Pre-Synthesized Polypeptides and Polypeptide-Containing Copolymers

##### Nanoprecipitation

Nanoprecipitation is a solvent-switch process that is based on dissolving a copolymer in a “good” organic solvent or a mixture of solvents, followed by the introduction of the polymer solution into water or buffer solution under vigorous stirring [240]. Organic solvents that are miscible with water are used for nanoprecipitation. The aqueous phase does not solvate the hydrophobic component of polypeptide amphiphiles and stimulates self-assembly [242,243]. Afterwards, depending on the boiling point of the organic solvent(s), the organic phase is removed either by evaporation (boiling point low) or dialysis (boiling point high). If necessary, the resulting aqueous dispersion of NPs can be purified from very small and/or conversely, very large aggregates by centrifugation and subsequent redispersion.

NPs from various copolymers containing polypeptide fragment(s), such as block, graft, dendritic, and hybrid copolymers, can be produced by nanoprecipitation [244,245]. This technique is mainly applied to hydrophobic (co)polymers or amphiphilic copolymers containing a pronounced hydrophobic moiety and insoluble in aqueous phases. The use of amphiphilic copolymers allows the use of nanoprecipitation without surfactants [240], which are widely used for hydrophobic polymers such as PLA or PCL. Using nanoprecipitation, various structures of NPs, such as nanospheres, micelles, vesicles, and polymersomes, can be obtained [178,201].

**Figure 16:**
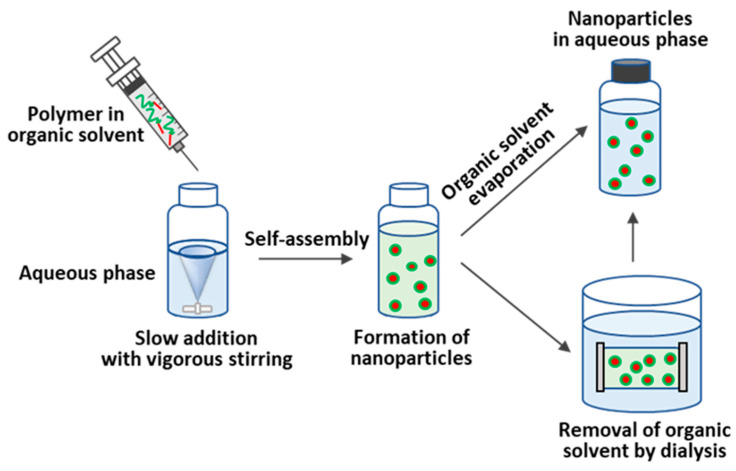
Schematic representation for preparation of nanoparticles by nanoprecipitation.

There are different variations of this method for the preparation of NPs. In the case of amphiphilic polypeptides capable of self-assembly, the aqueous phase is often added to the solution of the polypeptide or its copolymer. The addition of the aqueous phase is carried out slowly in order to avoid polymer precipitation from the solution [242,246,247]. The self-assembly process stops when the critical water content is reached, i.e., the addition of the aqueous phase does not lead to changes. Another way, widely used for more hydrophobic polypeptides or polypeptide-containing copolymers, is based on the drop-wise addition of the organic phase to water under vigorous stirring [71,72,248]. In both cases, the aqueous phase is taken in excess. The water/organic solvent ratio usually depends on the composition of polypeptide or polypeptide-containing copolymer and is optimized for each specific system. Moreover, the nanoprecipitation rate determines the hydrodynamic diameter of NPs; fast nanoprecipitation increases the nanoparticle size and polydispersity index [246]. Schematically, the essence of this method is presented in Figure 16.

The physicochemical characteristics and morphology of the NPs obtained during nanoprecipitation depend directly on the properties of the components in the system [240]. For instance, Akagi et al. tested four organic solvents, namely dimethyl sulfoxide (DMSO), DMF, dimethyl acetamide (DMAA), and *N*-methyl-2-pyrrolidone (NMP), for the preparation of NPs based on amphiphilic poly(*γ*-glutamic acid)-*g*-poly(L-phenylalanine ethyl ester) (P(*γ*Glu)-*g*-PPhe(OEt)) by nanoprecipitation and subsequent dialysis [249]. It was found that the solvent nature had almost no effect on the diameter of the resulting spherical NPs and their surface electrokinetic potential (ζ-potential). At the same time, the use of NMP (the most viscous solvent in the tested row) led to a significant increase in the NP size distribution.

Nanoprecipitation is a method suitable for the one-step preparation of drug-encapsulated NPs. For this purpose, the copolymer and the drug are dissolved in an organic solvent and then precipitated into the aqueous phase. During the nanoprecipitation process, the dissolved drug is incorporated into the hydrophobic part of the formed NPs. In this case, dialysis is usually used to remove the unencapsulated drug [10,160,240].

In general, this method is suitable for the encapsulation of hydrophobic/amphiphilic drugs but requires optimization of the polymer/solvent/water/drug system used to successfully produce drug-loaded NPs. The amount of drug added to the system also affects the final characteristics of the resulting nanoformulation. For PTMC-*b*-PGlu, the efficiency of drug (DOX) encapsulation, vesicle size, and polydispersity index of the delivery systems increased with the growth of the drug/copolymer mass ratio (from 0 to 70%) [250]. In addition, a significant influence of the pH of the aqueous phase (above and below pK_a_ of doxorubicin) on the encapsulation of doxorubicin was observed during nanoprecipitation.

The stereochemistry of the amino acids that compose polypeptides also affects the efficacy of drug encapsulation by nanoprecipitation. For example, PLeu-*b*-PEG-*b*-PLeu containing Leu-units formed DOX-loaded micelles of larger size and lower DOX loading than the same triblock-copolymer but containing racemic Leu. Moreover, copolymers containing Leu-based fragments demonstrated higher CMC due to the α-helical secondary conformation of the PLeu block [93].

##### Gradient Phase Inversion (Dialysis)

One of the variants of slow nanoprecipitation is the method of gradient phase inversion, which is most conveniently realized in the format of dialysis [200,240]. As in the previous case, this method is mainly suitable for amphiphilic block-copolymers and random polypeptides, which are better solubilized in the organic phase than in the aqueous phase. However, this method is not suitable for the formation of NPs based on highly hydrophobic copolymers. For the latter, nanoprecipitation would be a more preferable method.

**Figure 17:**
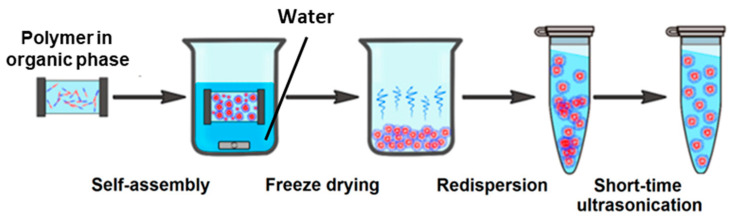
Scheme for producing nanoparticles by phase inversion method (dialysis) on the example of formation of polymersomes. Self-assembled NPs of other morphologies, e.g., micelles, vesicles, or nanogels, can also be obtained by this method. Figure reproduced with adaptation from [252] under the terms of the Creative Commons CC BY license.

In the case of gradient phase inversion, the polymer solution in an organic solvent is placed in a membrane bag, and dialysis against the aqueous phase is carried out. This provides simultaneous slow equilibration and removal of the organic solvent shifted by periodic refreshing of the aqueous phase. Dialysis is often followed by freeze-drying of NPs for storage and further redispersion in the medium of interest [51,110]. This process is schematically presented in Figure 17. Overall, the gradient phase inversion is suitable for the preparation of various types of NPs, such as polymersomes, micelles, and nanospheres [51,52,110]. This method can be successfully used for one-pot copolymer self-assembly and drug loading. For instance, Xing et al. used the dialysis method to load DOX into PEG-polypeptide nanogels modified with a near-infrared fluorescence moiety [251].

##### Direct Dissolution

The method of direct dissolution is based on the self-assembly of a polypeptide-based amphiphilic copolymer directly in an aqueous medium under a polymer concentration above its critical association concentration, followed by equilibration of the system [242,253]. The advantage of this method is that no organic solvents are needed. However, the method is suitable for amphiphilic polypeptides and polypeptide-containing copolymers with relatively low hydrophobic part content and, as a consequence, are better soluble in water. Direct dissolution mostly results in the formation of micelles, polymersomes, vesicles, or nanogels [196,242,254,255].

Ding et al. compared the influence of the methods of direct dissolution and nanoprecipitation on the size of the micelles formed [242]. It was found that micelles based on PEG-*S*-*S*-PLys(Z) and obtained by nanoprecipitation were characterized by lower dispersity and size than micelles obtained by the direct dissolution method.

This method can also be used to obtain encapsulated drug delivery systems using the one-pot technique [256,257,258]. In this case, to provide simultaneous self-assembly of the amphiphilic polypeptide/polypeptide-based copolymer and drug loading, the drug must be hydrophilic or amphiphilic to be soluble in water. Drug loading in such systems occurs through ionic interactions or hydrogen bonding with the polypeptide. In the case of amphiphilic drugs, hydrophobic interactions may also be responsible for drug encapsulation.

##### Rehydration of Films

**Figure 18:**
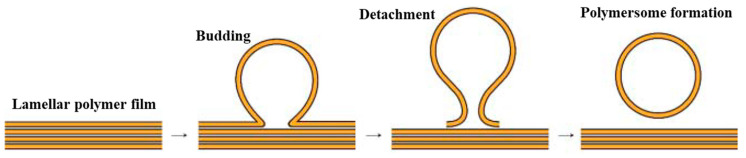
Schematic representation of polypeptide polymersome production by film rehydration. Reproduced as a part of original figure with permission of John Wiley and Sons, Inc. from [200].

Rehydration of films is initially developed to obtain liposomes and then transferred for the preparation of polymersomes or polymer vesicles [259,260]. For this purpose, the copolymer is dissolved in the organic phase, which then evaporates to dryness, accompanied by a homogeneous bulk polymer film on a substrate. This can be done under vacuum or in a nitrogen flow. Rehydration of the resulting film is carried out by adding a good solvent for the hydrophilic fragment of the polypeptide-containing copolymer (water or aqueous buffer solution) under careful stirring or gentle sonication. As a result, the formation of self-assembled NPs occurs (Figure 18) [200,261].

This technique is suitable for amphiphilic copolymers with a pronounced hydrophobic moiety, making them better soluble in organic solvents than in water. Usually, glass or Teflon are used as substrates, but gel-assisted rehydration is also possible. In the latter case, dehydrated agarose gel is used as a substrate to prepare the polymer film, and then conventional rehydration with the addition of an aqueous phase is performed. In addition, electroforming and template rehydration can be applied as external stimuli to promote rehydration. Electroforming involves the formation of a polymer film on the electrode surface, followed by rehydration in an aqueous medium under alternating current and/or voltage [262,263]. Template rehydration is based on photolithography and produces size-distribution-controlled but micrometer-sized vesicles [264].

The main advantage of film rehydration is that it allows the loading of both hydrophobic and water-soluble drugs. The drug can be added either in the organic solvent at the step of film formation or in the aqueous phase during its rehydration [200,265]. For example, photo- and redox-responsive vesicles based on PCys(S*o*NB)-*b*-PEO with an encapsulated hydrophobic anticancer drug (DOX) were produced by Liu et al. [260]. The obtained nanomedicine was characterized by a size of 83 nm and a loading capacity equal to 7.7 wt%. It was shown that DOX can be released in a controlled or on-off mode under UV irradiation or a combination of stimuli providing appropriate cytotoxicity.

The disadvantages of this method are the formation of multilayer films and, as a consequence, poor reproducibility and high polydispersity of the obtained NPs. The use of ultrasound, membrane filters, and freeze/thaw procedures allows for the minimization of these drawbacks [200].

##### Emulsification Methods

Besides the methods described above, NPs from polypeptide-based copolymers can be obtained by single or double emulsion methods, as well as emulsion-phase transfer (also called emulsion transfer) [200,266]. This method is suitable for polypeptides that are soluble in organic or aqueous phases. Depending on the inherent solubility of the copolymer, the formation of emulsion droplets occurs when the copolymer dissolved in oil/water is mixed with immiscible water/oil, respectively. Emulsification is carried out under vigorous stirring or ultrasonic treatment.

**Figure 19:**
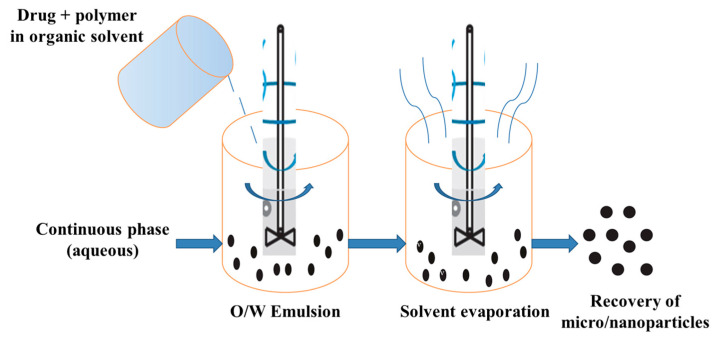
Scheme for production of nanoparticles by single emulsion method. Reproduced from [269] under the terms of the Creative Commons CC BY license.

The single emulsion method is based on dispersing the copolymer solution in an aqueous medium or dispersing the aqueous phase in the copolymer solution to form oil-in-water (o/w) or water-in-oil (w/o) emulsions, respectively (Figure 19) [240,267]. To obtain polypeptide NPs by the double emulsion method, a single o/w emulsion is emulsified in an aqueous phase to form a “water/oil/water” (w/o/w) emulsion [268]. Most often, these methods use volatile organic solvents that can be removed from the formed nano/microdroplets by evaporation (emulsification-evaporation) [240]. There are also other methods for removing polymer solvents, which are described in detail elsewhere [240].

**Figure 20:**
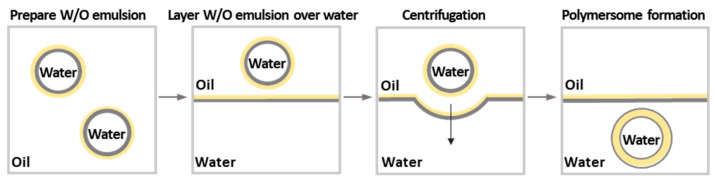
Emulsion phase transfer technique to prepare nanoparticles (given example for the formation of polymersomes). Reproduced as a part of original figure with permission of John Wiley and Sons, Inc. from [200].

In the case of the emulsion phase transfer method, a single w/o emulsion is placed in the oil phase of the prepared two-phase oil/water medium and left for some time (often several minutes) to diffuse excess copolymer at the interface and form a monolayer there. Then, the droplets are transferred, e.g., by centrifugation, from the upper (oil) phase to the lower (water) phase. After this process, so-called “phase transfer”, the oil phase is removed, and vesicles or polymersomes are obtained as w/o/w droplets (Figure 20) [200,266].

During emulsification, the orientation of the amphiphilic copolymer segments according to the environment takes place, due to which the emulsion droplets can be stabilized. Nevertheless, surfactants can be added to the aqueous phases for additional stabilization of single and double emulsions. In turn, when particles are produced by emulsion phase transfer, density-increasing and osmotic balance-supporting additives (glucose, sucrose) are added to the aqueous phases [160,200].

The final physicochemical characteristics of the resulting particles can be influenced by a number of experimental parameters, such as temperature, dispersion conditions, volume of the aqueous phase, surfactant and polymer concentration, solvent used, polymer molecular weight, method of solvent removal, etc. All these parameters can contribute to the change in the size of the droplet formed in the emulsion, which is a fundamental parameter that determines the size and polydispersity of particles [240]. Accordingly, by controlling the droplet size, it is possible to adjust the size and control the polydispersity of the particles. A powerful and promising method such as microfluidics allows to control the droplet size and reproducibly create particles [200,240]. Overall, emulsification methods allow the production of both nano-sized polypeptide particles (single emulsion) [267,270] and giant micron-sized vesicles (emulsion phase transfer) [266,271]. In terms of morphology, these can be polypeptide vesicles [266,272], micelles [270], and nanogels [267].

Among the disadvantages of emulsion methods are the possible residual impurities of the organic solvent [266], the use of additives [160], and the polydispersity of the obtained particles [200]. It should be noted that the emulsion phase transfer method allows the creation of asymmetric polymersomes. The advantage is the ease of encapsulating drugs by adding them to phases where they are soluble. Drugs of different natures can be encapsulated into polypeptide-based NPs using emulsion methods [200,250,270]. Similar to nanoprecipitation, increasing the drug’s hydrophobicity improves its loading into amphiphilic polypeptide-based NPs. For instance, the addition of triethylamine to the system containing DOX·HCl and/or changing the pH of the aqueous phase allows the regulation of DOX loading into PEG-*b*-PAsp(OBzl) (w/o emulsion method) and PTMC-*b*-PGlu (nanoprecipitation) NPs [250,270].

##### Electrospraying

**Figure 21:**
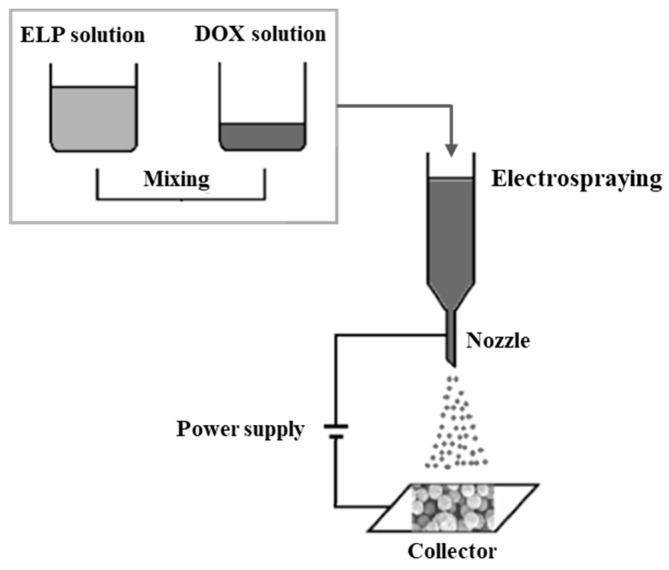
Schematic representation of the production of nanoparticles by electrospraying. Reproduced and adapted with permission from [273], Copyright© 2009, American Chemical Society.

As with many other types of polymers, polypeptides and their copolymers can be used to produce NPs by electrospraying [273,274,275]. This method is based on the electrospraying of the polymer solution in an organic solvent, accompanied by the removal of the solvent by evaporation (Figure 21). The drug-loaded nanoformulations are prepared by electrospraying the solution containing both copolymers and drugs. The technical arrangements are a limitation for the wide application of this technique. To date, the limitation for the wide application of this technique is the availability of the appropriate equipment.

Expectedly, the formation of polypeptide particles by electrospraying allows the variation of their physicochemical characteristics. Thus, Wu et al. reported the preparation of the 300–400 nm ELP-based NPs loaded with DOX [273]. It was found that the morphology, size, and polydispersity of NPs depended on the polymer molecular weight, applied voltage, and amount of organic solvent. At the same time, loading of DOX at 20 *w*/*w*% had no effect on the morphology of the obtained NPs, and drug release was consistent with the pH-determined solubility of ELP. Shao et al. varied the concentration of polymer solutions to prepare poly(γ-stearyl-L-glutamate)-based NPs by electrospraying [274]. Size and morphology of the obtained particles changed significantly depending on polymer concentration: from cup-like particles of 1 µm at 2 wt% to collapsed surface beads up to 12 µm and microfibers of ~5 µm in diameter at 16 and 22 wt%.

##### Complexation

**Figure 22:**
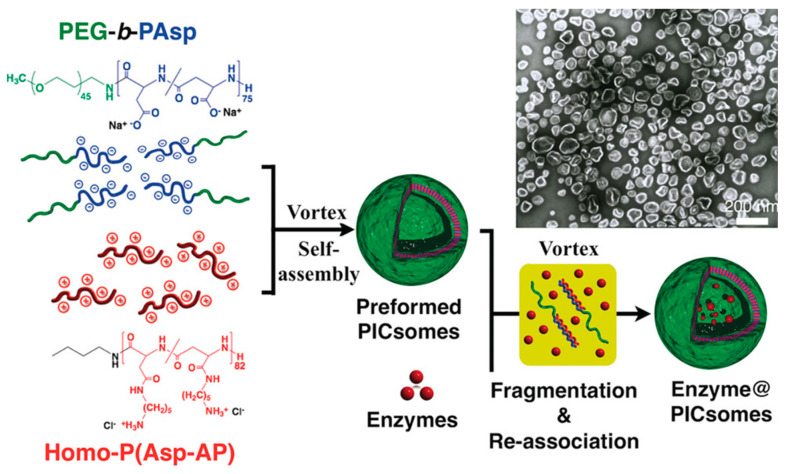
Scheme for the preparation of the enzyme-loaded polypeptide-based PICsomes. Reproduced with permission of John Wiley & Sons, Inc. from [283].

Nanoparticles can be formed due to polyelectrolyte interactions between oppositely charged polymers [164,276,277]. In this case, the resulting NPs represent interpolyelectrolyte complexes (IPECs) or polyplexes. In contrast to previous methods, which are mainly suitable for polypeptides containing a hydrophobic fragment and soluble in the organic phase, complexation is suitable for water-soluble polypeptides. The preparation of IPECs involves mixing aqueous solutions of the cationic and anionic components [278,279,280]. Depending on the polymer structure, this technique allows the production of nanogels [267,280], micelles [94,281], and vesicular assemblies called in the literature as PICsomes [164]. A schematic representation of the polypeptide-based PICsome is shown in Figure 22. Typically, a PEG-*b*-polypeptide ionomer and charged drug, a PEG-*b*-polypeptide ionomer and homoionomer, or two PEG-*b*-polypeptide ionomers are used to obtain such systems [164,282].

Complexation provides ease and efficient encapsulation of water-soluble biopharmaceuticals such as proteins, enzymes, and nucleic acids due to electrostatic interactions combined sometimes with hydrogen bonding [283,284,285]. In addition, the absence of organic solvents makes the loading of biological macromolecules safe and preserves their activity.

In addition, complexation can be used for the loading of the metal-containing drugs with the corresponding functional groups of the polypeptide [286,287,288]. For example, coincubation of PEG-*b*/*g*-PGlu and cisplatin or DACHPtCl(NO_3_) (DACHPt is (1,2-diaminocyclohexane)platinum(II)) in an aqueous medium leads to the formation of drug-loaded NPs by cross-linking the carboxyl groups of PGlu with cisplatin or DACHPtCl(NO_3_). Removal of the free drug after encapsulation can be accomplished either by dialysis [287] or ultrafiltration [289].

#### 3.2.2. Formation of Polypeptide Nanoparticles during Polymerization

##### Self-Assembly Induced by Polymerization

**Figure 23:**
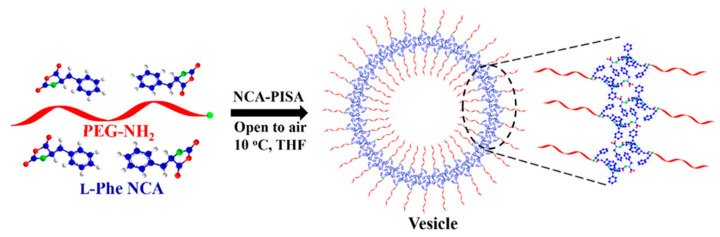
Production of polypeptide-containing vesicles by ring-opening polymerization-induced self-assembly (ROPISA) method. Reproduced with permission from [293], Copyright© 2019, American Chemical Society.

Besides traditional approaches for the production of polypeptide-based NPs from pre-synthesized and purified polypeptides and their copolymers with other polymers, the polymerization-induced self-assembly (PISA) method has recently been developed [163,290]. This method involves polymerization using a soluble polymer macroinitiator (solvophilic block) and monomers that are insoluble in their polymer form (solvophobic block). During polymerization, as the non-solvate block is synthesized, an amphiphilic block copolymer is formed and spontaneously self-assembles (Figure 23) [163,291]. PISA can be carried out under dispersive and emulsion conditions using both immiscible and solvent-miscible monomers [163]. In contrast to multi-step methods for the preparation of NPs using pre-synthesized copolymers, PISA allows one-pot polymerization and reproducible production of block-copolymer-based NPs. PISA involving ROP of NCA of α-amino acids is sometimes termed ring-opening polymerization-induced self-assembly (ROPISA) [163,292]. Production of polypeptide-containing NPs by ROPISA can be obtained in both organic [293] and aqueous media, despite the sensitivity of NCA to water [292,294].

The resulting nanostructures can have various morphologies: spherical micelles, cylindrical (worm-like) micelles, plates, vesicles, and polymersomes. The morphology of NPs can be controlled by the length of the polymer and the conditions of synthesis [293]. For instance, Jinag et al. reported the preparation of PEG-*b*-PPhe-based NPs in THF in the open air. The authors showed that increasing the molar ratio of the monomer/initiator in the reaction medium resulted in a change in the morphology of NPs from spherical core-shell NPs to single-layer vesicles. Grazon et al. prepared PEG-*b*-PGlu(OBzl) and PEG-*b*-PLys(Boc) based NPs in an aqueous medium [292]. It was noted that increasing the length of the hydrophobic block promotes a change in the morphology of micelles from elongated, needle-shaped to worm-shaped. The authors also noted a minimal number of spherical NPs obtained regardless of the length of the hydrophobic block.

##### Miniemulsion Polymerization

**Figure 24:**
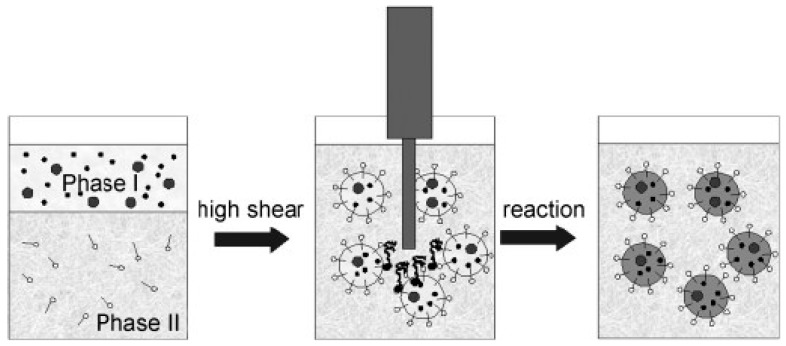
Scheme for miniemulsion polymerization. Reproduced with permission of John Wiley and Sons, Inc. from [299].

Emulsion polymerization enables the rapid and reproducible production of NPs [295]. Traditionally, emulsion polymerization is performed using monomers, initiator and surfactants/emulsifiers/co-stabilizers in droplets of solvent emulsified in the aqueous phase (o/w system) and is not suitable when the polymerization is water-sensitive, such as in the ROP of the NCA of α-amino acids [159,295]. In this case, miniemulsion (also called nanoemulsion [296,297]) polymerization can be used for the synthesis of polypeptides and the one-pot production of NPs. This method is based on the use of self-assembling amphiphilic surfactants in the reaction mixture that can protect the monomers from water by forming a shell called “a microreactor”, within which the reaction takes place [159] (Figure 24). Subsequent removal of organic solvents and impurities can be accomplished by evaporation and/or dialysis. At the end of the process, the structure formed from the surfactant remains non-covalently bound to the core portion of the resulting NPs [159,295].

It has been shown that glycosylated amphiphilic block copolypeptides can be successfully used as macromolecular surfactants in the miniemulsion polymerization of styrene and NCAs of α-amino acids to obtain narrowly distributed polypeptide-based NPs containing polypeptides or other polymers as a core [159,295,298]. The variation of the emulsification conditions allows for an influence on the physicochemical characteristics of the resulting NPs. For example, the initiator determines the mechanism and kinetics of the reaction and, in turn, may affect the size distribution of NPs. In addition, other factors also have an impact. Thus, a miniemulsion polymerization of NCA of Cys(S*o*NB) was followed by chain *S*-*S*-crosslinking of the synthesized homopolymer to stabilize the forming polypeptide NPs [295]. It was observed that the cross-linking resulted in a decrease in the size of the NPs. Examination of the morphology of these NPs by cryo-TEM showed the presence of domains of different densities, which is probably related to the formation of the secondary structure of the homopolypeptide, which is prone to the formation of exclusively β-sheet conformations. Judge et al. investigated the influence of co-surfactant and core polypeptide compatibility on the size of the obtained NPs [159]. It was hypothesized that in cases of similar nature and with the possibility of forming similar secondary structures in the core and surfactant blocks, the latter would integrate into the core structure and lead to an increase in particle size.

It should be noted that, despite the presence of surfactant in the system, in the process of polymerization, destabilization of the emulsion and enlargement of droplets can occur due to Ostwald ripening [296,300]. An osmotic agent (co-stabilizer) can be used to prevent these processes and, as a consequence, produce more narrowly dispersed nanoparticles [297,301].

Polypeptide-based NPs produced by miniemulsion polymerization described in the literature are characterized by a size of less than 300 nm with a spherical morphology, sometimes with a spherical shape with a “jellyfish”-like morphology [159,295,298]. Drug encapsulation can be carried out at the stage of polymerization by its dissolution in the appropriate organic or aqueous phase.

##### Reactive Spray-Drying

One more method for the preparation of polypeptide-based particles is in situ ROP of NCA of α-amino acids in the process of reactive spray-drying. This method is possible in the case of a very fast polymerization reaction, which can be achieved by using an appropriate initiator. For instance, Glavas et al. developed polymerization of Glu(OBzl) NCA using 1,8-diazabicyclo[5.4.0]undec-7-ene as an initiator in the process of spray-drying. The particles obtained by this method were characterized by a spherical shape and had a diameter of ~1 μm [302].

### 3.3. Properties of Polypeptide Nanoparticles and Their Formulations

Recent advances in polymer science have allowed the synthesis of well-defined polymers, including polypeptides, that can be designed for specific purposes, such as the delivery of small molecules, peptides, proteins, and genes. An important factor in the development of polymeric delivery systems is the knowledge of how the physicochemical characteristics of the polymer system affect its stability, degradability, cell penetration, cytotoxicity, macrophage uptake, and immunogenicity.

#### 3.3.1. Colloidal Stability

Stability during storage is an important characteristic of drug formulations that determines the potential clinical applications of nanomedicines. The advantage of polypeptide-containing NPs is the absence of surfactants on their surface [240]. This is ensured by the presence of intrinsic ionizable functional groups in amino acids or by conjugation with water-soluble uncharged polymers (PEG, natural and synthetic glycopolymers) that stabilize the resulting nanoformulation. High storage stability for a number of polypeptide-based NPs of various morphologies has been shown in some recent papers [51,52,60,71,303,304,305]. Moreover, some encapsulated systems also revealed high storage stability [80,250]. For example, the negligible release of DOX in the aqueous phase from PTMC-*b*-PGlu polymersomes was observed when stored at 4 °C for at least 6 months [250].

In addition to surface properties, factors such as packing density and dilution of the system affect the stability of the NPs and their drug formulations. Packing density depends on the secondary structure of the polypeptide [306] and cross-linking and affects drug release due to the diffusion of the drugs soluble in the external medium. Dilution of the system to CMC/CAC levels can also affect the stability of the NPs, which in turn favors the release of the drug from nanoformulation [307]. For amphiphilic copolymers, the structure of the hydrophobic component and the hydrophobic-hydrophilic balance affect the CMC/CAC [308]. Increasing the lipophilicity and proportion of the hydrophobic component in the copolymer reduces the CMC/CAC and, therefore, increases the stability of the NPs.

Crosslinking is another stabilization strategy to prevent premature drug release from NPs. Commonly, trigger-sensitive cross-linking is used to form reduction-responsive or pH-responsive bonds [15,164,217,309,310]. Disulfide bond cross-linking has been utilized to produce reduction-sensitive NPs for intracellular drug delivery and release. In the case of polypeptides, this task is solved by introducing Cys units into the polypeptide chain during polymerization [309,311,312] or post-polymerization modification [310], followed by the oxidation of thiols to cross-link. Incubation of *S*-*S*-crosslinked NPs in various media, including complex biological media, showed their increased stability to aggregation and cargo retention, while the presence of glutathione in the medium served as a trigger for disintegration of NPs and cargo release [310]. Linkers forming ketal, hydrazone, or Schiff bonds are used to provide pH-sensitive cross-linking of polypeptides [313,314,315]. The kinetics of drug release alter significantly when comparing the incubation of the nanoformulation cross-linked with these linkers at pH 7.4 and 5.0. For example, Lee et al. showed that the release of DOX from ketal cross-linked PEG-*b*-PAsp-*b*-PPhe micelles was 74 times faster at pH 5.0 compared to pH 7.4 [313].

Coordination of side functional groups of the polypeptide (e.g., carboxyl groups of poly(glutamic acid)) with metal complexes (e.g., platinum-containing drugs) also provides a kind of cross-linking, which leads to stabilization of nanoformulation. Comparison of the Pt(II)-loaded micelles based on PEG-*b*-PGlu and PGlu-*g*-PEG showed that structural parameters of the copolymer affect blood stability [287,316]. In particular, a more flexible regulation of PEG density in PGlu-*g*-PEG resulted in improved tolerance of these micelles regarding PEG-*b*-PGlu.

The stability of nanoformulation can also be improved by lyophilizing the encapsulated NPs with the use of cryoprotectants. Such an approach was realized by Akagi et al. for *γ*PGLu-*g*-Phe(OEt) NPs loaded with ovalbumin [249]. The resulting nanoformulation was lyophilized in the presence of glucose as a cryoprotectant. Redispersion after freeze-drying of protein-containing NPs in water showed that their size was the same as before lyophilization. Furthermore, lyophilization of NPs with glucose addition did not affect the release of ovalbumin, in contrast to NPs lyophilized without cryoprotectant. At the same time, freeze-drying in the absence of glucose promoted a significant irreversible aggregation of redispersed NPs.

#### 3.3.2. Chemical Stability and Polypeptide Degradability

Polypeptides are biodegradable macromolecules [317,318]. They are known to be cleaved by proteolytic enzymes (peptidases and proteinases) to free amino acids, which are natural metabolites. Proteinases and peptidases are widely distributed in both intra- and extracellular body fluids [319,320]. Despite their potential degradability, the rate of hydrolysis depends on the structure, composition, and chain length of the polypeptide [317], as well as the content of certain enzymes in the biological environment as specified by the various biological compartments.

Such polypeptides as PLys, PAsp, and PGlu and their copolymers have been extensively investigated [318,321,322]. PLys and γPGlu are naturally occurring poly(amino acids) [323,324]. These polypeptides are of interest for the development of various delivery systems due to the presence of ionizable and ready-to-conjugation functional groups [325]. Characterized by good biocompatibility, PGlu and PAsp, and derivatives and copolymers are known to undergo biodegradation by lysosomal enzymes [319]. Lysosomes contain a large number of hydrolytic proteases, including the cysteine proteases cathepsins B, H, and L, which play a crucial role in the degradation of proteins and polypeptides [326]. In the case of PGlu, it has been shown that polypeptide chains with a molecular weight of 11,000 are mainly excreted through the kidneys with urine and minimally retained by other body tissues [326].

Chui et al. performed a detailed study of the lysosomal degradation of both PGlu and its derivatives and copolymers to elucidate the subcellular fate of polypeptide drug carriers [319]. The influence of the introduction of hydrophobic co-monomers and the degree of neutralization of PGlu due to amidation with 3-aminopropanol or 2-aminoethanol on the enzymatic degradation process was evaluated. Introduction of Tyr and Ala into PGlu composition and neutralization of −COOH-groups of glutamic acid increased the degradability of polypeptides. At the same time, the type of hydroxyalkylamine had no significant effect on the degradation rate.

Introducing more hydrophobic co-monomers, namely Phe or Leu, into fully neutralized PGlu resulted in their faster degradation, while the difference in the use of Phe or Leu was negligible [319]. Analysis of degradation products obtained using papain, chymotrypsin, pronase, and lysosomal enzymes showed that the action of papain was similar to that of lysosomal cathepsin B. For polypeptides containing Phe and Leu, di- to pentapeptide fragments were formed. Most fragments contained hydrophobic amino acid residues (Phe or Leu), indicating a predominant action of papain on these residues. In the case of chymotrypsin, which is characterized by a well-defined specificity to the peptide bonds of hydrophobic L-amino acids, degradation also resulted in the formation of di- and pentapeptides. In turn, pronase (a mixture of proteases with strong exo- and endocatalytic activity) cleaved polypeptides to individual amino acids.

Degradation of PGlu modified with a Gly-Leu-Gly tripeptide containing *p*-nitroaniline showed a decrease in the rate of main chain degradation with an increasing degree of modification [327]. Furthermore, the release rate of *p*-nitroaniline and its amount increased with the introduction of *p*-nitroaniline. Thus, Gly-Leu-Gly can be used as a spacer for drug conjugation and ensure their efficient release under the action of lysosomal enzymes. Direct conjugation of the anticancer antibiotic adriamycin to PGlu *via* side carboxyl groups without the use of a spacer resulted in a lower rate of main chain degradation.

Romberg et al. reported the development and investigation of liposomes decorated with PGlu(OEt)/PAsp(OEt) [320]. A degradation study was performed using cathepsin B, papain, and proteinase E. Incubation of PGlu(OEt) with papain led to the degradation of polypeptides to tri- and tetrapeptides, whereas cathepsin B and proteinase E were able to degrade polypeptides totally. In contrast, PAsp(OEt) was not degraded by any of these enzymes. This effect is not surprising since PGlu is known to degrade 13- and 17-fold more actively compared with PAsp and PDGlu in liver lysosomal extracts (pH 5.4, 48 h incubation) [328]. A study of polypeptide-decorated liposomes in rats showed that their circulation in the blood was longer than that of free liposomes and comparable to that of PEG-lysosomes. Despite the different degradation behavior in the presence of enzymes, the circulation time of PAsp(OEt)-liposomes was not markedly longer than that of PGlu(OEt)-liposomes. Although the effects of PEG and PGlu(OEt)/PAsp(OEt) were similar, the use of enzymatically degrading polypeptides has the advantage of reducing the risk of in vivo accumulation.

PLys and its derivatives/copolymers capable of forming complexes with polyanions are of great interest for gene delivery (siRNA, mRNA, pDNA, etc.) [329,330]. Ren et al. studied the effect of PLys enzymatic biodegradation on DNA release from layer-by-layer self-assembled PLys/DNA multilayer film [321]. Incubation of material in PBS (phosphate-buffered saline) at 37 °C for 35 h in the presence of α-chymotrypsin resulted in 90% near-linear release of DNA and degradation of about 85% of the film. Zashikhina et al. showed that the release of irinotecan from PLys-*b*-PLeu polymersomes without enzyme at 37 °C did not exceed 30% within a month, while in the presence of papain/pepsin, complete release of the drug was achieved within 4 days.

Shi et al. studied the non-enzymatic degradation of PGlu-*g*-PEG NPs in deionized water at 37 °C. The copolymer average molecular weight decreased from 35,200 to 19,600 in 34 days. The obtained result was attributed to the hydrolysis of the ester and amide bonds of PGlu-*g*-PEG NPs. In turn, no significant change in molecular weight was found at 4 °C during the same period [316].

The characteristic secondary structures of polypeptides and the ability of these structures to break down under the influence of some factors also impact the degradation processes of polypeptides. For example, PGlu is characterized by a conformational change from a rod-shaped α-helix to a chaotic tangle with increasing pH and therefore undergoes strong pH-dependent enzymatic degradation. The rate of PGlu degradation catalyzed by papain decreased at pH < 5 (high proportion of α-helixes) as well as at pH < 0.5 (high charge density) [326].

The influence of L/D amino acids in the polypeptide on the degradation process was studied for conjugates of PLys and PDLys with methotrexate. Both conjugates entered the cells, but unlike PLys-methotrexate, PDLys-methotrexate did not show any therapeutic effect due to its inability to be degraded by lysosomal enzymes [319]. However, partial insertion of D-α-amino acids, non-coding, or unnatural amino acids into the polypeptide chain contributes to the stabilization of the macromolecule against enzymatic degradation [51,52]. Among the non-coded amino acids that can slow down enzymatic degradation is Aib [331]. Comparison of the stability of PLys-*b*-PAib polymersomes and P(Lys-*co*-Aib) NPs with PLys-*b*-PLeu polymersomes in papain-containing solution confirmed the higher stability of Aib-containing polypeptide NPs [52].

Thus, the rate of enzymatic degradation of polypeptide systems can be adjusted, and consequently, the rate of drug release can be controlled by varying the molecular weight of the polypeptide and/or block or graft copolymers, branching, hydrophilic/hydrophobic balance, composition and sequence of monomers in the polypeptide, secondary structure and chirality of amino acids in the polypeptide, as well as modification of side functional groups, method for drug conjugation, and type of spacer.

#### 3.3.3. Cell Uptake, Cytotoxicity, and Specific Targeting

It is well known that the size, surface characteristics, and texture of polymeric NPs play a crucial role in their biological properties. There are several mechanisms responsible for the internalization of NPs inside the cells [332]. Among them are pinocytosis and clathrin- and caveolae-mediated endocytosis. Pinocytosis is based on the internalization of extracellular fluid (“cellular drinking”), in which NPs may not interact directly with the cell membrane. Pinocytosis is characteristic of practically all cells. Absorbed objects, housed in small vesicles, are called pinosomes. Pinocytosis involving the absorption of a large volume of fluid and the formation of large vesicles (0.5–10 μm) is referred to as macropinocytosis. This pathway allows microparticles that cannot be taken into cells by most other mechanisms to be delivered inside the cell [333].

In turn, clathrin- and caveolae-mediated endocytosis are based on the interaction of NPs with the cell membrane [333]. Clathrin-mediated endocytosis (CME), also known as receptor-mediated endocytosis, is based on the interaction of NP components with clathrin receptors, followed by the formation of clathrin-coated vesicles. CME is the mechanism used to internalize ligand-conjugated NPs in targeted drug delivery. Blocking clathrin receptors results in decreased cellular uptake of NPs [333]. Inside the cell, clathrin coats located outside the vesicles are displaced prior to fusion with early endosomes. CME is one of the main mechanisms for the cellular uptake of 100–350 nm anionic NPs [333]. This pathway also underlies the cellular uptake of opsonin-bound NPs [332].

The caveolae-mediated pathway involves flask-shaped invaginations of the membrane called caveolae. The latter are composed of the membrane proteins caveolin-1, sphingolipids, and cholesterol. It formed caveolae separate from the plasma membrane and fused with the cell compartment, forming caveosomes. The feature of caveosomes is their neutral pH and ability to bypass lysosomes. Features of caveosomes are their neutral pH and ability to bypass lysosomes. This way of entry into the cell allows for the protection of NPs and their cargoes from lysosomal degradation [334]. Caveolae-mediated endocytosis is the preferential pathway for entry of small (up to 100 nm) anionic and PEGylated NPs, as well as NPs with hydrophobic domains, such as those formed by cholesterol and fatty acids [332,333]. The particle size is limited by the possible size of the caveosomes formed. At the same time, the presence of the cationic lipid sphingomyelin in the composition of caveosomes is responsible for the interaction with anionic and hydrophobic objects. Caveolae are characteristic of many cell types, such as endothelial and epithelial cells, fibroblasts, and muscle cells.

Summarizing the aforesaid, it can be concluded that the size and charge of NPs play a key role in the cellular uptake pathway. However, besides this, the physicochemical characteristics of particles also determine the efficiency of cellular uptake and the cytotoxic potential of NPs. Systemic studies analyzing the effect of size on the penetration of polypeptide NPs have not been found, but numerous studies on polymer particles have shown that small NPs are internalized at a higher rate than large ones [335,336,337,338]. The reason for this is the different time and energy required to form a vesicle and to internalize a small and a large particle inside the cell. In addition, small NPs can be internalized simultaneously inside a single vesicle, which is impossible for large particles due to their size [339].

Surface charge is another factor affecting cellular uptake and cytotoxicity. Many studies for polymer NPs, including polypeptide ones, revealed higher cytotoxicity for positively charged NPs [334,340]. This is associated with the increased interaction of positively charged NPs with the negatively charged cell membrane. In turn, this leads to better internalization of cationic NPs compared to neutral and negatively charged ones. However, the uptake of positively charged NPs may significantly disintegrate the cell membrane and, as a result, cause cell death. This effect becomes more pronounced when the concentration of positively charged NPs increases [109]. Oxidative stress is among the other mechanisms of NP cytotoxicity [341]. In particular, it has been recognized that charged NPs can interact with mitochondria, affecting the electron transport chain (ETC).

Comparison of cellular uptake rates for positively charged P(Lys-*co*-Phe) (ζ-potential = 36 mV), negatively charged P(Lys-*co*-Phe)/HEP (ζ-potential = −38 mV), and neutral PMAG-*b*-P(PLys-*co*-Phe) (ζ-potential = 1.5 mV) NPs revealed a 3- to 4-fold increase in the accumulation of cationic NPs compared to anionic and neutral NPs in A549 cells [109]. At the same time, both P(Lys-*co*-Phe) and P(Lys-*co*-Phe)/HEP were efficiently captured by mouse macrophages (J774.1A), whereas the capture of PMAG-*b*-P(PLys-*co*-Phe) was significantly reduced and close to PEG-*b*-PLA NPs. In turn, negatively charged P(Glu-*co*-Phe) NPs with *D_H_*~200 nm demonstrated a 2-fold lower rate capture by macrophages than PEG-*b*-PLA NPs [60]. Low uptake of negatively charged NPs was recently shown for negatively charged self-assembled NPs based on PGlu post-modified with different amino acids and glucose [71].

Enhanced interaction and uptake of cationic NPs in cells result in increased cytotoxicity. Numerous studies show that most positively charged polypeptide NPs (enriched with Lys and/or Arg) are non-cytotoxic up to a concentration of 80 µg/mL for normal and cancer cells [66,303,342,343]. The modification of Lys-based NPs with His allowed a slightly wider range of biocompatibility. For example, Osipova et al. reported that partial modification of Lys residues in P(Lys-*co*-Glu-*co*-Phe) NPs with His residues promoted a twofold reduction in cytotoxicity. The viability of HEK 293 cells increased from 32 μg/mL for P(Lys-*co*-Glu-*co*-Phe) NPs to 64 μg/mL for P(Lys-*co*-Lys(His)-*co*-Glu-*co*-Phe) NPs while maintaining the size and zeta potential of NPs. The level of cytotoxicity also depends on the cell phenotype, composition, and packaging of polypeptides in NPs, which determine the surface localization of certain amino acids [72,210,310].

In contrast to cationic NPs, negatively charged and neutral NPs are known to have much higher cytobiocompatibility [60,71,110,190,219]. Most polypeptide-containing negatively charged or neutral NPs are non-toxic to normal and cancer cells up to concentrations of 1000 μg/mL and higher. Negatively charged polypeptide NPs contain Glu or Asp-residues [51,94,177] or a negatively charged polymer fragment of another nature, e.g., hyaluronic acid, in the copolymer structure [89]. A near-neutral surface charge is most often achieved by introducing an uncharged polymer into the copolymer as a hydrophilic fragment. In this case, PEG [171,178,312], neutral natural polysaccharides [90,210], and synthetic glycopolymers [109,110] are the most selected types of polymers. It is known that shielding polypeptide-containing NPs with PEG contributed to the reduction of blood clearance due to the fast elimination by enzymatic degradation, opsonization, and uptake by macrophages [344,345,346]. In addition, the introduction of such a fragment reduces the cytotoxicity of cationic polypeptide NPs. For example, Zashikhina et al. showed that recharging cytotoxic P(Lys-*co*-Phe) NPs by coating them with heparin dramatically changed the properties of NPs [109]. Without heparin coating, the half maximal inhibitory concentration (IC_50_) values for P(Lys-*co*-Phe) NPs in HEK 293 and BEAS-2B cells, determined after 24 and 72 h of incubation, were ≤50 µg/mL. In turn, coating these NPs with heparin promoted an increase in IC_50_ values to >1000 μg/mL for BEAS-2B cells for 24 and 72 h of incubation, and to >1000 μg/mL and 280 μg/mL for HEK 293 cells for 24 h and 72 h of incubation, respectively. A similar result was obtained for NPs when a neutral synthetic glycopolymer (PMAG) was used to covalently coat P(Lys-*co*-Phe) NPs or self-assemble NPs from pre-synthesized PMAG-*b*-P(Lys-*co*-Phe) [109].

Besides surface charge, the size of NPs also affects cytotoxicity due to better cellular uptake. For example, Iudin et al. reported a more pronounced cytotoxicity of ~100 nm PGlu@Ag (Ag—silver) NPs compared to ~500 nm hybrid NPs [347]. The IC_50_ values for small PGlu@Ag NPs tested in normal (HEK 293) and cancer (HepG2) cells were 85 and 193 μg/mL, respectively. The higher cytotoxicity of small particles is related to both the intrinsic cytotoxicity of Ag and the better cellular uptake of small NPs. Control P(Glu-*co*-Phe) NPs of 200 nm were nontoxic up to 1000 μg/mL. Moreover, an experiment on uptake of NPs by mouse macrophages (J774.1A) revealed the higher uptake efficiency for ~100 nm PGlu@Ag NPs and the lower for P(Glu-*co*-Phe) NPs of 200 nm, while the ~500 nm hybrid NPs demonstrated an intermediate result. In this case, in addition to size, stiffness affected cellular uptake. Therefore, larger but stiffer hybrid NPs were taken up faster than the smaller soft P(Glu-co-Phe). The influence of NP stiffness/elasticity on their uptake by cells has been described in many studies [348,349,350]. It has been found that stiffer NPs are internalized more efficiently than softer ones. This result is explained by the lower total energy required to envelop stiff NPs in the membrane compared to softer NPs, which are prone to change their shape, in particular, to flattening.

In addition, the rate of cellular uptake may depend on the conditions of the NPs. For example, Ding et al. observed a 3-fold enhanced internalization of PEG-*b*-PCys NPs with a disulfide-crosslinked core loaded with camptothecin into HeLa cells at pH 6.5 compared to pH 7.4 [309]. Liu et al. developed near-infrared (NIR)-sensitive NPs based on PEO-*b*-P(Cys(S*o*NB)) and containing an upconverter, allowing the conversion of NIR light into UV light [351]. This delivery system containing loaded DOX demonstrated NIR-induced internalization into cells and improved cancer cell inhibition.

Modification of NPs with specific ligands allows for enhanced receptor-mediated internalization into certain cells. In particular, cancer cells are known to overexpress folic acid receptors [352]. This feature has been widely used for targeting drug delivery systems to cancer cells [353]. Thus, modification of NPs with folic acid improves their entrance into the cell. For instance, a folate receptor-mediated endocytosis was realized to enhance the intracellular delivery of FA-modified polypeptide NPs based on PEG-*b*-PGlu vesicles [354] and PEG-*b*-PHis micelles [355] into HeLa cancer cells. Besides FA, specific peptides have been widely used as ligands to modify NPs and realize receptor-mediated internalization. To realize this strategy, Wang et al. modified PEG-*b*-PGlu-*b*-PHis-*b*-PLeu NPs with LyP-1 peptide (cyclic Cys-Gly-Asn-Lys-Arg-Thr-Arg-Gly-Cys), which has affinity for the p32 or gC1qR receptor [356]. The latter reveals aberrant expression in breast cancer cells. Comparison of unmodified and LyP-1-modified polypeptide NPs showed enhanced internalization of ligand-containing NPs into MDA-MB-435 breast cancer cells. Xue et al. all used cRGD-peptide, which is known as a ligand to *α*v*β*3 and *α*v*β*5 receptors on cancer cells, to improve the delivery of DOX-loaded polypeptide micelles into MDA-MB-435 breast cancer cells [190]. The use of targeting peptide cRGD as a ligand to modify PGlu-*g*-PEG micelles to enable selective intracellular co-delivery of docetaxel and cisplatin has also been reported [134].

#### 3.3.4. Hemolysis and Tissue Permeability

Hemocompatibility is one of the most important properties for drug delivery systems. Being foreign materials, NPs may have a potential risk of causing the destruction of red blood cells, called hemolysis [357]. For most NPs, hemolytic activity depends on the concentration, size, and chemical composition of the surface [358]. As for cellular uptake, the smallest NPs are more hemolytic active than larger ones due to better uptake by erythrocytes. At high concentrations of positively charged NPs, blood cells may aggregate because of multiple electrostatic surface interactions and non-covalent crosslinking [359]. This is also valid for cationic polypeptides such as PLys [360]. Moreover, as it has been shown for cationic liposomes, the positive surface charge can influence immunotoxicity by stimulating neutrophils and inducing oxidative bursts in these cells [359,361]. Recently, Zhu et al. studied the hemolytic activity of sulfonium cationic PMet [362]. The minimum concentration causing 10% hemolysis was found to be above 400 µg/mL. In turn, the hemolytic activity of negatively charged NPs is quite low. For instance, incubation of PGlu-*b*-PLeu polymersomes in human blood at concentrations of 2–125 μg/mL caused hemolysis only by 0.4–0.6% [363]. A similar result was recently shown by Zhang et al. for NPs formed from PGlu and γPGlu modified with Phe(OEt) [364]. Hemolysis for both types of NPs did not exceed 1% at pH 7.4.

As a negative charge, PEGylation of polypeptides also reduces their hemolytic activity [365,366]. Thus, surface properties may alter the safety profile of delivery systems for blood cells.

Overcoming biological barriers is another key property, depending on the size of the drug delivery systems. Cabral et al. compared the accumulation of sub-100 nm PEG-*b*-PGlu micelles in highly and poorly permeable tumors in tumor-derived mice [367]. The results showed the independence of permeability of 30, 50, 70, and 100 nm NPs into hypervascular tumors with a highly permeable structure. In turn, only NPs smaller than 50 nm penetrated poorly permeable hypovascular tumors. At the same time, using a transforming growth factor (TGF-β) signaling inhibitor, which reduces the pericyte coverage of the endothelium in the neovasculature of tumors, improved the accumulation and distribution of larger NPs. In this case, 70 nm PEG-*b*-PGlu micelles accumulated to a level comparable with that of the 30 nm NPs. Later, Matsumoto et al. studied the permeability of tumor blood vessels to PEG-*b*-PGlu NPs of two sizes (20 and 70 nm) loaded with DACHPt in cancer-derived mice under eruption conditions (vascular bursts followed by a brief vigorous outward flow of fluid) [368]. The authors found that 20 nm NPs entered tumor tissue *via* both static permeability and eruption, whereas permeability of 70 nm was mostly provided by eruption.

PEG-containing NPs have a prolonged circulation in the blood, which is necessary for passive targeting and accumulation in a pathological focus, e.g., in tumor tissue. In particular, Li et al. developed Ca^2+^-crosslinked PEG-*b*-PGlu DOX-loaded NPs and studied this nanoformulation in an osteosarcoma mouse model [369]. It was found that 2 h after the intravenous administration of the nanoformulation, the latter accumulated predominantly in the tumor and, to a lesser extent, in the lungs and kidneys. After 6 h, intratumoral accumulation reached a maximum. In addition, compared with free DOX, administration of the nanoformulation reduced the myocardial damage. A similar result was also observed by Yin et al. for PEG-*b*-P(S-tert-butylmercapto-Cys) loaded with DOX [311]. The low cytotoxic effect on the heart and the high intratumoral accumulation were also detected. However, in this case, the authors also revealed the noticeable accumulation of the nanoformulation in the liver. Zheng et al. studied the biodistribution of rhodamine-labeled PEG-*b*-PLys-*b*-PLeu micelles designed for co-delivery of docetaxel and siRNA in nude mice [342]. The fluorescence signal of the tag was detected in the liver and tumor, and the signal was significantly more intense in the tumor. These studies confirm that PEGylated polypeptide NPs provide effective passive targeting.

Nevertheless, the modification with PEG may slow down cellular uptake and limit therapeutic efficacy. To overcome this drawback, polypeptides with a cleavable PEG block have been designed and investigated. Recently, Jiang et al. synthesized PEG-*b*-PGlu, whose blocks were linked by a pH-sensitive (amide bond derived from 2-propionic-3-methylmaleic anhydride) or matrix metalloproteinase-2/9-sensitive peptide (ProLeuGlyLeuAlaGly) linker cleavable in the tumor environment [370]. The resulting cisplatin-loaded PEG-*b*-PGlu NPs demonstrated prolonged circulation in the blood as well as enhanced accumulation in tumor tissues and inhibition of tumor growth due to on-site PEG cleavage.

Despite the known stealth effect of PEG and PGlu, the successful administration of cationic polypeptide NPs in vivo is also reported. Yao et al. evaluated the disulfide-cross-linked PArg-*b*-PHis-stearoyl micelles designed for DOX and miRNA co-delivery [303]. The biodistribution study of the 1,1′-dioctadecyltetramethyl indotricarbocyanine iodide-loaded micelles was performed after the intravenous injection into tumor-bearing mice. The scanning of live mice 2 h after administration revealed a tumor-site localization more intensive than in the rest of the tissues. The highest signal of the dye was detected in the tumor after 12 h. At the same time, no signal of the dye in the tumor was detected when it was administered in a free form. The obtained result is related to the enhanced permeability and retention (EPR) effect of NPs and their avoidance of the reticuloendothelial system (RES) due to their small size (170 nm) and soft nature.

#### 3.3.5. Immunogenicity and Toxicity

Due to the number of positive structural features and properties of polypeptide NPs, they have great potential as drug delivery systems. Among other biological properties, the immunogenicity of polypeptide-containing NPs is one of the most important issues. Such properties of NPs, such as size, surface charge, and stiffness/elasticity affect the interaction and triggering of certain immune cells. Being similar to proteins, synthetic polypeptides can be a cause for the appearance of the immune response. Today, to improve the existing vaccines or develop novel ones, polypeptide NPs are also a matter of choice. Some polypeptide NPs are considered as adjuvants to increase the immune response [371,372], while others are used as antigen for the development of nonadjuvanted vaccines [373].

PLys NPs are known to be quite immunogenic, and this property is widely considered in vaccine development [374]. PLys-coated polystyrene NPs loaded with DNA were tested as vaccines in a mouse model [375]. In this case, PLys-coated NPs induced high levels of CD8 T cells and the production of specific antibodies. At the same time, the immunocompatibility and toxicity study of PLys nanocapsules (360 nm) showed that toxicity markers, changes in hematologic parameters, and important immunomodulatory genes had insignificant changes compared to the control [376]. Thirty days after treatment with PLys nanocapsules of different concentrations, almost normal architectonics was observed in the histopathologic examination of the major tissues.

Kim et al. studied the immunogenicity of cationic NPs of various sizes, morphologies, and elasticity based on PSar-block-copolymers [377]. A number of amphiphilic PSar-containing copolymers, namely PSar_73_-*b*-PLA_30_, PSar_21_-*b*-(Leu-Aib)_6_, PSar_27_-*b*-(DLeu-Aib)_6_, PSar_25_-*b*-(DVal-Aib)_6_, PSar_21_-*b*-(Leu-Aib)_8_, and PSar_26_-*b*-(DLeu-Aib)_8_ were synthesized by a combination of peptide synthesis and ROP of Sar NCA. Four kinds of self-assembled NPs were prepared from the copolymers obtained: (1) PSar_73_-*b*-PLA_30_ micelles of 35 nm (G1), (2) PSar_21_-*b*-(Leu-Aib)_6_ + PSar_27_-*b*-(DLeu-Aib)_6_ vesicles of 194 nm (G2), (3) PSar_21_-*b*-(Leu-Aib)_6_, PSar_27_-*b*-(DLeu-Aib)_6_ + PSar_25_-*b*-(DVal-Aib)_6_ vesicles of 229 nm (G3), and (4) PSar_21_-*b*-(Leu-Aib)_8_, PSar_21_-*b*-(DLeu-Aib)_7_ and PSar_26_-*b*-(DLeu-Aib)_8_ vesicles of 85 nm (G4). The twice-immunization of BALB/c mice with the PSar-containing self-assembled NPs of different sizes and the same surface characteristics revealed the difference in antibody production. Immunization of mice with G1 resulted in the production of the largest amount of IgM. In turn, the largest amount of IgG3 was produced after immunization with G3 or G4. At the same time, the increased avidity of IgG3 after single administration was observed for G3. G2 and G3 vesicles were similar in diameter, but G2 exhibited 3-fold greater elasticity. This resulted in different avidities for IgG3, since it is known that B cells are better activated by stiffer substrates [378]. The study of the biodistribution of these NPs also revealed the differences. After the first injection, the extent of circulation of NPs throughout the body decreased proportionally to the size of the NPs: G1 > G4 > G2~G3. Accumulation in the liver after the second injection, indicating an accelerated blood clearance (ABC) phenomenon, was most intense when G1 was administered, followed by G4 and to a lesser extent for G2 and G3. This order is consistent with the amount and avidity of IgM produced by these NPs. It means that IgM production is a more dominant factor for the induction of the ABC phenomenon than IgG3 production. Thus, the differences in the physicochemical characteristics of NPs influence the antibody switching process.

In contrast, anionic polypeptide-based delivery systems are known to possess low immunogenicity. PGlu enhances T helper cell type 1 (Th1)-mediated immune responses by promoting the activation of dendritic cells [379]. Recently, Sakhabeev et al. reported the four-step immunization of mice with fibrillogenic β2-M protein conjugated to P(Glu-*co*-Phe) NPs (*D_H_* = 130 nm) [380]. The downregulation in the production of specific antibodies (humoral immune response) was detected for such a system compared to the mixture of PEG-*b*-PLA NPs with free β2-M protein used as a control. In turn, Uto et al. found that the immunization of mice with ovalbumin conjugated to γPGlu NPs (*D_H_* = 210 nm) induced significant activation of CD8^+^ T cells [381]. Similarly, the suitability of ovalbumin-loaded NPs based on PGlu modified with Phe(OEt) (*D_H_*~250 nm) for enhancing cellular immunity has been recently reported by Zhang et al. [364].

Experimental results reflecting the patterns between surface charge and toxicity of polypeptide-based NPs in vivo have not been found. However, comparison of the results of amine-terminated and carboxyl- and hydroxyl-terminated dendrimers showed that cationic dendrimers had higher in vivo toxicity (in terms of maximum tolerated dose) [332]. This resulted in more pronounced intravascular coagulation and hemolysis. In addition, while cationic dendrimers accumulated significantly in the liver, negative and neutral dendrimers demonstrated longer circulation times. Similar results may be expected for polypeptide NPs with appropriate surface functionalities.

## 4. Polypeptide-Based Hydrogels

Hydrogels are easily swellable in water soft three-dimensional systems, which are formed by cross-linked hydrophilic or amphiphilic (co)polymers [382,383]. Hydrogels can be prepared from various natural and synthetic macromolecules, including polypeptides [164,315,384,385,386]. Polypeptide-based hydrogels are characterized by their biocompatibility and biodegradability. Depending on composition, they can possess their own biological activity and biomimetic mechanical properties, the ability to mimic some properties of extracellular matrix constituents, sensitivity to stimuli, and suitable injectability. Currently, hydrogels are of great interest as biomaterials for drug delivery, wound healing, and tissue engineering [16,27,46,385,386,387,388].

Like other hydrogels, polypeptide-based hydrogels are prepared in two possible ways: by physical gelation or chemical crosslinking. The wide variety of functionalities in the side chains of amino acids contributes to a wide range of cross-linking techniques. Physical polypeptide hydrogels can be obtained from polypeptide-based amphiphilic block copolypeptides or polypeptide-containing block copolymers, homopolypeptides with modified side chains (hydrophilic/hydrophobic component) or alkyl tail (hydrophobic component), branched polypeptides, as well as mixtures of modified and unmodified polypeptides both with each other or with other polymers [16,27,95,382] and biologically active molecules [389].

The main pathway for the direct preparation of the polypeptide-based physical gels is the self-organization of polypeptides due to the formation of secondary structures (α-helixes and β-sheets) [27,46,390]. Often, such hydrogels are stabilized by hydrophobic interactions between α-helix and β-sheet-containing building blocks [388,391]. At the same time, the gelation force increases in the order α-helix > β-sheet > random coil [392]. Polypeptides that are capable of forming β-sheet secondary structures are most responsible for producing physically cross-linked hydrogels [27,393]. There are also alternative methods for forming physically cross-linked polypeptide hydrogels based on modification of the polypeptides to provide, for example, host-guest or electrostatic interactions [16]. Varying the environmental conditions (pH, temperature, solvent, polypeptide concentration, ionic strength) and polypeptide composition (ionic/non-ionic, amino acid chirality, branching structure, macromolecule length, substituents in side chains, etc.) allows tuning the hydrogel macroscopic properties [86,382,394,395,396].

For example, Phan et al. showed that an amphiphilic triblock copolymer based on PEG and sheet-like PCys(Bzl) blocks (PCys(Bzl)-*b*-PEG-*b*-PCys(Bzl)) was capable of hydrogelation at a critical gel concentration of 3.5–9.0 wt% [95]. At the same time, a triblock copolymer containing a methyl substituent instead of benzyl in the side chains of PCys, namely PCys(Me)-*b*-PEG-*b*-PCys(Me), does not form a hydrogel up to a concentration of 10 wt%. This is due to the influence of PCys side substituents on the type of non-covalent interactions between the copolymer chains, namely the appearance of additional aromatic interactions between benzyl substituents. In addition, for both polypeptides, it was found that the ability to hydrogelation and the mechanical properties of the resulting hydrogels also depend on such parameters determining the amphiphilic balance as the length of polypeptide fragments and the ratio of blocks.

In addition, it has been revealed that molecular self-assembly, hydrogelation, and mechanical strength of physically cross-linked hydrogels are significantly influenced by the polymer topology (star-shaped or linear), the composition of the hydrophobic polypeptide fragment (PCys(Bzl), PPhe, PLeu, PVal, and PAla), and the polypeptide chain length of amphiphilic copolypeptides [86]. The star-shaped topology, as well as the increase in the number of arms, promoted hydrogelation (a decrease in the critical gelation concentration (CGC)) due to the increase in interchain depot interactions. The presence of benzyl groups in the polypeptide due to additional aromatic (π-π and cation-π) interactions led to an even greater decrease in CGC. Also, increasing the number of arms led to an increase in mechanical strengths, with the highest mechanical strength observed for star-shaped 6-arm PLys-*b*-PPhe explained by the presence of aromatic interactions. The star-shaped polypeptides showed a lower degree of freedom for self-assembly compared to their linear analogs. The predominant conformations for the star-shaped systems studied were the tangle and β-sheet/turn conformations. It was also noted that the hydrogelation of star-shaped PLys-*b*-PPhe was more efficient than that of PLys-*g*-PPhe, which may be due to more efficient packing in the case of the former copolypeptides.

A similar tendency was observed for polypeptides with α-helical structures. Nevertheless, α-helixes play an equally important role in noncovalent gelation processes. For example, star-shape polypeptide P(EG_2_Glu) formed in water a hydrogel mainly due to oriented (parallel or antiparallel) packing of rigid α-helices at low CGC [397]. The polypeptide structure (length and number of arms) influenced both the CGC and the mechanical strength of the hydrogel formed. The most pronounced effect was found when the number of arms was increased. In particular, an increase in the number of arms led to a decrease in CGC and an increase in strength. At the same time, gelation of linear non-ionic diblock-polypeptide based on PEG and P(EG_2_Glu) as well as amphiphilic polypeptide P(EG_2_Glu) with an alkyl tail in water resulted in the formation of hydrogels due to β-sheets of P(EG_2_Glu) [398,399].

Physical gelation at very low concentrations can also be observed for polyelectrolyte-containing block copolymers based on polypeptides, including linear ones. Detailed information on this issue can be found in a recent review [388].

In contrast to physical hydrogels, chemically crosslinked hydrogels are produced by the covalent linking of macromolecule chains. In the case of polypeptides, it can be performed by adding crosslinking agents directly to the polypeptide-containing system or by a preliminary modification of the polypeptide side chains to introduce the required reactive functionality. Covalently linked hydrogels can be made from homopolypeptides, a mixture of different homopolypeptides, copolypeptides, block-copolypeptides, or polypeptide-containing block-copolymers [384,391]. Carbodiimide chemistry, “click” chemistry (alkyne-azide), “click-like” tyrosine/tryptophan-triazolinedione (TAD) conjugation, “thiol-ene” chemistry, enzyme-catalyzed crosslinking, “grafting from” technique, etc., are among the widely used techniques to prepare chemically cross-linked polypeptide hydrogels [27,46,384,400]. However, chemical cross-linking has some drawbacks. First, the use of cross-linking agents and organic solvents may affect the appearance of toxicity or cause side reactions (e.g., with drugs or other components of the hydrogel). Second, cross-linking can involve several steps. In this regard, soft covalent binding under physiological conditions catalyzed by enzymes avoids the disadvantages of chemical modification [401]. However, in this case, enzymes may be entrapped in the hydrogel, which may affect the potential immunogenicity of the biomaterial.

A comparison of the mechanical strengths of physical hydrogels and chemically cross-linked hydrogels indicated that the strength of chemically cross-linked systems is significantly higher [402]. Polypeptide hydrogels can be formed due to both physical forces and chemical crosslinking to combine the advantages of both approaches [16].

Similar to polypeptide-based nanoobjects, polypeptide hydrogels can also exhibit sensitivity to external stimuli. Such stimuli can be temperature, pH, redox environment, enzymes, salts, metals, UV irradiation, or magnetic field [19,388,403]. For example, an injectable self-healing hydrogel formed due to the hydrophobic interactions of four-arm star PEG-*b*-PGlu(*o*NB) was developed by Zhao and coauthors [404]. The hydrophobic moieties contained an encapsulated hydrophobic anticancer pre-drug, DOX. Under UV irradiation, cleavage of the *o*-nitrobenzyl ester bond in the PGlu fragment occurs. As a result, the hydrophobic PGlu(*o*NB) domain transformed into a hydrophilic one, namely PGlu, which weakens drug retention and stimulates the release of loaded DOX. A 4-arm PEG-*b*-PGlu hydrogel pH-sensitive at pH 3 and suitable for subcutaneous or oral delivery and controlled release of protein and peptide drugs was obtained by Bao et al. Using hydrogel loaded with insulin and bovine serum albumin, the release of proteins was investigated in model gastric and intestinal fluids with different pHs. The strong dependance on the pH of the medium is explained by the ongoing processes of deprotonation/protonation of the PGlu carboxyl groups, leading to the sol–gel transition. Increasing pH leads to the deprotection of carboxylic groups and the disassembly of hydrogel, followed by drug release [405].

**Figure 25:**
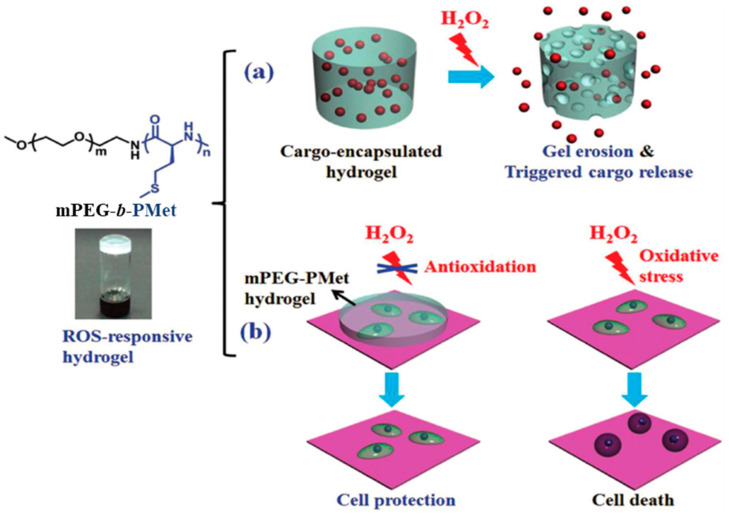
Schematic representation of ROS-responsive hydrogels based on mPEG-*b*-PMet as an oxidation-triggered drug delivery (**a**) and as a protection for cells under oxidative stress system (**b**). Reproduced and adapted with permission of John Willey and Sons, Inc. from [406].

Reactive oxygen species (ROS)-responsive hydrogels based on mPEG-*b*-PMet were obtained as a promising system for drug delivery in the therapy of diseases with oxidative stress, showing cytoprotective properties [406]. It was shown that hydrogen peroxide exposure of the self-assembled hydrogel in aqueous media leads to its erosion due to the oxidation of PMet side chains and the formation of hydrophilic sulfone and sulfoxide groups. Increasing the solubility of the copolymer led to the disintegration of the hydrogel and, in the case of drug loading, to its release (Figure 25).

Both physical and chemical hydrogels can exhibit sensitivity to stimuli. Obtaining permanent polymer networks can be achieved by covalent binding, and a stimulus-mediated bulk phase transition is observed. In turn, reversible polymer networks are usually obtained by physical gelation, and reversible sol–gel or gel–sol phase transitions can occur under stimuli. This ability of hydrogels makes them promising for controlled drug delivery. Stimulus-responsive polypeptide systems capable of in situ gelation are of considerable interest. These are so-called injectable hydrogels, which in their initial state are sols, and after injection into the body, they change to a gel state [19,407].

A thermo-responsive injectable gel capable of self-gelation in the tumor environment, based on mPEG-*b*-P(L-norvaline), was obtained as a promising delivery system for tumor treatment [408]. This physically cross-linked hydrogel allowed controlled release of L-norvaline through cleavage of the peptide bond by tumor enzymes, and in combination with loaded DOX, it demonstrated excellent therapeutic efficacy in vivo (mice).

Physical hydrogels are the first that have been applied for localized drug delivery. However, recently, chemically cross-linked hydrogels based on polypeptides with dynamic covalent bonds (DCB) were discovered for stimulus-responsive delivery [409]. DCB gives hydrogels a sharper sol-gel phase transition when exposed to a stimulus, making them more attractive for use in drug delivery applications. The most studied DCB is the imine bond (or Schiff base, R−N=CH−R’, formed by R’−CHO and R−NH_2_ compounds). A hydrazone bond (R’NH−N=CH−R, formed by R’NH−NH_2_ and R−CHO compounds) structurally similar to the imine bond is also used.

For example, a biocompatible, antibacterial, tissue adhesive, sprayable hydrogel formed by the generation of reversible imine and acylhydrazone bonds between oxidized dextran containing aldehyde groups and charged polypeptides, namely, positively charged 1-(propylthio)acetic acid-3-butylimidazole-modified PLys and/or negatively charged adipate dihydrazide-modified PGlu. The resulting hydrogel exhibited self-healing ability in seconds. In addition, changing the charge density by varying the composition and number of polypeptide fragments can influence the gelation time as well as the biocompatibility of the resulting hydrogel. It was also observed that a higher number of acylhydrazone bonds resulted in a higher modulus and a lower degree of swelling.

In general, much attention has recently been paid to the development of polypeptide-containing polymer hydrogels as a favorable medium for cell functioning, with the aim of applications in cell culture, wound healing, tissue regeneration, and as chemotherapeutic and immunostimulatory agents and/or delivery systems for cancer treatment [16,388,390,400,408,410,411,412,413]. In particular, research is progressing towards stimulus-responsive compositions based on nano-/micro- and macro-peak peptide/polypeptide or peptide/polypeptide-containing materials to enable controlled spatiotemporal drug distribution and combined therapy [414,415,416,417,418,419]. These materials have hydrogels as the matrix and various types of nanoparticles as fillers. Bioactive compounds may be contained in NPs alone or in NPs and hydrogels. In addition, the hydrogels and/or NPs themselves may also be biologically active.

**Figure 26:**
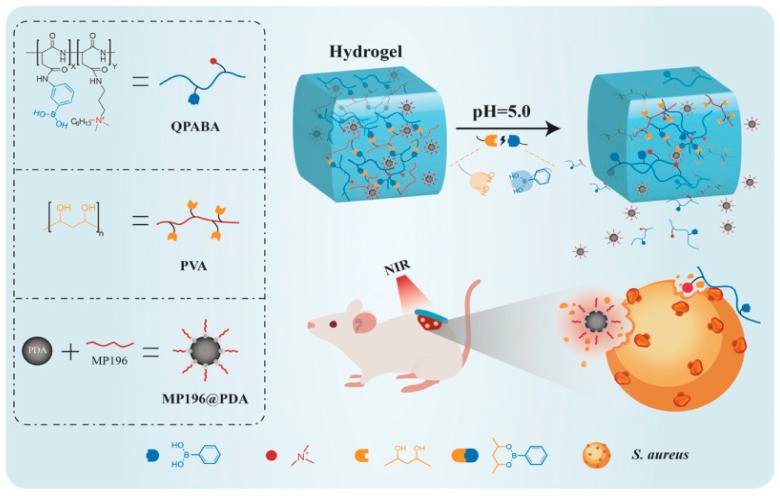
Schematic representation of pH-responsive QPABA/PVA hydrogel with encapsulated NIR-responsive MP196@PDA NPs as a wound treatment material for antibacterial therapy. Reproduced with permission of Elsevier from [411].

For example, Li et al. prepared a multifunctional cationic hydrogel for synergistic cationic, pH-, and NIR-responsive antibacterial therapy in the treatment of bacterial-infected wounds (Figure 26) [411]. The hydrogel matrix was formed by crosslinking quaternary ammonium/boronic acid-modified PAsp (QPABA) and poly(vinyl alcohol) with RWRWRW-NH_2_ peptide (MP196) linked to PDA NPs. The gelation time was 30 s. The presence of phenyl boronic ester bonds provided pH-triggering dissociation under the low pH of the bacterial microenvironment. High in vivo antibacterial efficacy of the developed hydrogel (close to 100%) was achieved in combination with NIR light exposure.

More information on the various factors that play an important role in the polypeptide gelation process, properties, and applications of polypeptide hydrogels can be found in some relevant reviews [16,382,392]. Polypeptide hydrogels, along with hydrogels based on other polymers, are characterized by properties that make them suitable for additive manufacturing of 3D materials, and these properties can be tunable through molecular design [16,395,420,421]. The development of polypeptide-based hydrogels using covalent and non-covalent cross-linking for various 3D printing strategies has recently received special attention. It is noteworthy that the production of materials based on such hydrogels is possible without the introduction of specific additives. The main aspects of this topic have been highlighted in sufficient detail in a recent review [395].

In summary, drugs, genes, biologically active substances, cells, and drug-containing NPs can be incorporated into polypeptide hydrogels by physical distribution or covalent conjugation [390,393,422]. Cargo can be released due to free diffusion or because of the stimulus application [423]. The rate of release can be controlled by (co)polypeptide design and composition, macromolecule length, and the type of hydrophobic side chains [424]. Additional examples of some polypeptides applied to the formation of physical and chemically crosslinked hydrogels, which have not been discussed in the text, are presented in Table 5.

## 5. Application as Drug Delivery Systems

The diversity of polypeptide-based systems allows the delivery of various small drugs and therapeutic macromolecules. In such nanocarriers, drugs can be encapsulated, dispersed, adsorbed, or conjugated [276]. In general, polypeptide-based delivery systems can be considered versatile platforms suitable for the delivery of drugs with different structures, physicochemical properties, and biological properties [431].

### 5.1. Cancer Treatment

Cancer remains the leading cause of death in the world [432]. In this regard, the search for new therapeutic options is urgent. In the last two decades, various delivery systems for cytostatic substances have been actively developed to reduce systemic toxic loads and frequency of administration and to increase the bioavailability of the therapeutic substance [433,434]. Polypeptide-based systems have also received considerable attention in this field due to their biodegradable nature and the possibility of variation in composition and properties over a wide range. The variety of types of polypeptide-based delivery systems considered for cancer treatment are summarized in Table 6.

One of the most popular types of polypeptide-based copolymers considered for the delivery of anti-tumor drugs is PEGylated polypeptides (Table 6). PEGylation is a proven, successful technology for improving the pharmacokinetic profiles of many therapeutic systems [435]. More than 25-years of success in the use of PEGylated drugs and nanoformulations are explained by the biocompatibility and low immunogenicity of this polymer. PEGylation can improve the stability of drug delivery systems, increase circulation time, and mask undesirable properties such as surface cationic charge [436]. Moreover, PEGs are approved by the Food and Drug Administration (FDA) Agency for medical use.

Variation of amino acid side radical functionality allows the preparation of delivery systems with pH and redox-sensitive release (Table 6). In particular, the introduction into the polypeptide of Asp or Glu, with pKa of side carboxyl groups in the region of pH 4, allows the production of NPs capable of drug release in the acidic extracellular environment of tumor tissues (pH 5.5–6.0) [437,438]. The pH-sensitive release provided by anionic amino acids (Glu/Asp) is associated with the protonation of these amino acids, due to which either the ionic stabilization of the particle is disrupted or the ionic interactions with the drug are destroyed. In both cases, this leads to easier release of the drug from the delivery system [369,439,440].

In addition, His is the main amino acid for the preparation of pH-sensitive systems for intracellular delivery [441]. Unlike other basic amino acids such as Lys, Orn, or Arg, the pKa of secondary amine in the imidazole ring of His corresponds to 6, which is close to the pH of early and late endosomes (pH 6.5 and 5.5–6.0, respectively) of cells [442]. Because of this, it is widely used to provide endosomal escape through the so-called “proton sponge effect” [443]. The latter is based on the capture of protons by secondary amines in the acidic environment of the endosome (pH 5–6), which prevents its normal acidification by pumping in more protons and increasing the influx of Cl^−^ and water. Violation of buffering leads to endosome disruption and the release of the drug into the cytoplasm. This approach is widely used to deliver nucleic acids and, sometimes, small drugs with intracellular targets. For example, Hong et al. reported the development of a delivery system based on piperlongumine encapsulated into PEG-*b*-PHis micelles. Piperlongumine is a novel pro-oxidant agent that exhibits cancer-specific cytotoxicity *via* the elevation of intracellular ROS in cancer cells. Using the developed pH-sensitive systems, the authors observed higher cytotoxicity against cancer cells due to an increased intracellular ROS in cancer cells over normal ones. Modification of PEG-*b*-PHis micelles with folic acid demonstrated more specific cellular uptake and anticancer efficacy in folate receptor-positive cancer cells.

**Table 6:** Single drug polypeptide-based delivery systems for cancer treatment.

Polypeptide–Based Copolymer	Delivery form	Drug	Properties	Ref.
PEG-*b*-PGlu/Ca^2+^	pH-responsive nanoparticles	Doxorubicin	*D_H_* = 206 nm; increased release of encapsulated DOX at pH 5.5; Effective inhibition of K7 cells (IC_50_ = 0.15 µg/mL); predominant accumulation of DOX formulation in tumor; the tumor suppression rate in the K7 osteosarcoma-allografted mice was about 80%	[369]
PEG-*b*-P(Glu-*co*-Phe)	Nanoparticles	Doxorubicin	*D_H_* = 140 nm; DOX loading efficiency is almost 98%; A pH-responsive release; high inhibition of A549 compared with free DOX; increased tumor accumulation, reduced toxicity and higher antitumor efficacy compared to free DOX at the same dose	[177]
PEG-*b*-PCys(StBu)	Reduction- responsive micelles	Doxorubicin	Average diameter ˂ 100 nm; low rate of DOX release in buffer and rapid release in GSH-containing media; Selective accumulation of the delivery system in tumors of the orthotopic xenograft mice; reduced distribution in heart	[311,312]
PEG-*b*-P(Tyr-*co*-Tyr(LA))/ cRGD-PEG-*b*-PTyr	Reduction- responsive micelles	Doxorubicin	cRGD-decorated redox-responsive DOX encapsulated micelles; *D_H_* = 45 nm (PDI = 0.04–0.17); reduction-triggered DOX release (up to 80% for 40 h in presence of GSH); effective suppression of human breast tumor growth in mice at 6 mg DOX/kg; absence of visible side effects	[190]
mPEG-*b*-PGlu	pH-responsive nanorods	Doxorubicin	DOX-loaded nanorod size: L = 280 nm, D = 44 nm; release of encapsulated DOX enhanced at pH 5.0 (up to 80% for 120 h); more effective inhibition of cancer cells than with the use of free DOX; sustained release and concentration maintenance in plasma compared to free DOX in vivo	[439]
PLeu-*b*-PEG-*b*-PLeu; P(D,L-Leu)-*b*-PEG-*b*-P(D,L-Leu)	Micelles	Doxorubicin	*D_H_* = 211 nm for levorotatory and 179 nm for racemic polypeptide bearing copolymers; sustained DOX release from both systems; a system with racemic polypeptide blocks demonstrated slower DOX release and enhanced tumor inhibition efficacy in the Saos-2-xenografted female BALB/c nude mice	[93]
PEG-*b*-PLeu	Reduction- responsive micelles	Doxorubicin	*D_H_* = 160 nm; high DOX loading efficiency; reductive-responsive release of DOX due to PEG block cleavage; low cytotoxicity of micelles and effective internalization of the delivery systems into cancer cells	[103]
PAsp-*b*-PEG-*b*-PAsp	pH-responsive micelles	Doxorubicin	Electrostatic DOX loading; *D_H_*~60 nm (PDI = 0.2); loading capacity of 70% (*w/w*) at a drug/polymer ratio of 0.5 at pH 7.0; accelerated release at acidic pH; reduced blood clearance	[94]
PEG-*b*-PGlu-*b*-PHis-*b*-PLeu/ PEG-*b*-LyP-1-peptide/Ca^2+^	pH-responsive micelles	Doxorubicin	LyP-1-peptide: CysGlyAsnLysArgThrArgGlyCys; LyP-1 is an active targeting moiety to gC1qR receptor; pH-sensitive aconityl linkage between PEG and polypeptide fragments contributes to the increased release at pH 5.0; *D_H_* up to 200 nm; effective accumulation in MDA-MB-231 breast cancer cells; profound inhibitory effect in an in vitro metastasis inhibition model	[356]
Tat-ELP-GlyPheLeuGlyCys	Thermally- responsive conjugate	Doxorubicin	Cys-linked DOX conjugate; phase transition range of DOX-polypeptide conjugate was 37–42 °C; a 20-fold increase in cytotoxicity was observed for DOX-polypeptide conjugates when combined with hyperthermia	[444]
PEG-*b*-PLys(NA)	Esterase-responsive vesicles	Doxorubicin	*D_H_*~100 nm; degradation initiated by esterase hydrolysis of phenolic acetate moieties indices vesicles reorganization and promotes DOX release; selective cytotoxicity in the high-esterase-expressive cancer cells over the low-esterase-expressive cells such as normal fibroblasts; effective suppression of tumor growth in BALB/c human cervical tumor-bearing mice	[445]
P(SI-*co*-SI(Lys))-*g*-PAsp	pH-responsive nanogels	Doxorubicin	*D_H_* = 132 nm (PDI = 0.18); DOX conjugation through pH-responsive hydrazone bond; resistance to nonspecific protein adsorption; enhanced drug release under acidic conditions	[440]
PGlu(OBzl)-*b*-PMPC	Micelles	Doxorubicin	*D_H_* = 79 nm (PDI = 0.10); high stability over time; enhanced DOX release at acidic conditions; fast internalization by cancer cells, effective tumor growth inhibition and reduced systemic toxicity in BALB/c mice	[446]
P(Lys-*co*-bAC-*co*-DMMA)	Reduction- and pH-responsive micelles	Doxorubicin	*D_H_* = 80–90 nm; change in micelle surface charge from negative at normal pH to positive at tumor extracellular pH; increased DOX release in a reductive intracellular environment; low cytotoxicity of blank micelles; high growth inhibition against HeLa cells	[343]
His_12_/MOF	pH-responsive hybrid nanoparticles	Doxorubicin	Zn^2+^/2-methylimidazole (Im) MOF; *D_H_* = 100–300 nm depending on Im/Zn^2+^ ratio; pH-responsive drug release at pH 6.3; Enhanced cellular uptake	[447]
PAsp/Fe-Zn	Microrockets	Doxorubicin	Improved stomach delivery, DOX penetration and enhanced retention	[448]
mPEG-*b*-P(Lys-*co*-Lys(Chol))	Nanoparticles	Doxorubicin	*D_H_*~130–230 nm for spherical NPs; chemically conjugated DOX; pH-switchable rod/spherical morphology; temperature/pH dual responsiveness during in vitro release; low cytotoxicity to normal cells; targeting and significant proliferation inhibition of cancer cells	[108]
mPEO-*b*-PHis-*b*-PCys	Micelles	Doxorubicin	*D_H_*~120–210 nm depending on copolymer composition and drug loading; antiproliferative activity of DOX-loaded NPs was comparable to free DOX in three breast cancer cell lines (MCF-7, T-47D, and MDA-MB231)	[107]
P(Glu-*co*-Phe)	Nanogels	Doxorubicin	*D_H_* = 150 nm (PDI = 0.36); more effective cell penetration in comparison with submicron-sized CaCO_3_+Dextran sulfate particles; effective inhibition activity of nanoformulations toward breast cancer cells (MCF7); sustained DOX release in vivo (rats) for 3 weeks after intraperitoneal administration	[449]
mPEG-*b*-P(Glu-*co*-Glu(OEtCl)); mPEG-*b*-P(Glu-*co*-Glu(OEtCl)), crosslinked with Na_2_Se_2_	Micelles; X-ray-responsive nanogels	Doxorubicin	*D_H_*~90–110 nm; X-ray-responsive Se−Se bond in nanogels; enhanced DOX release after X-ray irradiation due to nanogel disintegration; a synergistic effect of chemo- and radiotherapy and fewer side effects toward human A549 lung carcinoma-bearing nude mice	[450]
PEG-*b*-PCys	Reduction- and pH-responsive micelles	Camptothecin	*D_H_* = 73–182 nm depending on composition and *S*-*S*-cross-linking; dual stimuli-triggered intracellular CPT release; improved inhibition activity of nanoformulation at pH 6.5 (for 31.8-fold to 0.61 µg CPT/mL compared to control)	[309]
P(Lys-*co*-Lys-*g*-PEG)-*b*-PPhe	Reduction and pH-sensitive micelles	Camptothecin	*D_H_*~260 nm; PEG-detachable corona; micelles stable in the absence of reducing agents; drug release of 89% at pH 7.4 and 94% at pH 6.5 in the presence of ditiotreitol reducing agent; prolonged blood circulation and enhanced accumulation in tumor in vivo	[83]
γPGlu-*b*-PFK	Nanoparticles	Lonidamine	PFK: ProLys(PheLys)_5_Pro β-sheet peptide; *D_H_* = 17–28 nm depending on PFK amount; high LND loading (73–99%); empty NPs non-cytotoxic to CaCo-2 at low PFK amount (~10 wt%)	[258]
Mal-PEG-*b*-PGlu	Vesicles	Cisplatin	*D_H_*~270 nm; modification of vesicles with thiol-functionalized folic acid; triggered CP release in acidic conditions (pH 5); dose-dependent cytotoxicity towards cancer cells compared to normal cells; higher cellular uptake of FA-modified vesicles	[354]
PMAG-*b*-PGlu(OBzl), PMAG-*b*-Ile and PMAG-*b*-P(Lys-*co*-Phe)	Reduction-responsive micelles and polymersomes	Paclitaxel	*D_H_* = 170–290 nm depending on composition (PDI = 0.22–0.36); PMAG-block was linked through disulfide bonds; absence of cytotoxicity of empty polymer systems to normal cells; low uptake by macrophages by PMAG-containing delivery systems; high inhibition activity of PTX-loaded nanoformulations in different cancer cells	[109,110]
P(Lys-*co*-Phe)/HEP; P[Glu-*co*-Glu(Phe/Ile)-*co*-Glu(Arg/Orn)-*co*-Glu(Glc)]	Nanogels; Nanoparticles	Paclitaxel	*D_H_* = 155–240 nm depending on composition; low cytotoxicity and uptake by macrophages; high inhibition efficiency against breast cancer (MCF-7) and lung adenocarcinoma cancer cells (A549)	[71,110]
P(Glu(OEt))-*b*-PEG-*b*-P(Glu(OEt))	Thermoresponsive hydrogel	Paclitaxel	Thermo-responsive gelation; biocompatibility and elimination of hydrogels within 21 days after subcutaneous injection into mice; effective tumor growth suppression by PTX incorporated hydrogels without evident organ damage	[92]
PLys-*b*-PLeu	Polymersomes	Irinotecan	*D_H_* = 200 nm; loading of irinotecan by pH gradient method; enzyme-triggered drug release; cellular uptake of polymersomes; high stability and low cytotoxicity; Intracellular drug delivery; effective inhibition of CaCo-2 cell growth	[80]
PEOX-*b*-PGlu	Nanoparticles	Irinotecan	*D_H_* = 91 nm drug-NPs conjugated delivery systems; chemical conjugation enhanced CT26 cell death compared to free drug; improved drug stability and solubility	[114]

Abbreviations: IC_50_—half maximal inhibitory concentration; GSH—glutathione; LA—lipoic acid; cRGD—cyclic Arg-Gly-Asp peptide; NA—N-acetoxybenzyl acetate; SI—succinimide; OEt—ethyl ester; Glc—D-Glucosamine. Other abbreviations are provided in the list of abbreviations and in the footer to Table 2.

Cysteines containing thiol groups can facilitate the formation of reduction-responsive disulfide bonds. The latter can link both two polymer chains in copolymers [103,190] and different macromolecules inside a polymer particle [309,311,312]. Disulfide bonds are stable in an extracellular environment low in glutathione (2–20 µM) [451], which is a natural antioxidant regulating many processes as a reducing agent [452]. At the same time, delivery systems containing disulfide bonds are easily disrupted to thiols inside a cell containing a high level of GSH (2–10 mM) [451]. This difference in GSH concentrations is a feature not only of the extracellular and intracellular spaces but also of healthy and tumor tissues. In particular, in vivo studies showed that the GSH concentration in cancer tissues is four times higher than in healthy mouse tissues [453]. Thus, the reduction-responsive destruction of NPs ensures the release of the drug within the target tissues and cells.

Among a variety of anticancer drugs, doxorubicin is the leading molecule used for encapsulation into polypeptide-based NPs (Table 6). Doxorubicin is one of the anthracycline antibiotics possessing high antitumor and antileukemia activity. The demand for the development of encapsulated forms of DOX is due to a number of side effects, such as low selectivity of action, inhibition of hematopoiesis, immunosuppression, and cardiotoxic effects. When administered subcutaneously and intramuscularly, DOX may cause tissue damage and necrosis [454]. In turn, encapsulated DOX can minimize these side effects. In the case of subcutaneous administration, it is possible to create a depot of the drug that provides a sustained therapeutic effect [455]. For other anticancer drugs, several publications are known on the encapsulation of paclitaxel, camptothecin, irinotecan, cisplatin, and lonidamine into polypeptide-based systems (Table 6).

Besides drug delivery, polypeptide-based systems can be successfully used for the preparation of injectable hydrogels for intratumoral radiotherapy [456]. In particular, Schaal et al. reported the development of ELP micelles labeled with the ^131^I radionuclide and forming a hydrogel in situ. Application of the obtained system provided >95% tumor regression in prostate tumors (a PC-3M-luc-C6 tumor model in athymic nude mice).

The majority of failures in cancer therapy are the result of cancer chemoresistance [457]. Drug resistance can be associated with different mechanisms, such as alteration in signaling pathways and drug targets, the presence of molecular efflux pumps, increased DNA repair processes, and cell death inhibition [458,459,460,461]. The use of delivery systems partially solves the existing problems, but not completely. Another way to enhance therapeutic efficacy is to combine different anti-cancer therapeutics to achieve a synergistic action [462,463,464]. The co-delivery approach can be based on the joint administration of different single drug-containing NPs as well as dual drug-loaded delivery systems. However, it has been shown in numerous papers that the best results are observed for co-loaded systems as opposed to the combination of two separate drug nanoformulations [304,465,466]. A combination of two small drugs with different activities [130,467] or a small drug with a gene therapy agent [342] is usually used to obtain dual drug-loaded systems. The polypeptide-based dual drug delivery systems are summarized in Table 7.

Co-loading of two hydrophobic or amphiphilic small drugs is a relatively easy task to realize. For example, Wu et al. reported the preparation of reduction- and NIR-sensitive PCys-*g*-PEG-Lac_5_/Au (Lac—lactose, Au—gold) hybrid NPs for co-delivery of doxorubicin and 6-mercaptopurine, which are cytostatic drugs from the groups of anthracycline antibiotics and antimetabolites (purine antagonists), respectively [130]. The co-delivery of doxorubicin with a pro-oxidant agent (quercetin) [305] or a vascular disrupting drug (combretastatin) [468] allows for high antitumor efficacy due to the combination of a cytostatic action with the induced cell apoptosis or the destruction of blood vessels. Combining a cytostatic drug with an inhibitor of receptors overexpressed in cancers, such as the receptor tyrosine kinase ErbB2 (or Her2/Neu), also results in greater antitumor efficacy in vivo [304].

Unlike small drugs, co-loading a small cytostatic drug with a nucleic acid presents a non-trivial challenge. Loading a small amphiphilic/hydrophobic drug such as PTX or DOX requires a hydrophobic moiety in the copolymer. In turn, gene delivery systems must be cationic to efficiently bind polyanionic nucleic acids. Among amino acids, Lys, Orn, and Arg are used to prepare the gene-binding polypeptide fragment, and His is used to ensure escape from the endosome due to the “proton sponge effect”. The combination of hydrophobic and cationic fragments in one copolymer allows loading a small drug into the hydrophobic core of self-assembled NPs and nucleic acid due to binding to the polycationic part (Table 7). Sometimes the cationic surface can be shielded by a PEG block. siRNAs, or miRNAs, are the most commonly used gene therapeutics for the preparation of anticancer nanoformulations. Both siRNAs and miRNAs are short double-stranded RNAs capable of switching off certain mRNAs and thereby inhibiting the expression of specific proteins overexpressed in cancer cells (e.g., Bcl-2 or VEGF). As with the co-delivery of small drugs, in vivo experiments have shown higher therapeutic efficacy of the cytostatic/nucleic acid dual-drug-loaded systems compared to a mixture of single drug nanoformulations [303,342].

### 5.2. Gene Delivery

Today, gene therapy is recognized as one of the most powerful tools for the treatment of many genetic disorders, as well as cancer and infectious diseases. This kind of therapy is based on the transfer of DNA/RNA into the cells, the replacement of a damaged gene with a healthy copy, or the inactivation of a damaged gene [475,476]. The main feature of nucleic acids is their negative charge, which hinders the penetration of nucleic acid into the cells. Furthermore, they are easily attacked by nucleases in vivo and degraded. Application of gene delivery systems allows for overcoming the existing obstacles.

The development of safe, non-viral systems for intracellular gene delivery is an urgent task in modern biomedicine. Among the existing gene delivery systems, cationic liposomes [477], cationic polymers forming polyplexes with DNA/RNA [478,479], and cationic micelles [480] are the leading systems.

Unlike other cationic synthetic polymers, polypeptide-based systems have been of great interest for nucleic acid delivery for many years due to their biodegradability, biocompatibility, and bioactivity. As mentioned in Section 5.1, cationic polypeptides containing Lys, Orn, and Arg as the main positively charged moieties are very promising for gene delivery. The delivery properties of all cationic systems are based on the electrostatic binding of oppositely charged nucleic acids and further interaction of the system, which has retained its positive charge, with the negatively charged cell membrane.

PLys is one of the most widely characterized gene delivery systems [481,482]. Numerous studies of linear PLys as delivery vectors have shown their low transfection efficiency due to their high cytotoxicity accompanied by their strong complexation ability, especially for high molecular weight PLys [285,483,484]. Moreover, PLys cannot efficiently exit the endosome and degrade within the endosome/lysosome before entering the cytoplasm. In this regard, various approaches to the design of delivery systems have been proposed to improve transfection efficiency. Table 8 shows some recent examples of polypeptide-based delivery systems designed and examined for the delivery of nucleic acids of various structures and lengths (siRNA, mRNA, and pDNA).

In particular, transfection efficiency can be significantly improved by introducing His-units to promote the escape of the delivery system from endosomes [227,310,501], hydrophobic fragments to improve membrane penetration [62,210,499], or negatively charged moieties to weaken binding to nucleic acid and facilitate intracellular release through competitive replacement [66,230,489]. In addition, hydrophobic polypeptides can be modified with non-peptide cationic moieties containing secondary amines in the structure [284,495]. Enhanced gene transfection is also known for PArg and Arg-enriched polypeptides, as PArg is a kind of cell-penetrating peptide [502]. A key role in the uptake of Arg-containing polypeptides is played by the guanidine group, which forms bidentate hydrogen bonds with a phosphate group on the cell membrane surface [503]. After hydrogen bond formation, the ion pair dissociates on the inner side of the membrane in response to the membrane potential after the membrane translocation. For this reason, PArg delivers genetic material into cells more efficiently than the other homopolypeptides, PLys and Phis, of equal length. However, as for PLys, increasing the molecular weight of PArg promotes gene transfection efficiency and, at the same time, cytotoxicity, which severely limits further applications [504]. Thus, the combination of Lys, Arg, and His in the polypeptide structure is one of the most widely used approaches to optimizing the properties of gene delivery systems [72,303,498,499].

In addition, branched polypeptides are also known to exhibit improved gene delivery compared to their linear analogues [505,506,507]. Dendrimers and graft polymers may be the polymers of choice, allowing high transfection efficiency at relatively low molecular weights of the cationic fragments [238,494,498]. Finally, many other specific targeting molecules, such as folate [508] or sugar [210] residues, may also influence cellular uptake *via* receptor-mediated endocytosis.

One of the key issues when using cationic delivery systems in vivo is their slight to moderate cytotoxicity and stability in the blood. To improve these parameters, one possible tool is PEGylation [342,496,509]. Despite the existing positive examples of PEGylation, the development of such systems needs to be balanced between increased stability and circulation time of cationic NPs and a possible decrease in cellular uptake and endosomal release. For example, Kano and his coauthors have studied the grafting of PLys depending on the length of PEG and the grafting density [486]. The authors have found the optimal combination of molecular weights for PLys and PEG chains (*Mw* = 40,000 and 10,000, respectively) as well as grafting density (37%), which allowed successful siRNA retention and protection from siRNA degradation, sustained circulation in the blood, and accumulation in tumors. In contrast, other tested PLys-*g*-PEG compositions failed in some of these properties.

An overview of poly(amino acids) among other types of polymers for gene delivery can also be found in a recent review [510].

### 5.3. Antimicrobial Systems

Bacterial infections pose a significant threat to human health worldwide. Inappropriate use and overuse of antibiotics have led to the rise of antibiotic resistance [511]. In this regard, the development of encapsulated forms of known antibiotics, including those previously withdrawn due to toxicity (e.g., polymyxins), as well as the search for new compounds with antimicrobial properties, are among the priority fields of modern medicine [512]. Currently, many antibiotic delivery systems have been proposed [513,514], including those based on polypeptides [60,515]. Moreover, some peptides [516,517] and polypeptides [518,519] are known to possess inherent antimicrobial properties.

Table 9 provides examples of polypeptide systems obtained by loading an antibiotic into a polypeptide delivery system or polypeptides having their own antimicrobial properties. For instance, Su et al. reported the formation of NPs due to polyelectrolyte interactions between P(*γ*Glu) partly modified with Arg and chitosan modified with Arg [515]. The obtained systems were pH-sensitive and capable of retaining loaded zwitterionic amoxicillin at pH 2.5 and 4.5, but effectively releasing the antibiotic at pH 7.0.

Recently, Iudin et al. and Dvoretskaya et al. have demonstrated the suitability of PGlu-containing NPs to capture positively charged peptide antibiotics (polymyxins) through electrostatic interactions or their covalent conjugation [60,347,520]. In all cases, the physically loaded antibiotics maintained their antimicrobial properties at free-drug levels, while the amide-linked conjugates showed reduced activity. At the same time, all encapsulated forms showed less cytotoxicity to human embryonic kidney cells (HEK 293) compared to free cationic polymyxins.

It is known that polycations can disrupt the membranes of gram-positive and gram-negative bacteria [524]. This property is used in the design of antimicrobial peptides and polypeptides [525,526]. In particular, PLys-containing amphiphiles, such as P(Lys-*co*-Phe) or P(Lys-*co*-Ala), demonstrate their own antimicrobial activity and can be used as NPs, wound covering systems, and coatings for medical use [518,519,522].

### 5.4. Anti-Inflammatory and Antioxidant Systems

Nanoparticle-based drug delivery systems have also found applications in anti-inflammatory therapy. As in the case of other small-molecule drug delivery, efficient delivery of anti-inflammatory drugs can reduce their dosage and improve their therapeutic effect. However, unlike other systems [527], relatively few anti-inflammatory drug delivery systems based on polypeptides have been found to date [214,528,529] (Table 10). They are different in design, drug type, and loading.

For instance, Fan et al. reported the preparation of a thermo-responsive injectable hydrogel based on PEG-*b*-P(Ala-*co*-Gly-*co*-Ile) for local anti-inflammatory therapy with the non-steroid drug naproxen [529]. Recently, Zashikhina et al. developed conjugated dexamethasone (DEX) delivery systems based on amphiphilic polypeptide nanogels with positively (Lys) and negatively (Glu) charged surfaces [214]. The systems were designed for intravitreal injections to provide sustained release of DEX in the vitreous. Cytocompatibility and high mobility of the nanogels were testified for negatively charged Glu-based polypeptides. In turn, cationic NPs showed cytotoxicity and immobilization behavior in the vitreous humor. However, these properties were easily changed after coating the Lys-based nanogels with heparin and recharging the surface.

He-Wei et al. and Bergonzi et al. reported obtaining curcumin delivery systems representing PCL-*b*-PLys micelles [172] and ELP/alginate films [532] for injective administration and topical skin treatment, respectively. Curcumin is a natural polyphenolic molecule with antioxidant and anti-inflammatory properties that has been gaining a lot of attention in the last decade [534]. Composite polypeptide-polysaccharide (ELP/alginate) films demonstrated success in curcumin loading, sustained release, and preservation of antioxidant activity [532].

### 5.5. Protein and Peptide Delivery

Proteins and peptides have gained much attention in recent decades as biopharmaceuticals for the treatment of numerous diseases, such as genetic, immunologic, and endocrine disorders, cancer, infectious diseases, and many others. Today, more than 90% of protein and peptide therapeutics are administered parenterally (44% intravenously, 33% subcutaneously, and 14% intramuscularly) [535]. Despite the known potential high therapeutic efficacy of using peptide and protein drugs, challenges in their use still remain. The main obstacle is related to their structure and susceptibility to biodegradation. As macro- or oligomeric molecules whose primary structure is built from a set of natural amino acids, peptides and proteins can undergo rapid degradation in the presence of proteases and at low pH. This, in turn, reduces their bioavailability. In addition, intracellular penetration of proteins that have targets inside the cell is sometimes limited due to inappropriate protein surface charge or molecular size.

Some progress has been achieved in the use of polymeric materials to protect peptide/protein cargo from loss of bioactivity due to unfavorable conditions during processing and storage, as well as after administration. Unlike commonly studied polymers such as hydrophobic aliphatic polyesters and non-biodegradable poly(vinyl alcohol) widely used for protein/peptide encapsulation [535], polypeptide-based delivery systems are of similar nature and biodegradable, and their properties can be easily adjusted depending on the goal. Since the majority of protein and peptide drugs are water-soluble substances whose stabilization in water is ensured by the functionality of the side chains of hydrophilic amino acids such as Lys, Glu/Gln, Asp/Asn, His, Ser, and Arg, they can be easily loaded into cationic/anionic polypeptide delivery systems through electrostatic interactions. Indeed, the known examples of proteins/peptides loading into polypeptide systems are based on this approach (Table 11).

The study of the biological function of proteins encapsulated in polypeptide NPs showed preservation of protein activity both in vitro and in vivo. For instance, hypoxia-sensitive PEG-*b*-P(Gln((Deta-NBCF)) micelles loaded with cytochrome C induced a greater killing effect in liver cancer cells (HepG2) under hypoxic conditions due to activation of cell death pathways by cytochrome C [536]. In turn, insulin loaded in P(Glu-*co*-Gln(Ts))/PLys interpolyelectrolyte complexes exhibited effective colon permeability and reduced glucose levels in diabetic mice [257].

Zashkhina et al. demonstrated the preservation of biological activity of C-peptide (the connecting peptide; a 31-amino acid polypeptide connecting insulin’s A and B chains in the proinsulin molecule) and its short fragment encapsulated in or conjugated with amphiphilic polypeptide nanogels. It is known that C-peptide possesses a biological function to activate Na^+^/K^+^-adenosine triphosphatase (Na^+^/K^+^-ATPase), whose activity is reduced in erythrocytes and various tissues of Type I diabetic patients. For all tested nanoformulations, loaded/conjugated C-peptide and its fragment effectively stimulated Na+/K+ -ATPase activity in erythrocytes ex vivo [51].

## 6. Summary and Perspectives

The diversity of amino acids allows the synthesis of polypeptides with different compositions and degradability. ROP of α-amino acid NCAs or ROP in combination with other synthetic methods such as azide-alkyne 1,3-dipolar cycloaddition, thiol-ene click chemistry, ROP of lactides/lactones, RAFT polymerization, peptide synthesis, etc., are used to synthesize polypeptides and their copolymers with different architectures such as copolymers, block copolymers, and hyperbranched copolymers. Depending on the composition of the polypeptide or its copolymer, a variety of nanostructures can be obtained through amphiphilic copolymer self-assembly, nanoprecipitation, cross-linking, or polyelectrolyte complexation. Self-assembled NPs (micelles, polymersomes, vesicles, and nanogels) are characterized by lower critical micelle/association concentrations, which provide higher stability of polymeric NPs compared to NPs derived from low-molecular-weight amphiphiles. The physicochemical characteristics of NPs affect their biological properties. Size, surface charge, and stiffness/elasticity are some of the key parameters affecting the rate and mechanism of cellular uptake, cytotoxicity, hemolytic activity, and immunogenicity. In addition to NPs, polypeptide hydrogels are being actively studied as biocompatible systems for local delivery and as injectable drug delivery systems.

Polypeptide-containing materials can provide pH-, reduction-, and temperature-responsive properties both due to their own composition and in combination with corresponding synthetic polymers of other nature. Varying the total charge of polypeptides, the ratio of hydrophilic and hydrophobic properties, and the morphology of the drug delivery system make it possible to co-encapsulate drugs of different natures successfully and with preservation of their activity. These can be both small hydrophilic and hydrophobic molecules and anionic or cationic biomacromolecules (peptides, proteins, and nucleic acids). In addition, polypeptide-based systems have enabled the joining of compounds of different natures, such as hydrophobic small drug molecules and nucleic acids, into one delivery system for combined therapeutic action. Modification of drug delivery systems with special targeting molecules helps to increase the selectivity of drug accumulation in certain biological tissues.

Despite a number of obvious positive properties of polypeptide-based/containing drug delivery systems, there are still some limitations. In particular, the lack of commercially available monomers, the high cost of protected precursors for monomer synthesis, and the requirements for controlled synthesis and anhydrous polymerization conditions limit the scaling of polymer synthesis. The development of stimulus-responsive and targeted delivery systems has improved selectivity and therapeutic efficacy by enhancing on-site localization and release of the drug. However, these delivery systems are often complex in design, requiring a long, complicated, and quite expensive route to obtain them.

A better understanding of structure-property patterns remains crucial for translating synthetic polypeptides into a platform for their introduction into medical practice. Many studies have shown that NPs derived from the same copolymers, differing in block length, amino acid content, or self-assembly method, affect the morphology of NPs, their size, and consequently the biological properties of delivery systems. Therefore, more careful monitoring of physicochemical characteristics, their reproducibility, and systematic studies of their biological effects are required. In addition, most of the developed polypeptides and their copolymers have no physiological functions and, being foreign, may possess immunogenicity. A successful therapeutic formulation that has demonstrated efficacy in vitro should also be studied in vivo. In particular, immunologic and toxicologic properties are also lacking systematic data.

## Figures and Tables

**Table 2:** Some examples of spherical micelles formed from amphiphilic polypeptide-based copolymers.

Block Copolymer	Copolymer *M_n_*	*D_H_* (nm)	PDI	CMC	Ref.
PSer-*b*-PPhe		110–240	0.14–0.23	4 mg/L	[82]
PEG-*b*-PLeu		70–80			[171]
PEG-*S*-*S*-PLeu	6230–6840	160		2.8 mg/L	[103]
PLeu-*b*-PEG-*b*-PLeu	8000	180–210		3–4 mg/L	[93]
PMAG-*b*-PPhe	19,400	190–290	0.07–0.12		[111]
PLys-*b*-PCL	9520	30–60		0.3–0.8 mg/L	[172]
PLys-*b*-PPhe	6100–10,500	490–670	0.07–0.29		[173]
Elastin-like block-copolymers		30–40; 100–200		4–8 µM	[174,175]
PAsp-*b*-PLA	28,000–35,500	30–40		63–360 mg/L (depending on pH)	[176]
PMAG-*b*-P(Glu(OBzl))	7000–29,600	180–260	0.17–0.24		[110]
PEG-*b*-P(Glu-*co*-Phe)	7760	120–140		20 mg/L	[177]
PEG-*g*-PLys-*b*-Phe	6500	80		6 mg/L	[178]
PEO-*b*-PTyr	7700	28		50 mg/L	[88]
PEO-*b*-PLeu	6900	24		14 mg/L	[88]
PEO-*b*-PAsp(OBzl)	16,300	20–130 (depending on conditions)		18 mg/L	[179]
P(Lys-*co*-Lys-*g*-PEG)-*b*-PPhe	1900	260	0.22	2.1 mg/L	[83]

Abbreviations: PDI—polydispersity index; *D_H_*—hydrodynamic diameter; CMC—critical micelle concentration. Other abbreviations are provided in the list of abbreviations and in the footer to Table 1.

**Table 3:** Some examples of polymersomes formed from amphiphilic polypeptide-based block copolymers.

Block Copolymer	Copolymer *M_n_*	Mean *D_H_* (nm)	PDI	CAC	Ref.
PEG-*b*-PGlu(OP)	14,300–21,200	110–180	<0.2	0.07–58 µM (depending on pH)	[102]
PGlu-*b*-PPhe	12,600–39,300	200–300			[81]
PGlu-*b*-PPhe	1600	150		0.11 mg/mL	[18]
PGlu-*b*-PPhe	10,100	1210	0.28	3.5 µM	[85]
PLys-*b*-PLeu	27,000	150–215 (depending on medium)			[80]
PLys-*b*-PLeu	10,200	960	0.24	0.67 µM	[85]
PLys-*b*-PAib	4200–5300	216–398	0.15–0.24		[52]
PMAG-*b*-PIle	9000 10,000	200 260	0.21 0.30		[110]
PB-*b*-PGlu	5200–21,300	106–212		5 µM	[196]
PGlu(OBzl)-*b*- P(IC-AlaAla(OMe))	260,000	7500			[197]

**Table 5:** Examples and properties of some physical and chemically cross-linked polypeptide hydrogels.

Hydrogel Composition	Type of Crosslinking	CGC, wt%	Properties	Ref.
Non-ionic star-shaped P(EG_2_Glu)	Physical (α-helical-based entangled and branched fibrills with width ~ 16–22 nm (TEM, AFM), height 1.1 ± 0.4 nm (AFM), length 100 nm—several µm	1–3 (water)	Shear-thinning and rapid self-healing; *G*’ can be 24–3350 Pa, depending on the composition and concentration of the polypeptide; potential injectable materials for controlled peptide drug release	[397]
Non-ionic PEG-*b*-P(EG_2_Glu)	Physical (β-sheet-based nanoribbon with width 7.5–9.6 nm (TEM) 9.7–13 nm (AFM), height 1.1–1.4 nm (AFM), length—µms)	2—>10 (water)	Temperature-induced sol-to-gel transitions; *G*’ up to 200 Pa; potential injectable drug carriers	[398]
Ionic PAla–*b*-PGlu–*b*-PAla	Physical (β-sheet-based superfibers with width from 0.4 to 8 μm and length > 100 μm (SEM))	4.5 (PBS pH 7.4, NaCl 0.15M)	pH- and thermal-responsive; shear-thinning and rapid self-healing; *G*’ (PBS 0.15M NaCl pH 7.4)~10^6^ Pa	[425]
PGlu-*b*-PEG-*b*-PGlu)-*g*-Chol and PGlu-*g*-*β*-CD	Physical (host-guest crosslinking between β-CD and Chol groups, porous structure with pore diameter of about 40 μm (SEM))	-	Rapid self-healing and non-cytotoxic (ADSC line); *G*’ (alkaline water pH 7.4) can be several hundred—46,000 Pa and degradation (PBS 7.4) from 40 to ~70 days depending on the composition, concentration and ratio of the polypeptides; potential application for tissue engineering	[426]
PEG-*b*-PAla	Physical (combined random coil, α-helix, β-sheet secondary structures and hydrophobic interactions; porous structure with microscale pore size (lyophilized, SEM))	-	Injectable, non-citotoxic (splenocytes and BMDC line) and acceptable biocompatibility in vivo (mice); *G*’ (PBS) ~10^4^ Pa for loaded hydrogel; in vitro and in vivo sustained release antibody (anti-CTLA-4 and anti-PD-1); induction of BMDC maturation, enhanced melanoma-specific CTL response, and high anti-melanoma efficacy in mice for a multi component hydrogel; potential application as a tumor vaccine and antibody local co-delivery system for cancer immunotherapy	[390]
Pluronic-*b*-PLys, pluronic-*b*-PPhe-CHO and BGN@PDA	Chemically cross-linked (Schiff base (imine bonds) between amine of F127-*b*-PLys and aldehyde groups of F127-b-Phe-CHO and BGN@PDA; porous structure (lyophilized, FESEM))	-	Shear-thinning and self-healing (cavity disappearance in 12 h); *G*’ (water) from ~10^1^ to ~10^3^ Pa, depending on the temperature; non-cytotoxic (A375 cancer cells and C2C12 cells); in vitro and in vivo antibacterial capability (*E. coli*, *S. aureus*, and MRSA); excellent photothermal performance (inhibit tumor growth and ablate tumor in vivo); stimulation of angiogenesis and collagen formation; potential application for skin-tumor therapy, anti-infection therapy, wound healing, tissue regeneration, and as an injectable system	[427]
P(Lys-*co*-Trp)/ P(DLys-*co*-DTrp)/their mixture and HMBT	Chemically cross-linked (tryptophan units crosslinked with HMBT (TAD crosslinking); macroporous structure (SEM))	-	Antimicrobial; *G*′~10^5^ Pa for organogels based on non-deblocked polypeptides; slow hydrolytic degradation (8–11% after 7 days, PBS pH 7.4, 37 °C), from rapid (less than 1 day) to long-term (<20% after 6 days) proteolytic degradation (trypsin, PBS pH 7.4, 37 °C) depending on the enantiomeric composition; non-cytotoxic (mammalian cells); potent antimicrobial activity (Gram-positive *S. aureus* and Gram-negative *E. coli*); potential applications for tissue engineering and wound treatment	[428]
mPEG-*b*-PGlu(OEt)	Physical (secondary structures and decrease hydration of PEG block with increasing temperature); porous structure (lyophilized, SEM)	-	Injectable and thermosensitive; *G*′ from ~10^1^ to ~10^3^ Pa depending on the temperature; temperature of sol–gel phase transition from 30 to 36 °C depending on the concentration; in vitro (L929 cells) and in vivo (mice) biocompatibility; sustained release of aPD-L1 and DOX in vitro (PBS 7.4, 37 °C) up to 57% of DOX over 14 days and 28% of aPD-L1 over 3 days; higher anti-melanoma efficacy in mice (intratumoral injection) and longer survival of mice treated with a dual drug-loaded hydrogel compared to single drug loaded hydrogels; potential application for local cancer immunochemotherapy	[413]
mPEG-*b*-PTyr-I^131^	Physical (β-sheet secondary structures and crystallization of PEG blocks at high temperatures; 3D interconnected porous structure (lyophilized, SEM) with continuous network of fibers (TEM))	-	Injectable and thermosensitive; *G*’ from ~2 to ~10^2^ Pa depending on the temperature; radiochemical stability (up to 28 days); biocompatibility (NIH 3T3 normal cells, HepG2 tumor cells) and low hematotoxicity in vivo (mice); stable retention of I^131^ at the site of subcutaneous injection (up to 28 days) and no organ damage in vivo; good local retention of loaded radiosensitizer and its sustained release with peritumoral injection; inhibition of tumor growth (in vivo, mice); potential application for synergistic brachytherapy (co-delivery of iodine-131 and radiosensitizer (SmacN7-R9 peptide))	[429]
HBC-*g*-(DOPA, PLys)	Physical (hydrophobic association and hydrogen interactions between molecular chains) and chemical (DOPA phenols cross-linking); interconnected porous structure with pore size about 90 µm (lyophilized, SEM) and porosity 91% (lyophilized)	-	Reversible temperature-induced sol-gel-sol transitions; *G*’ from ~10^1^ to ~10^3^ Pa depending on the temperature; low-cytotoxic (L929 fibroblasts); hemocompatibility and tissue adhesive (shear strength 672 ± 13 Pa, lap-shear tests, wet porcine skins); in vitro antimicrobial activity (*E. coli* and *S. aureus*); suitable as a platform for 3D cell culture (BMSCs); effective inhibition of infection and inflammation is acompanied by acceleration of wound healing when encapsulated with BMSCs (wound closure is more than 99% after 15 days)	[400]
PGlu-*g*-ADIBO and PGlu-N_3_ with conjugated bioactive peptides (c(RGDfK) and N-cadherin mimetic peptide)	Chemically cross-linked (azide-alkyne 1,3-dipolar cycloaddition) porous structure (lyophilized, SEM)	-	Bioactive; gelation time from 1.5 min to 35 min and *G*’ from ~ 300 to ~ 9400 Pa depending on the concentration; biocompatability (BMSCs); promotes adhesion (BMSCs), proliferation (BMSCs, chondrocytes), and chondrogenic differentiation of cells (BMSCs); proteolytic degradation (PBS pH 7.4, 37 °C, 100% after 2 or 7 days, depending on the enzymes); in vivo (rats) biocompatibility and degradation within 10 weeks after subcutaneous injection	[430]

Abbreviations: *G*’—storage modulus; PBS—phosphate-buffered saline; β-CD—β-cyclodextrin; ADSC—adipose-derived stem cells; BMDC—bone marrow-derived dendritic cells; anti-PD-1—clone RMP1-14 monoclonal antibodies; anti-CTLA-4—clone 9H10 monoclonal antibodies; CTL—cytotoxic T lymphocytes; aPD-L1—anti-programmed cell death 1 ligand monoclonal antibody; TEM—transmission electron microscopy; ROS—reactive oxygen species; SmacN7-R9—SmacN7(AVPIAQK) peptide conjugated with a oligosarginine (to the COOH-terminal of peptide); DOPA—l-3,4-dihydroxyphenylalanine; HBC—hydroxybutyl chitosan; BGN—bioactive glass nanoparticles; PDA—polydopamine; FESEM—field emission scanning electron microscopy; MRSA—methicillin-resistant Staphylococcus aureus; HMBT—hexamethylene-bis-triazolinedione; TAD—triazolinedione; ADIBO—azadibenzocyclooctyne; PGlu-N_3_—azido-modified PGlu; BMSCs—bone marrow mesenchymal stem cells.

**Table 7:** Dual drug polypeptide-based delivery systems for cancer treatment.

Polypeptide–Based Copolymer	Delivery Form	Drug	Properties	Ref.
PEG-*b*-P(Glu-*co*-Glu(Phe))/Ca^2+^	Nanogels	Doxorubicin + 17-AAG	*D_H_* = 63–95 nm (PDI = 0.16–0.20) depending on drug loading; Effective inhibition of the breast cancer cells and selective synergistic anticancer activity against ErbB2-overexpressing breast cancer cell lines; antitumor efficacy of a dual system in vivo surpassed the combination of free drugs	[304]
PCys-*g*-PEG-Lac_5_/Au	Reduction- and NIR-sensitive hybrid nanoparticles	Doxorubicin + 6-mercaptopurine	$\bar{D}$ = 40 nm (TEM), 60 nm (SEM); reduction- and NIR-triggered release; enhanced inhibition effect and lactose-mediated targeting towards HepG2 cancer cells; half maximal inhibitory concentration lower than for individual drugs; Synergistic antitumor effect	[130]
PPhe-*b*-PHis-*b*-PEG	pH-sensitive vesicles	Doxorubicin + quercetin	*D_H_* = 82 nm; pH-sensitive release of both drugs (accelerated at pH 5.5); pro-oxidant QUR enhanced the cytotoxic action of DOX through high oxidative stress and damage to cellular components	[305]
PEG-*b*-P(Ala-*co*-Phe)	Thermo- responsive micelles	Doxorubicin + combretastatin	Thermo-responsive micelles (*D_H_* = 400 nm) were transferred into an injectable hydrogel when heated from 20 to 40 °C; faster release of DOX than CA4 due to their difference in water solubility; dual drug systems improved the apoptosis of tumor cells and had minor damage to normal tissues	[468]
PEG-*b*-PGlu-*b*-PLys decorated with DOXE	Nanoparticles	Doxorubicin + paclitaxel	*D_H_*~56–76 nm; synergistic effect in suppression of A549 cells; low toxicity, high tumor accumulation and antitumor efficiency in vivo	[101]
PGlu-g-PEG/vE decorated with c(RGDfK)	Micelles	Docetaxel + Cisplatin	*D_H_*~46–90 nm; co-loading via hydrophobic and chelation effect; synergistic cytotoxicity and enhanced internalization rate in B16F1 cells (mouse melanoma); long circulation in vivo; Enhanced anti-tumor and anti-metastasis efficacy in the B16F1 melanoma xenograft bearing C57BL/6 mice	[134]
PEG-*b*-PLys-*b*-PLeu	Micelles	Docetaxel + siRNA	*D_H_* = 90 nm; synergistic tumor growth suppression by DTX and siRNA-Bcl-2 micelles due to silencing of the anti-apoptotic Bcl-2 gene as well as enhanced antitumor activity of DTX in the MCF-7 xenografts tumor-bearing nude mice	[342,469]
PArg-*b*-PHis-Stearyl	Micelles	Doxorubicin + miRNA	*D_H_*~170 nm (PDI 0.2); Facilitated endosomal escape of miRNA-34a and release of DOX; synergistic anti-proliferative effect provided by co-delivery of miR-34a and DOX in the DU145 tumor-bearing nude mice; reduced DOX-mediated cardiotoxicity	[303]
PEG-*b*-P(Phe-*co*-Cys)	Reduction- responsive nanogels	Doxorubicin + 1-methyl-D,L-Trp	*D_H_* = 129–137 nm depending on nanoformulation; simultaneous intracellular release of the drugs; combination of DOX to induce immunogenic cell death with immune regulator 1-methyl-DL-tryptophan resulted in the synergistic antitumor effect at reduced DOX dose	[470]
P[Lys-*co*-Lys(Arg)-*co*-Lys(Tyr)]	Nanogels	Paclitaxel + siRNA	*D_H_*~120 nm; effective PTX and siRNA loading; stability of delivery systems; effective inhibition of cancer cells (A549) and GFP silencing in GFP-expressive cancer cells (K562/GFP)	[72]
PEG-*b*-PVal	Injectable hydrogel	Tumor cell lysate + poly(I:C)	Hydrogel-based vaccine for subcutaneous injection based on combination of antigen with agonist of the TLR3 receptor of the immune system; good antitumor efficacy by inducing an immune response of cytotoxic T lymphocytes when administered to mice with melanoma	[471]
PLys-*g*-(PEG-Chol)	Reduction-sensitive nanoparticles	Sorafenib + SIRT7 inhibitor	*D_H_*~300–370 nm depending on loading; effective induction of apoptosis of the liver cancer cells in vitro; high specificity to liver cancer and low toxicity to heart, kidneys, lungs, and liver	[472]
PGlu-*g*-mPEG	Nanoparticles	Garcinia cambogia acid + photosensitizer IR783	*D_H_*~120–200 nm, depending on drug loading; passive accumulation at the tumor and high biocompatibility; low temperature (45 °C) photodynamic anticancer therapy under NIR irradiation	[473]
P(Glu-*co*-DPhe)	pH sensitive nanogels	Paclitaxel + irinotecan; Doxorubicin + irinotecan; Doxorubicin + paclitaxel	*D_H_*~150–250 nm depending on drug combinations; comparison of the release rate from the single and dual drug nanoformulations; drug combinations synergism/antagonism study; comparison of the inhibitory activity towards cancer cells by free drugs and their single and dual drug formulations	[474]

*Abbreviations:* Lac—lactose; Au—gold; NIR—near-infrared; QUR—quercetin; DOXE—deoxycholate; vE—vitamin E (α-tocopherol); c(RGDfK)—cyclic derivative of RGD peptide; SIRT7—a NAD+-dependent class III histone deacetylase. Other abbreviations are provided in the list of abbreviations and in the footer to Table 2, Table 4, Table 5 and Table 6.

**Table 8:** Some polypeptide-based delivery systems for gene delivery.

Polypeptide–Based Copolymer	Delivery Form	Nucleic Acid	Properties	Ref.
P[Glu(OBzl)PEAE)-*co*- Glu(OBzl)MEAM]	Reduction- responsive polyplexes	siRNA	*D_H_*~200 nm; effect siRNA binding; redox-triggered siRNA release due to disulfide-bonds cleavage; facilitated endosomal escape and release of siRNA in the cytosol; reduction of luciferase GL2 expression up to 60% in luciferase-expressive HeLa cells	[284]
oligo(L/D/D,L-Cys-*S*-*S*-CA)	Reduction- responsive polyplexes	siRNA	*D_H_*~100–350 nm; low cytotoxicity to cancer cells (HeLa, HepG2); transfection efficacy was comparable to PEI	[485]
P(Lys-*co*-Lys(Chol))/ branched amylose	Nanogels	siRNA	*D_H_* = 60 nm; enzymatically degradable nanogels; facilitated cellular internalization and effective knockdown of VEGF expression in kidney tumor cells (Renca cells) by anti-VEGF siRNA nanogel complexes	[210]
P(Lys-*co*-Glu-*co*-Phe); P(Lys-*co*-Glu-*co*-Ile);	Nanoparticles	siRNA	*D_H_* = 180–200 nm for optimal polypeptide compositions; facilitated release of siRNA in complex biological medium; efficient VEGF gene silencing in retinal pigment epithelia cells (ARPE-19)	[66]
P(Lys-*co*-Lys(His)-*co*-Phe); P(Lys-*co*-Lys(His)-*co*-Glu-*co*-Phe)	pH-responsive nanoparticles	siRNA	*D_H_* = 150–180 nm (PDI = 0.21–0.22) for optimal polypeptide compositions; pH-responsive siRNA release (enhanced at pH 5.0); efficient cellular uptake and endosomal escape; knockdown of GFP-expression in human breast cancer (MDA-MB-231/GFP cells) was comparable to Turbofect^®^	[227]
PLys-*g*-PEG	Polyplexes	siRNA	PLys was grafted with PEG of various length (2000–10,000); grafting PEG to PLys increased the its lifetime in the bloodstream and accumulation in the tumor without losing its ability to associate with siRNA; copolymer with *M_w_* = 40,000 for PLys and *M_w_* = 10,000 for PEG at 37% grafting demonstrated the best results.	[486]
Fluorinated and guanidinated PGlu(OBzl)	Polyplexes	siRNA	Intratracheally TNF-α siRNA delivery; highly efficient gene knockdown (∼96% at 200 μg/kg siRNA)	[487]
Man-PLys-*b*-PLeu	Polyplexes	siRNA	Thioketal-linked polypeptide; effective crossing blood-brain barrier and accumulation in microglia; reduction of α-synuclein protein aggregates	[488]
PLys-*photo-linker*-HEP	Photo- sensitive polyplexes	siRNA, pDNA	*D_H_* = 100–400 nm depending on PLys molecular weight and PLys/HEP ratio; photo-induced release; low cytotoxicity for several cell lines; enhanced GFP-knockdown by photo-induced anti-GFP siRNA delivery; induction of GFP expression by GFP pDNA delivery	[230]
PLys/HA	Polyplexes	DNA	*D_H_*~140 nm; high DNA loading efficiency (around 95%); efficient transfection into HEK-293T cells (>90%) under optimal conditions combined with low cytotoxicity	[489]
P(Lys-*co*-Ile)	Nanogels	mRNA	*D_H_*~200 nm at optimal polypeptide/mRNA ratio; efficient protein expression after delivery of EGFP-mRNA and fLuc-mRNA transfection by nanogels; delivery was efficient than that observed for bPEI	[62]
PSar-*g*-lipid	Nanoparticles	mRNA	*D_H_*~150 nm; improved target protein section in liver; reduced cytokine secretion in human whole blood indicating low immunogenicity	[490]
PAMAM-PLys dendrimers	Polyplexes	mRNA	*D_H_* ˂ 200 nm at optimized N/P ratio; the presence of α-amino group of an amino acid or introduction of imidazole motifs was not beneficial for transfection activity	[491]
ELP	Polyplexes	pDNA	Delivery of pDNA consisted of four Yamanaka factors (Oct-4, Klf4, c-myc, and Sox2); successful transfection of MEF cells; successful expression of targeted proteins (immunocytochemistry).	[492]
ELP	Polyplex-loaded hollow spheres	pDNA	*D_H_*_~_300 nm; better cell viability than for polyplex alone; high expression of luciferase attributed to providing protection against endosomal degradation.	[493]
ELP-*g*-PTMAEMA	Polyplexes	pDNA	*D_H_*~150 nm; effective pDNA binding; HEK 293 cells transfection by FITC-pDNA/ELP-*g*-PTMAEMA system	[494]
Guanidine-alkyl- functionalized PGlu(OBzl)	Polyplexes	pDNA	Increase in transfection with the elongation of alkyl spacer; transfection comparable with Lipofectamine (LPF200)	[495]
PEG-*b*-PLys-*b*-PGlu(OBzl)	Polyplexes	pDNA	*D_H_*~60–120 nm without cargo depending on Lys content and 110–160 nm in complex with pDNA; highest transfection efficiency for PLys-enriched copolymer (60%) and lowest length of hydrophobic fragment (40%)	[496]
P(Lys-*co*-Lys(His)-*co*-Lys(Cys)-*co*-Phe); P(Lys-*co*-Lys(His)-*co*-Lys(Cys)-*co*-Glu-*co*-Phe)	Reduction and pH-responsive nanoparticles	pDNA	*D_H_* = 60–100 nm depending on Cys content and pDNA loading; improved stability in complex biological medium; reduction-responsive pDNA release; pEGFP-N3 delivery into HEK 293 (30–70% transfection efficacy)	[310]
Lys dendrimers (G3–G5)	Polyplexes	pDNA	*D_H_* = 100–200 nm; transfection efficacy increased with the increase in generation; the higher transfection was 60% compared to control (PEI); the protection capacity of DNA for G5 was comparable with the same for PEI in presence of nuclease; imidazole modified G3 demonstrated properties comparable to unmodified G4	[497]
Lys and Lys-Arg dendrimers (G3–G4)	Polyplexes	pDNA	The superior transfection efficacy for Arg-containing dendrimer, bur lower than control (Lipofectamine L2000); high peptidase resistance and biocompatibility	[498]
Lys and Lys-Gly dendrimers (G3)	Polyplexes	pDNA	Lys dendrimer delivered successfully pDNA into both cancer and normal cells; transfection efficacy comparable with commercial lipofectamine, at less cell damage	[238]
Lys-Arg dendrimers with side chain lipid functionalization	Micelles	siRNA, ssDNA	*D_H_* = 137–457 nm; transfection efficiency of asymmetric peptide dendrimers in various cancer cells surpassed the efficiency of commercial standards (Lipofectamine 2000)	[499]
PLys dendrigraft	Polyplexes	pDNA	Vaccine development; high transfection efficiency; strong cellular immune responses against H9N2 avian influenza virus infection in chickens	[500]
Star-shaped PLys	Polyplexes	pDNA	*D_H_*~120–142 nm depending on PLys arms; PLys grafted from bis-MPA dendritic core (G1-G3) (8–32 PLys arms); successful complexation with pDNA; increased stability in the heparin displacement test of the 32-arm PLys/pDNA complex	[151]

*Abbreviations:* MEAM—*γ*-4-((2-mercaptoethyl)amino methyl); CA—cysteamine; PEAE—*γ*-4-((2-(piperidin-1-yl)ethyl)aminomethyl; VEGF—vascular endothelial growth factor; *M_W_*—weight average molecular weight; *photo-linker*—4-brommethyl-3-nitrobenzoic acid; FITC—fluorescein isothiocyanate; G3-G5—generation 3–5; bis-MPA—bis-(hydroxymethyl)propionic acid. Other abbreviations are provided in the list of abbreviations and in the footer to Table 2, Table 4 and Table 5.

**Table 9:** Polypeptide-based systems with antimicrobial and anti-inflammatory properties.

Polypeptide–Based Copolymer	Delivery Form	Drug	Properties	Ref.
*γ*PGlu-*co*-Glu(Arg)) and CS-*N*-Arg	Polyplexes	Amoxicillin	*D_H_*~200 nm; pH-responsive systems with poor colloidal stability at pH 1.5 and 7.0; effective inhibition of *H. pylori* growth	[515]
P(Glu-*co*-DPhe)	Nanogels	Polymyxin B and E	*D_H_* = 160 nm; low uptake by macrophages and low cytotoxicity; encapsulated systems with MIC equal to free antibiotics	[60]
P(Glu-*co*-Phe)	Nanogels	Polymyxin B	*D_H_* = 280–380 nm depending on drug conjugation amount; conjugated system with MIC higher than for free antibiotic	[520]
PGlu@Ag	Hybrid nanoparticles	Polymyxin B and E	*D_H_* ˂ 210 nm; absence of cytotoxicity against L02 cells up to 250 µg/mL; a synergistic antibacterial effect of polymyxin B and Ag core against *P. aeruginosa*.	[347]
CS/P(α,β-D,L-Asp)	Polyplexes	Isoniazid	*D_H_*~140 nm; encapsulation efficiency in the range of 5.3–5.8%	[521]
[PCL-*b*-P[Phe_12_-*co*-Lys_9_-*co*-Lys(FA)_6_]	Vesicles	AMP: P[Phe_12_-*co*-Lys_9_-*co*-Lys(FA)_6_]	*D_H_* = 300 nm; MIC (*E. coli*, *S. aureus*) = 7–32 µg/mL depending on composition; absence of cytotoxicity against L02 cells up to 250 µg/mL.	[518]
P(Lys-*co*-Phe) and PSar-*b*-P(Lys-*co*-Phe)	Coatings	–	Effective antimicrobial activity against *S. aureus*, *E. coli*, *P. aeruginosa*, and *C. albicans*; antifouling activity to protein and platelet adhesion; biocompatibility with L929 cells	[519]
PEG-*b*-P(Lys-*co*-Ala)	Cross-linked hydrogel	–	Cell adhesion and cell proliferation activities; significant antibacterial activity against *E. coli* and *S. aureus;* system for cutaneous wound healing	[522]
PArgMA/P*β*-AE	Cross-linked hydrogel	–	Adjustable swelling ratio, increasing at pH 5.6; increased cell viability, attachment and proliferation; enhanced antibacterial activities of PβAE against *E. coli* (5.1 times) and *S. aureus* (2.7 times)	[523]
Sulfonium cationic P(D,L-Met)	Solution	–	Broad spectrum of antibacterial activity (*E. coli*. *P. aeruginosa*, *S. epidermidis,* methicillin-resistant *S. aureus*); increase in MIC with the polymer chain elongation; biocompatibility with mammalian cells	[362]

Abbreviations: MIC—minimum inhibitory concentration; Ag—silver; AMP—antimicrobial polypeptide. Other abbreviations are provided in the list of abbreviations and in the footer to Table 2.

**Table 10:** Polypeptide-based systems with anti-inflammatory and antioxidant properties.

Polypeptide–Based Copolymer	Delivery Form	Drug	Properties	Ref.
PLys-*b*-PThr	Nanogels	Recombinant TRAIL protein	*D_H_*~300 nm, PDI = 0.21; combination of antimicrobial properties of polypeptide with anti-inflammatory properties of the protein drug to treatment of the *K. pneumoniae*-caused sepsis; Intraperitoneal injections prevented mice lung and renal injury, as well as intratracheal sepsis	[528]
P(Lys-*co*-Phe), P(Glu-*co*-Phe), P(Lys-*co*-Phe)/HEP	Nanogels	Dexamethasone	Conjugated systems with D_H_ of 200–290 nm depending on polypeptide composition; negative nanoparticles are able to migrate in the vitreous and are non-toxic to retinal cells; release ester-linked drug within a week in vitreous/buffer medium (50/50, *v*/*v*)	[214]
mPEG-*b*-PLys	Reduction- responsive micelles	Dexamethasone	Disulfide-bonded conjugates with *D_H_* of 66–76 nm; enhanced accumulation in murine colorectal cancer model (CT26) compared to free DEX; increased the CD8+ T cell infiltration and the M1 over M2 macrophage ratios	[530]
PNIPAM-*b*-PGlu	Thermo- responsive micelles	Dexamethasone	*D_H_*~85 nm; thermo-responsive micelles forming microneedles; reduction in mechanical strength of microneedles after drug loading; effective delivery of the DEX to the sclera	[112]
PEG-*b*-P(Ala-*co*-Gly-*co*-Ile)	Thermo- responsive hydrogel	Naproxen	Biodegradable hydrogel with gelation dependent on polymer concentration, length of polypeptide segment; drug release within six days; suitable as injectable delivery systems	[529]
PHis/Ceria	Nanocages	Acetylcholine chloride + inhibitor of the activin receptor-like kinase (SB431542)	Good biocompatibility with the eye; enhanced corneal penetration and effective pH-responsive release of drugs caused by tissue injury/inflammation	[531]
PCL-*b*-PLys	Micelles	Curcumin	$\bar{D}=$ 33–57 nm depending on loading; High loading capacity (up to ~9.5%)	[172]
ELP/alginate	Films	Curcumin	Sustained release up to 10 days; high cytocompatibility with human dermal fibroblasts; presence of antioxidant activity of the material	[532]
PLys-*g*-mesoporous silica	Nanoparticles	Quercetin	Effective drug loading and enhanced release at pH 3	[533]

Abbreviations are provided in the list of abbreviations and in the footer to Table 2.

**Table 11:** Some polypeptide systems designed for protein and peptide delivery.

Polypeptide—Based Copolymer	Delivery Form	Drug	Properties	Ref.
P(Lys-*co*-Ala)-*b*-PLX-*b*-P(Lys-*co*-Ala)	Thermoresponsive Nanogels	BSA	*D_H_*~80 nm at 30 °C, ~110 nm at 40 °C; biodegradation in presence of elastase; Protein sustained release within 9 days	[256]
P(Glu-*co*-Gln(Ts))/PLys	Polyplexes	Insulin	*D_H_* = 220–350 nm depending on composition; release of insulin at pH 6.8 (intestinal) and hindering the drug leakage at pH 1.5 (stomach); improved penetration through the CaCo-2 cell layer; reduction of the glucose level in diabetic mice	[257]
PEG-*b*-P(Gln((Deta-NBCF))	Micelles	Cytochrome C	Hypoxia-responsive system; high protein loading content and stability at normoxic condition; great killing effect to liver cancer cells (HepG2) under hypoxic condition	[536]
P(Glu-*co*-DPhe)/CS	Nanogels	Neuropeptide (β-endorphin fragment 9–19)	*D_H_*~200–300 nm; encapsulation efficacy of 76%; release of 26 and 16% for 24 h in simulated intestinal fluid for neat polypeptide nanogels and their chitosan-coated forms, respectively	[537]
P(Lys-*co*-DPhe); P(Glu-co-DPhe)	Nanogels	C-peptide or its fragment	Encapsulation or conjugation; High peptide loading; *D_H_* = 80–190 nm; stability over time in PBS; high biological effects by stimulating Na^+^/K^+^—ATPase activity in erythrocytes	[51,538]

Abbreviations are provided in the list of abbreviations and in the footer to Table 2.

## Data Availability

The data can be shared up on request.

## Abbreviations

*Amino acids and their derivatives*: Aib—α-aminoisobutyric acid; Ala—L-alanine; Arg—L-arginine; Asn—L-asparagine; Asp—L-aspartic acid; Asp(OBzl)—*β*-benzyl-L-aspartate; Cys—L-cysteine; Cys(SCbz)—S-benzyloxycarbonyl-L-cysteine; Cys(S*o*NB)—*S*-(*o*-nitrobenzyl)-L-cysteine); Gln—L-glutamine; Gln(Deta-NBCF)—*γ*-(diethylene triamine-*g*-4-nitrobenzyl chloroformate)-L-glutamine; Gln(Ts)—*γ*-tosyl-L-glutamine; Glu—L-glutamic acid; Glu(OBzl)—*γ*-benzyl-L-glutamate; Glu(*o*NB)—γ-*o*-nitrobenzyl-L-glutamate; Glu(OSu)—*γ*-succinimide ester of glutamic acid; Gly—L-glycine; His—L-histidine; IC-AlaAla(OMe)—L-isocyanoalanyl-L-alanine methyl ester; Ile—L-isoleucine; Leu—L-leucine; DLeu—D-leucine; Lys—L-lysine; Lys(Z)—*ε*-carboxybenzyl-L-lysine; Lys(Boc)—*ε*-tert-butoxycarbonyl-L-lysine; Lys(*o*NBC)—*ε*-*N*-(o-nitrobenzyloxycarbonyl)-L-lysine; Met—L-methionine; Orn—L-ornithine; Phe—L-phenylalanine; DPhe—D-phenylalanine; Phe(DH)—3,4-dihydroxyphenylalanine; Pro—L-proline; Sar—sarcosine; Ser—L-serine; Thr—L-threonine; DTrp—D-triptophan; Tyr—L-tyrosine; Tyr(Bzl)—*O*-benzyl-L-tyrosine; Val—L-valine; DVal—D-valine.

*Polymers*: *γ*PGlu—poly(γ-L-glutamic acid); bAC—*N*,*N*-bis(acryloyl)cystamine; CS—chitosan; ELP—elastin-like polypeptides; HA—hyaluronic acid; HEP—heparin; OEG—oligo ethylene glycol; P—poly; P(D,L-Leu)—poly(D,L-leucine); PDLys—poly(D-lysine); P(EG_2_Glu)—P(*γ*-(2-(2-methoxyethoxy)esteryl)L-glutamate); P(IC-AlaAla(OMe))— poly(L-isocyanoalanyl-L-alanine methyl ester); PAMAM—polyamidoamine; PB—polybutadiene; PCL—poly(*ε*-caprolactone); PCys(StBu)—*S*-tert-butylmercapto-L-cysteine; PDA—polydopamine; PMPC—poly(2-methacryloyloxyethyl phosphorylcholine); PE—polyethylene; PEG—poly(ethylene glycol); mPEG—poly(ethylene glycol) monomethyl ether; PEI—poly(ethylene imine); PEO—poly(ethylene oxide); PGlu(OP)—poly(*γ*-propargyl-L-glutamate); PGlu(EEO_2_)—poly(γ-(2-(2-ethoxyethoxy)ethyl)-L-glutamate); PIPOX—poly(2-isopropyl-2-oxazoline); PEOX—poly(2-ethyl-2-oxazoline); PLA—polylactide; PLGA—poly(lactic-*co*-glycolic acid)); PLX—poloxamer; PMAG—poly(2-deoxy-2-methacrylamido-D-glucose); PNIPAM—poly(*N*-isopropylacrylamide); PTMAEMA—P[2-(methacryloyloxy)ethyl]trimethylammonium; PTMC—poly(trimethyl carbonate); Tat—cell penetrating peptide fragment from HIV-1 Tat protein.

*Drugs and biologically active agents*: 17-AAG—17-allylaminodemethoxygeldanamycin; CA4—combretastatin; Chol—cholesterol; CP—cisplatin; CPT—camptothecin; DACHPt—1,2-diaminocyclohexane-platinum(II); DEX—dexamethasone; DNA—deoxyribonucleic acid; pDNA—plasmid DNA; DOX—doxorubicin; DOXꞏHCl—doxorubicin hydrochloride; DTX—docetaxel; LND—lonidamine; RNA—ribonucleic acid; miRNA—microRNA; mRNA—messenger RNA; siRNA—small interfering RNA; ssDNA—single-stranded DNA; poly(I:C)—polyinosinic:polycytidylic acid; PTX—paclitaxel.

*Other compounds*: DMMA—dimethylmaleic anhydride; DOXE—deoxycholate; FA—folic acid; GSH—glutathione; Lac—lactose; Mal—maleimide; MOF—metal-organic framework; NA—N-acetoxybenzyl acetate; SI—succinimide; SIRT1—silent information regulator 1; vE—vitamin E (α-tocopherol).
